# First-in-human randomized controlled trial of an oral, replicating adenovirus 26 vector vaccine for HIV-1

**DOI:** 10.1371/journal.pone.0205139

**Published:** 2018-11-14

**Authors:** Kathryn E. Stephenson, Michael C. Keefer, Catherine A. Bunce, Doreen Frances, Peter Abbink, Lori F. Maxfield, George H. Neubauer, Joseph Nkolola, Lauren Peter, Christopher Lane, Harriet Park, Carl Verlinde, Angela Lombardo, Christopher Yallop, Menzo Havenga, Patricia Fast, John Treanor, Dan H. Barouch

**Affiliations:** 1 Center for Virology and Vaccine Research, Department of Medicine, Beth Israel Deaconess Medical Center, Boston, Massachusetts, United States of America; 2 Harvard Medical School, Boston, Massachusetts, United States of America; 3 Ragon Institute of MGH, MIT and Harvard, Cambridge, Massachusetts, United States of America; 4 University of Rochester Medical Center, School of Medicine and Dentistry, Rochester, New York, United States of America; 5 International AIDS Vaccine Initiative, New York, New York, United States of America; 6 Batavia Biosciences B.V., Leiden, The Netherlands; Rush University, UNITED STATES

## Abstract

**Background:**

Live, attenuated viral vectors that express HIV-1 antigens are being investigated as an approach to generating durable immune responses against HIV-1 in humans. We recently developed a replication-competent, highly attenuated Ad26 vector that expresses mosaic HIV-1 Env (rcAd26.MOS1.HIV-Env, “rcAd26”). Here we present the results of a first-in-human, placebo-controlled clinical trial to test the safety, immunogenicity and mucosal shedding of rcAd26 given orally.

**Methods:**

Healthy adults were randomly assigned to receive a single oral dose of vaccine or placebo at 5:1 ratio in a dosage escalation of 10^8 to 10^11 rcAd26 VP (nominal doses) at University of Rochester Medical Center, Rochester, NY, USA. Participants were isolated and monitored for reactogenicity for 10 days post-vaccination, and adverse events were recorded up to day 112. Rectal and oropharyngeal secretions were evaluated for shedding of the vaccine. Humoral and cellular immune responses were measured. Household contacts were monitored for secondary vaccine transmission.

**Results:**

We enrolled 22 participants and 11 household contacts between February 7 and June 24, 2015. 18 participants received one dose of HIV-1 vaccine and 4 participants received placebo. The vaccine caused only mild to moderate adverse events. No vaccine-related SAEs were observed. No infectious rcAd26 viral particles were detected in rectal or oropharyngeal secretions from any participant. Env-specific ELISA and ELISPOT responses were undetectable. No household contacts developed vaccine-induced HIV-1 seropositivity or vaccine-associated illness.

**Conclusions:**

The highly attenuated rcAd26.MOS1.HIV-Env vaccine was well tolerated up to 10^11 VP in healthy, HIV-1-uninfected adults, though the single dose was poorly immunogenic suggesting the replicative capacity of the vector was too attenuated. There was no evidence of shedding of infectious virus or secondary vaccine transmission following the isolation period. These data suggest the use of less attenuated viral vectors in future studies of live, oral HIV-1 vaccines.

**Trial registration:**

ClinicalTrials.gov NCT02366013.

## Introduction

The development of an HIV-1 vaccine remains a critical goal in the effort to decrease HIV-1 incidence and end the worldwide epidemic. However, it remains unclear how to increase the potency and durability of HIV-1-specific immunity following vaccination. One strategy to improve vaccine-induced immune responses is to use live, replicating vectors to express HIV-1 antigens[1]. Live-attenuated viral vaccines are effective in both humans and animals and include some of the most widely-used licensed vaccines in the world[2]. Live vectors expressing HIV-1 antigens might approximate a live, attenuated HIV-1 vaccine, while also providing a better safety profile, particularly when delivered via a mucosal route.

**Fig 1:**
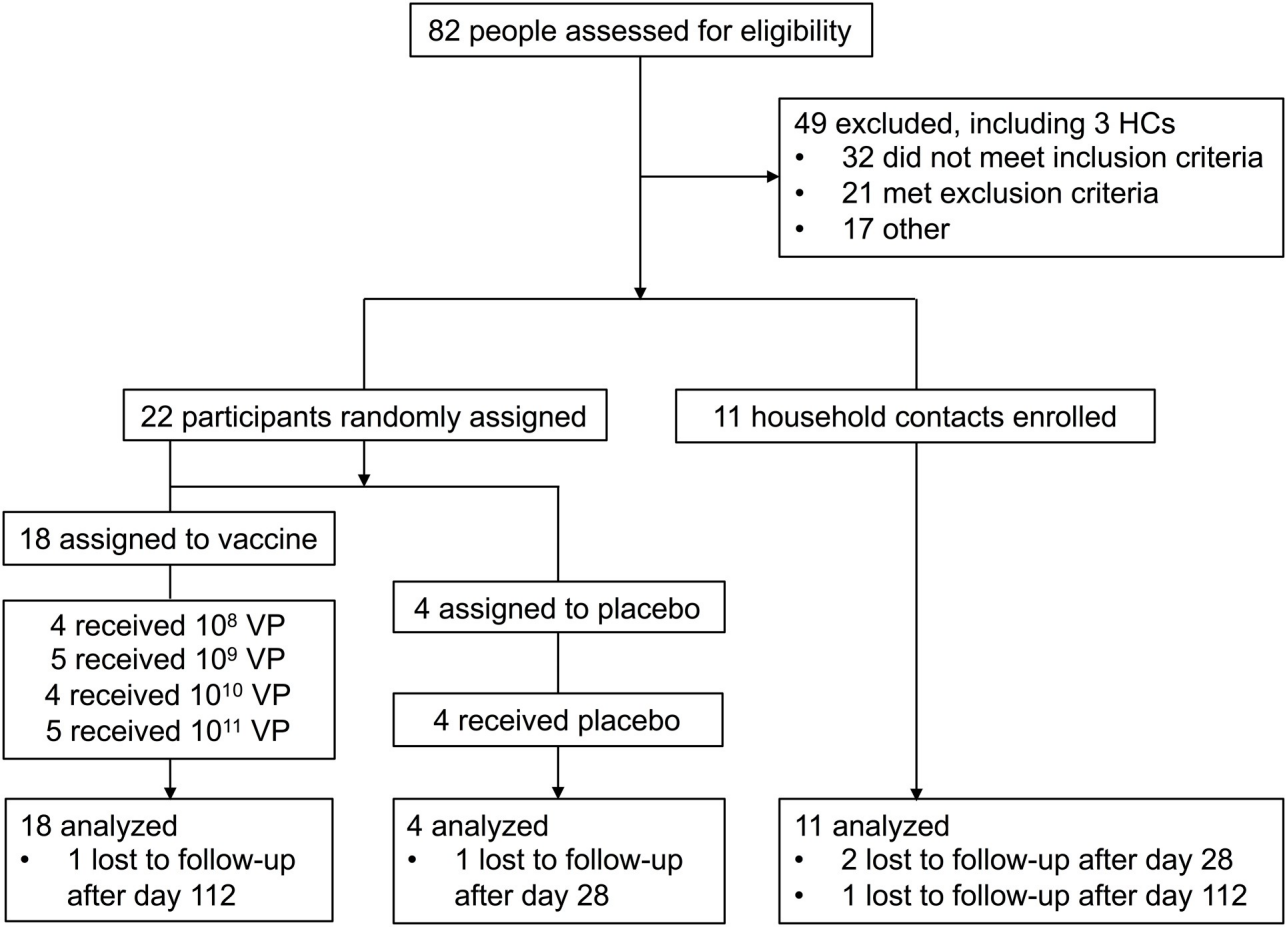
Trial profile. HC, household contact.

To test this hypothesis, we produced a replication-competent adenovirus serotype 26 (rcAd26) vector by deleting most of the E3 and part of E4 genes of wild-type Ad26 and inserting a mosaic HIV-1 Env transgene (Fig 1)[3]. The resulting vaccine, rcAd26.MOS1.HIV-Env, was highly attenuated compared to wild-type Ad26, showing a 4.4-log reduction in replicative capacity in the duodenal cell line HuTu80. The highly attenuated nature of the rcAd26 vaccine was likely due to deletions in E3 and E4, which may be necessary for optimal Ad26 replication, and the addition of the Env transgene, which reduces replication efficiency. Nevertheless, the rcAd26 vaccine elicited humoral and cellular immune responses when delivered intramuscularly in mice and non-human primates, even though it does not replicate in these models. For human studies, we lyophilized and formulated the vaccine as Acryl-EZE enteric-coated capsules for oral administration.

Here we report on the findings of a phase 1, first-in-human, dose-escalation, placebo-controlled clinical trial (NCT02366013) to test the safety, immunogenicity and mucosal shedding of the oral rcAd26.MOS1.HIV-Env vaccine in healthy HIV-1-uninfected adults aged 18–50 years old.

## Methods

### Study design

This study was a single-center, randomized, double-blind, placebo-controlled, dose-escalation trial to evaluate the safety, immunogenicity, and mucosal shedding of a single dose of rcAd26.MOS1.HIV-Env (rcAd26) in HIV-uninfected participants. The Research Subjects Review Board at University of Rochester Medical Center approved the protocol on 29 Dec 2014, and participant recruitment began on 20 Jan 2015. Written informed consent was obtained from each participant. Study participants were healthy and at low risk for acquiring HIV as per standard criteria. The study was registered at ClinicalTrials.gov (NCT02366013) on 04 Feb 2015 and registration was approved after revisions on 18 Feb 2015. First enrollment was on 18 Feb 2015. Final participant follow up visit was on 20 Jun 2016.

Groups 1–4 received sequential 10-fold increases in vaccine dose from 10^8 to 10^11 VP (nominal doses) (Fig 1). Participants within each group were randomly assigned to receive vaccine or placebo at a 5:1 ratio. Randomized treatment assignments were generated by the Data Management Center (DMC). After successful randomization, an allocation number was provided. The DMC created the randomization table and the sponsor monitored the implementation of this process. The allocation number was referenced against a confidential list provided to an unblinded on-site pharmacist to determine the assignment/treatment allocation.

To minimize the risk of secondary vaccine transmission, all participants remained in individual rooms in an isolation facility until Day 9 after dosing; each group was admitted together as a cohort. All study products were given as enteric-coated capsules to be swallowed with a glass of non-carbonated water. Systematic safety assessments were conducted throughout the study, and participants were followed for 1 year (see S1 Protocol for full study protocol). Medical monitoring was provided by a Protocol Safety Review Team and an independent Safety Monitoring Committee. Peripheral blood was collected to determine anti-HIV and anti-vector immunity on Day 0, 17, 21, 28, 112, 240, and 365, and to detect systemic vaccine replication on Day 7. Rectal Weck-Cel^®^ sponges were collected to determine mucosal immunogenicity on Day -2, 17, 28, 112, 240, and 365. Rectal and oropharyngeal swabs were collected to determine vaccine shedding on Day -2, 1, 2, 3, 4, 5, 6, 7, 8, 9, 12, 17, 21, and 28.

Household and sexual contacts (Household Contacts, HCs) were enrolled in the study as well. Only participants with 2 or fewer adult HCs were eligible to enroll. HCs were healthy adults at low risk for HIV acquisition; all HCs provided written informed consent. HCs had no contact with participants until the isolation period was completed. Following participant discharge from isolation, HCs were monitored for intercurrent illnesses, adverse events, sero-conversion to HIV and anti-vector immunity until 8 months following enrollment. HCs were also asked to report illnesses that might be due to adenovirus to study staff at any point in the study, e.g., fever, conjunctivitis, gastroenteritis, cystitis or upper respiratory infection.

### Vaccine

**Fig 2:**
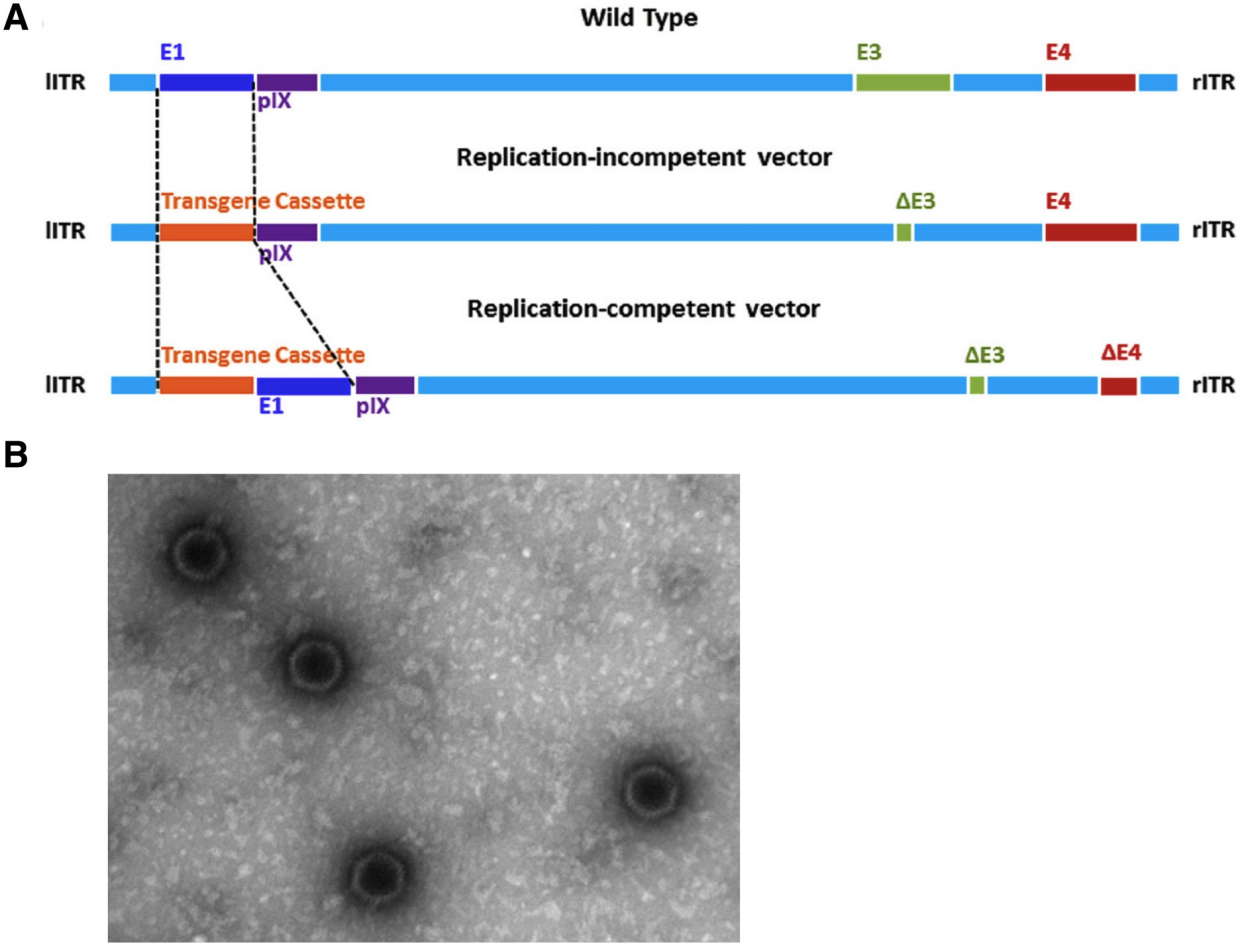
Oral, replicating adenovirus 26 vector vaccine for HIV-1. (A) Schematic showing the construction of the replication competent adenovirus serotype 26 (Ad26) vaccine vector from wild-type Ad26. A transgene cassette consisting of a mosaic HIV-1 Env gene (Mos1 Env) was inserted to create the rcAd26.MOS1.HIV-Env (rcAd26) vaccine. lITR, left inverted terminal repeat; rITR, right inverted terminal repeat[3]. (B) Intact rcAd26 vaccine from dissolved lyophilized capsule as seen by electron microscopy.

The rcAd26.MOS1.HIV-Env vaccine candidate is a replication competent human adenovirus serotype 26 (rcAd26), used as a vaccine vector to deliver a transgene (Mos1-Env) consisting of antigens from the HIV-1 envelope (Fig 2). The recombinant rcAd26 virus has most of the E3 and part of the E4 genes (open reading frames 1–4) deleted to allow the Mos1ENV transgene to be inserted. Bulk rcAd26.MOS1.HIV-Env virus vector was manufactured by Batavia Biosciences (The Netherlands) propagated in PER.C6^®^ cells and then purified, concentrated and sterile filtered.

Capsule formulation was conducted at PaxVax Inc. (San Diego, USA) with rcAd26.MOS1.HIV-Env virus vector provided by Batavia Biosciences. The bulk virus vector was formulated with stabilizers and then lyophilized prior to filling into capsules. The placebo and the low dose 10^8 VP were filled in the smaller Size 3 capsules, while the high dose 10^10 VP dose was filled in larger Size 1 capsules. The capsules were then coated with Acryl-EZE enteric coating. Placebo capsules were sucrose-filled HPMC Size 3 capsules enteric coated with Acryl-EZE. Participants who received a total dose of 10^9 VP were administered 10 Size 3 capsules containing 10^8 VP each. Participants who received a total dose of 10^11 VP were administered 10 Size 1 capsules containing 10^10 VP each.

### Immunogenicity studies

All immunogenicity assays were performed in a blinded fashion under Good Clinical Laboratory Practice (GCLP) conditions. Ad26-specific neutralizing antibodies (NAbs) were assessed by luciferase-based adenovirus neutralization assays as previously described[4,5]. Serum IgG binding to the vaccine-matched Mos1 Env antigen was assessed by direct enzyme-linked immunosorbent assays (ELISAs) [6]. Interferon (IFN)-gamma enzyme-linked immunospot assays (ELISPOTs) were performed to assess Env-specific cellular immune responses using a pool of overlapping Mos1 Env peptides[6]. Criteria for positive Ad26 NAb responses were titers ≥ 18 and for Env ELISA responses was an absorbance >2-fold over mean background values and absolute optical density (O.D.) > 0.2. Criteria for ELISPOT positivity were ≥ 55 spot-forming cells (SFC) per million peripheral blood mononuclear cell (PBMC) and at least 3x greater than background.

### Viral shedding assessment

Rectal swabs and plasma were assessed for rcAd26.MOS1.HIV-Env by real-time (rt)PCR for the Ad26 vector hexon region to allow for quantitation of vector. Criteria for PCR positivity was detection of adenovirus at fluid concentrations of 1000 VP/mL or higher. If Ad26 DNA was detected in a clinical sample, adenovirus cultures were also performed to evaluate for the presence of infectious virus. Positive outgrowth cultures were assessed for presence of the Envelope insert by PCR over the transgene cassette to distinguish between vaccine vector or community-acquired Ad26 infection. A single individual on the study team remained unblinded to viral shedding results from day 28. If a participant’s adenovirus PCR remained detectable at day 28, weekly adenovirus PCR was to be performed until the PCR was negative.

### Statistical methods

The number of participants was chosen for this study to provide a preliminary safety and immunogenicity assessment. The sample size was not based on formal hypothesis testing considerations, but was within the range of participants (i.e., 20–80) recommended in the Code of Federal Regulations (CFR 312.21) for first-in-human product Phase 1 evaluations. Placebo recipients were included for blinding and safety purposes and to provide additional control specimens for immunogenicity assays. In particular, if no events were observed in a group of 4 or 5 participants, the upper 95% Clopper-Pearson confidence limit on the true rate of events would be 60.2% and 52.2%, respectively.

All analyses are based on the intent-to-treat principle including all participants in the group to which they were randomized. Summaries of responses are presented with geometric mean titers (GMTs) for the Ad NAb, HIV-1 ELISA and HIV-1 ELISPOTs, all with associated 95% confidence intervals (CIs).

## Results

### Study conduct and population

Screening began on 20 January 2015 and a total of 82 individuals were screened (Fig 1). Enrollment commenced on 18 February 2015 and was completed on 24 June 2015. Twenty-two (22) participants and 11 household contacts were enrolled in the study. The baseline characteristics of the participants are shown in Table 1. There were 49 individuals who were deemed ineligible for the study. The primary reason for screen failure was inability of the participant to accommodate the study schedule. Two participants and three household contacts were lost to follow up and thus, were terminated from the study early. All 22 participants were administered study product with the last administrations for Group 4 occurring on 23 June 2015. The last participant was seen on 20 June 2016.

**Table 1:** Characteristics of the participants at baseline.

Variable	Group 1	Group 2	Group 3	Group 4	Placebo	Household Contacts	All Participants
No. of participants	4	5	4	5	4	11	33
Age							
Mean	24.8	26.2	32.0	28.6	30.8	35.7	30.8
Range	21–29	20–32	25–46	21–46	24–47	18–57	18–57
Gender							
Female—no. (%)	0	2 (40.0)	1 (25.0)	3 (60.0)	2 (50.0)	6 (54.5)	14 (42.4)
Male—no. (%)	4 (100.0)	3 (60.0)	3 (60.0)	2 (40.0)	2 (50.0)	5 (45.5)	19 (57.6)
Race							
Asian—no. (%)	0	0	0	0	0	1 (9.1)	1 (3.0)
Black—no. (%)	2 (50.0)	1 (20.0)	3 (75.0)	0	2 (50.0)	1 (9.1)	9 (27.3)
Black, American Indian—no. (%)	0	0	0	1 (20.0)	0	0	1 (3.0)
White—no. (%)	2 (50.0)	3 (60.0)	1 (25.0)	4 (80.0)	2 (50.0)	8 (72.7)	20 (60.6)
White, Black—no. (%)	0	1 (20.0)	0	0	0	1 (9.1)	2 (6.1)
Ethnicity							
Hispanic or Latino	0	1 (20.0)	0	0	0	0	1 (3.0)
Not Hispanic and Not Latino	4 (100.0)	4 (80.0)	4 (100.0)	5 (100.0)	4 (100.0)	11 (100.0)	32 (97.0)
Height (cm)							
Mean	179.3	171.6	168.3	173.3	174.0	N/A	173.2
Range	171–188	155–184	163–181	165–186	162–188		155–188
Weight (kg)							
Mean	83.3	72.4	75.5	87.0	89.3	N/A	81.3
Range	73–90	53–93	68–83	63–107	72–112		53–112
BMI (kg/m^2^)							
Mean	26.0	24.5	26.7	28.9	29.3	N/A	27.0
Range	22.5–28.7	20.1–28.4	25.3–29.0	22.6–35.3	26.3–33.4		20.1–35.3
Study Product Administration							
Study Product Administration #1	4 (100.0)	5 (100.0)	4 (100.0)	5 (100.0)	4 (100.0)	N/A	22 (100.0)

### Vaccine safety

No participants or household contacts became HIV infected during the study, and no participants or household contacts tested positive for vaccine induced seropositivity/seroreactivity (VISP/VISR) at the end of the study by HIV testing. There were no pregnancies reported during the study.

**Fig 3:**
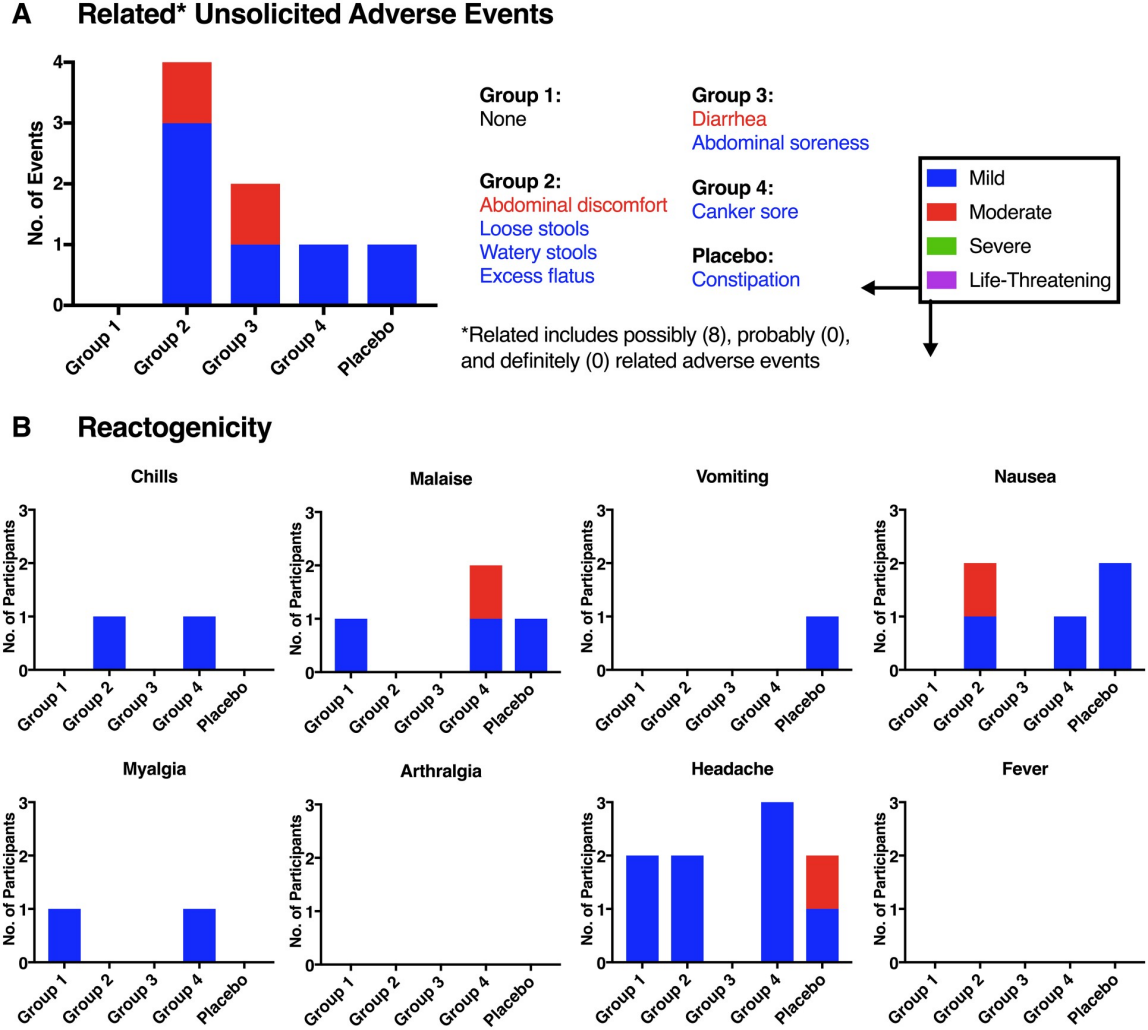
Vaccine safety. (A) Frequency of possibly, probably and definitely related (“Related”) unsolicited adverse events from the signing of the informed consent form until Study Day 112. Multiple adverse events could be reported per participant. (B) Number of participants who reported reactogenicity symptoms from the day of study product administration (Day 0) through Day 9. The National Institute of Allergy and Infectious Diseases Division of AIDS Toxicity Table was used to grade severity of adverse events and reactogenicity.

No serious adverse events were reported during the course of the study. A total of 32 non-serious unsolicited adverse events (Fig 3) were reported during the course of the study (30 were reported for participants, whereas two were reported among the household contacts); 20 of the 32 events (62.5%) were assessed as Grade 1 (mild) in severity, and 12/32 (37.5%) as Grade 2 (moderate); no adverse events were assessed as Grade 3 (severe) or higher in severity. Fifteen (14 participants, 1 household contact) of the 32 adverse events (46.9%) were classified under the MedDRA system organ class (SOC) for Gastrointestinal disorders, under which the most frequent preferred terms were constipation and diarrhea. Eight of the 30 (26.7%) participant reported events that were assessed as possibly related to the study product, whereas, 12/30 (40.0%) were considered to be probably not related to study product and 10/30 (33.3%) were considered to be not related to study product. A total of 6 participants (5 vaccine, 1 placebo) reported at least 1 unsolicited adverse event assessed as possibly, probably or definitely related to investigational product. There was no apparent relationship of adverse events to vaccine or dose among study participants. For adverse events reported for household contacts, assessment for relatedness to study product was not applicable.

### Vaccine-specific immune responses

**Fig 4:**
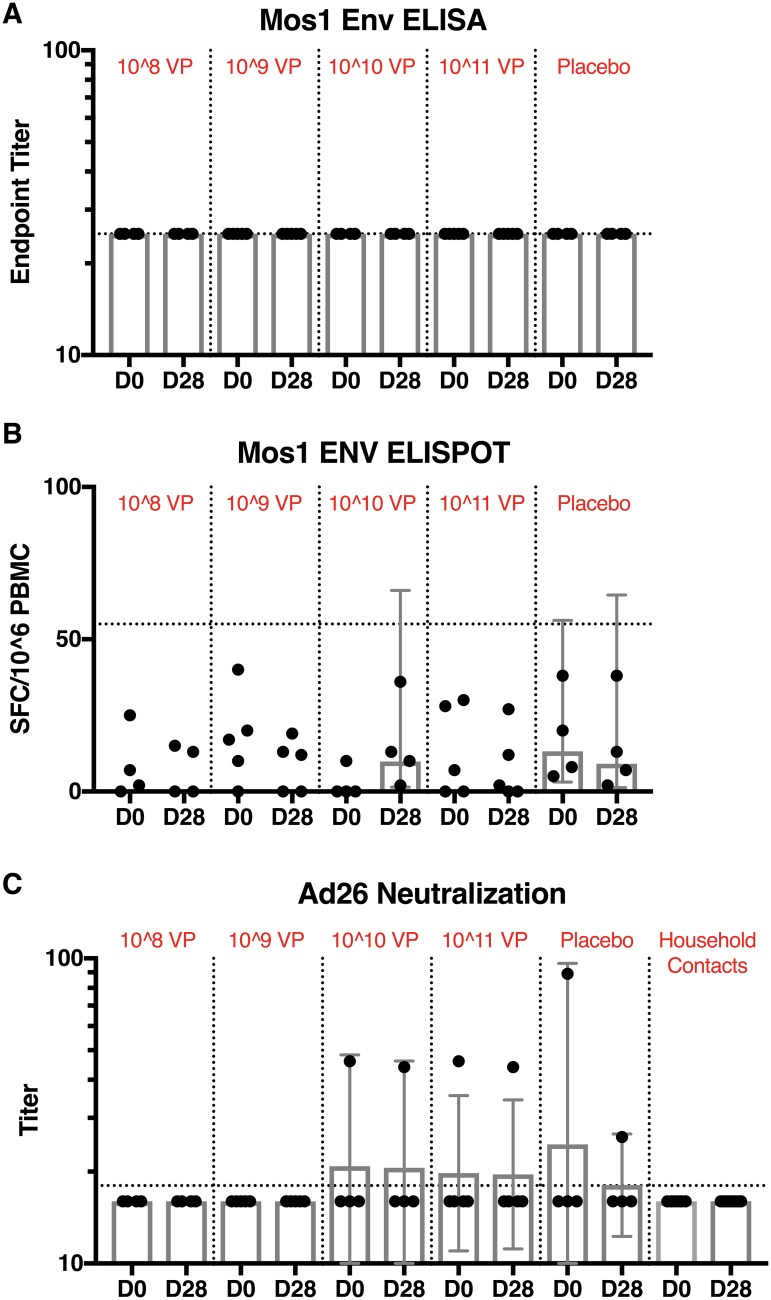
Vaccine-specific immune responses. (A) Mos1 Env-specific binding antibody responses, (B) Mos1 Env-specific T cell responses, and (C) Ad26 vector-specific neutralizing antibody responses by dose group. Individual responses from participant by day and dose group are shown. Dots show individual titers at a given time point. Summaries of responses are presented with geometric mean titers (GMTs), all with associated 95% confidence intervals (CIs). Dotted lines indicate threshold for positivity.

Analyses of participant serum samples at both Day 0 and Day 28 were below the assay limit of detection and presented undetectable end point binding antibody titers that fell below the pre-defined cutoff criteria of an absorbance >2-fold over mean background values and absolute optical density (O.D.) > 0.2 (Fig 4). HIV-1 Env-specific ELISPOT results were undetectable at day 0 and day 28 following vaccination. Vector-specific immunity was also not detected following immunization.

### Viral shedding

**Fig 5:**
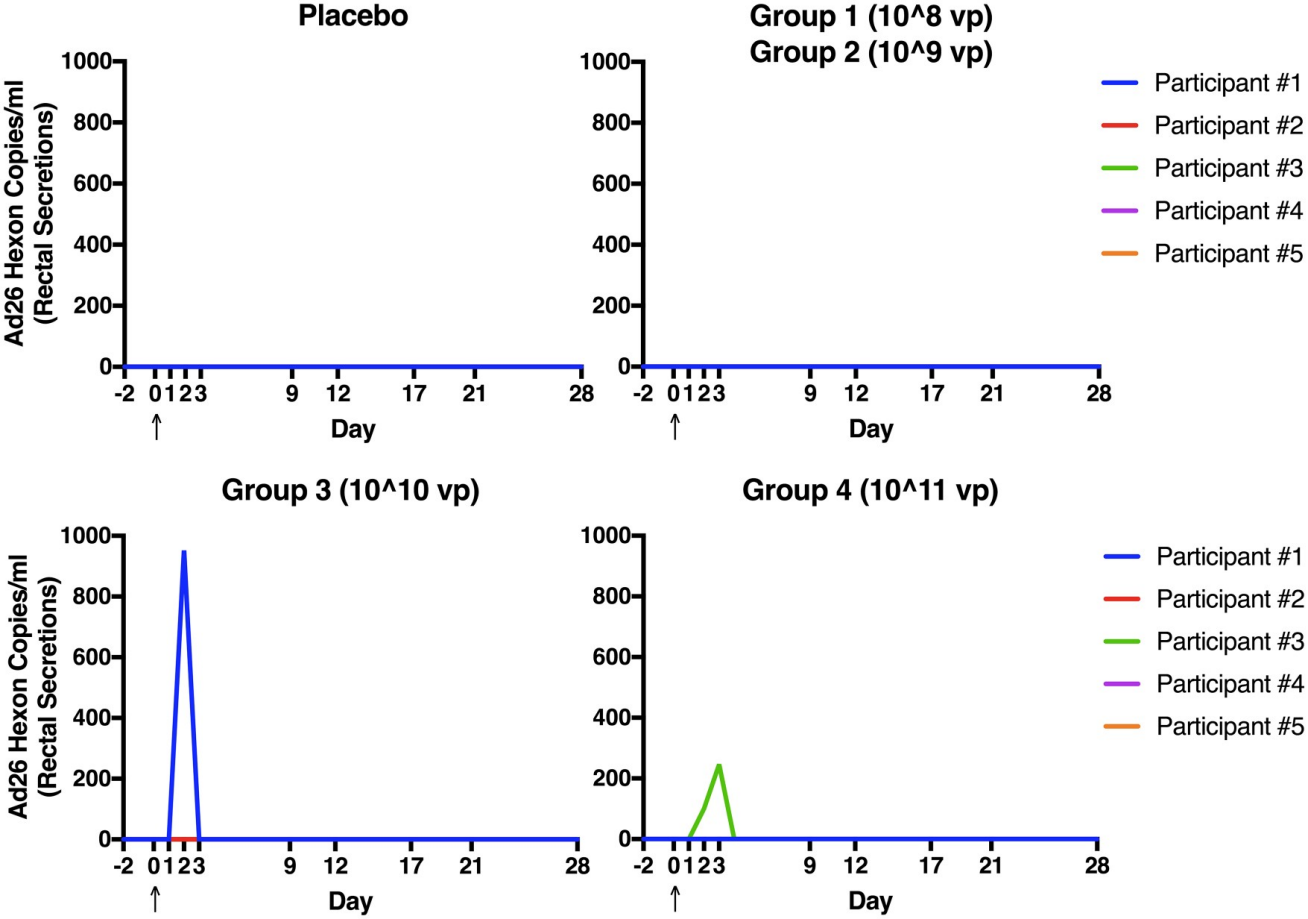
Viral shedding. Number of adenovirus serotype 26 (Ad26) hexon copies/ml detected by real-time (rt)PCR in rectal secretions collected on Day -2, 1, 2, 3, 4, 5, 6, 7, 8, 9, 12, 17, 21, and 28 for all groups. Arrow indicates day of study product administration. All samples with detectable Ad26 by PCR were negative by adenovirus culture.

As shown in Fig 5, no adenovirus 26 hexon (Ad26) was detected in rectal secretions from participants who received either placebo or the two lowest doses of vaccine (10^8 or 10^9 VP). Ad26 was detected by PCR in rectal secretions from 1 participant on day 3 following receipt of 10^10 VP of vaccine and in 1 participant on days 3 and 4 following receipt of 10^11 VP of vaccine. Ad26 was also detectable in plasma by PCR in one participant on D7 following receipt of 10^9 VP of vaccine (323 copies/ml). No Ad26 was detected in oral secretions of any vaccine recipients; 1 placebo recipient had detectable Ad26 in oral secretions on D5 (105 copies/ml). All samples with detectable Ad26 by PCR were negative by adenovirus culture and transgene PCR.

### Household contacts

Eleven household contacts were monitored. No HIV-1-specific antibodies were detected in household contacts at baseline or at day 28 of the study. There were also no symptoms reported by household contacts to suggest secondary transmission.

## Discussion

Replication-competent viral vectors are under study as HIV vaccine candidates because they may better mimic natural infection than replication-incompetent vectors, thereby leading to more comprehensive immune responses in humans. Such live vectors may have further enhanced immunogenicity if delivered orally or by other mucosal routes. We recently developed a replication-competent adenovirus serotype 26 vector to express HIV-1 antigens (rcAd26) and demonstrated that the vector exhibited a 4.4-log reduction in replicative capacity compared to wild type Ad26 in human cell lines. We also previously demonstrated that rcAd26 was immunogenic when delivered parenterally in mice and rhesus macaques[3], even though rcAd26 is actually replication-incompetent in animals. Here we report the results of a first-in-human, randomized controlled trial to test the safety, immunogenicity and mucosal shedding of this vaccine given orally in healthy HIV-1-uninfected adults.

In this study, 22 participants were isolated in an inpatient facility and administered varying doses of rcAd26.MOS1.HIV-Env (N = 18) or placebo (N = 4), given as oral capsules. Eleven (11) household contacts were monitored. The rcAd26 vaccine was well tolerated up to 10^11^ viral particles, with only mild to moderate adverse events reported by a handful of participants. Symptoms were predominantly gastrointestinal in nature, such as abdominal discomfort and diarrhea, and were not dose related. No serious adverse events were observed.

The rcAd26 vaccine given orally in humans was not immunogenic in this study. No HIV-specific or vector-specific humoral or cellular immune responses were detected at any dose. Low levels of vaccine were detected by PCR in rectal secretions from two participants at the highest doses within 3 days of vaccination, but no infectious vaccine was grown in culture. There was no evidence of secondary transmission of vaccine virus following the isolation period. No household contacts developed vaccine-induced HIV-1 seropositivity or vaccine-associated illness throughout the study.

The most likely explanation for the poor immunogenicity in humans of the live, oral rcAd26 HIV vaccine is that the replication competent vector was too attenuated to be given orally. We originally chose Ad26 for development as a live vector because wild type Ad26 infects the human gut, is transmitted via the oral-fecal route, and is infrequently associated with any clinical symptoms, and we hypothesized that replication of the vaccine in the gut might lead to enhanced mucosal HIV-specific immunity. In addition, replication-incompetent Ad26 vectors expressing HIV Env inserts were previously shown to be safe and immunogenic when given intramuscularly in humans in phase 1 studies[7–10]. We also knew that oral Ad4 and Ad7 vaccines had been given safely to prevent Ad4 and Ad7 respiratory illness in >10 million individuals [11–13]. Nevertheless, the final constructed rcAd26 vaccine was profoundly attenuated: the deletion of E3 and E4 and the insertion of the HIV-1 Env transgene resulted in a significant reduction in replicative capacity compared with that of wild-type Ad26 in the duodenal cell line HuTu80[3]. We did previously confirm *in vitro* that infectious rcAd26 survived the lyophilization and encapsulation process. But, it is unknown how much live virus was ultimately delivered to the duodenum in a real-world gastric environment. The rcAd26 vaccine was therefore handicapped by both high attenuation and, very likely, low inoculum. These are the probable reasons that our trial demonstrated little evidence that the vaccine was able to establish ongoing replication in the human gut *in vivo*.

Our results are consistent with a previous trial of an oral, live adenovirus serotype 4 vector vaccine for influenza (Ad4-H5-Vtn), which also showed minimal immunogenicity after a single dose[14]. In this trial, Ad4-H5-Vtn did not show significantly higher immune responses than placebo until it was boosted with intramuscular inactivated H5N1 vaccine, showing that oral, live adenovirus vectors might be effective tools to prime immune responses. This suggests that rcAd26 may have a role in combination with intramuscular HIV Env boosting, an approach not tested in this study. Oral, live vaccines may also require repetitive dosing over a short interval to ensure vaccine take. This was demonstrated formally in early studies of the licensed oral, live Salmonella typhi vaccine Ty21a, which is given as four doses on alternate days[15,16]. Such a dosing schema might have been more successful for rcAd26 as well.

Future studies of replication-competent vaccine vectors should consider carefully the degree of attenuation, the dosing schema and the route of administration when designing human studies. It is an open question as to whether rcAd26 would have been safe and immunogenic if given intramuscularly, or if a less attenuated vaccine would have had better luck when given orally. Upcoming studies with replication competent CMV and VSV and other adenovirus serotypes may help define the parameters of how to safely test these novel concepts [1].

## Conclusions

A live, oral HIV-1 vaccine (rcAd26.MOS1.HIV-Env) was well tolerated up to 10^11 VP in healthy, HIV-1-uninfected adults. There was no evidence of shedding of infectious virus or secondary vaccine transmission following the isolation period. The single dose of the vaccine was found to be poorly immunogenic, likely because the vector was too attenuated for oral administration. These data suggest the use of less attenuated viral vectors in future studies of live, oral HIV-1 vaccines.

## Supporting information

S1 Protocol R001 clinical trial protocol. This document is the complete clinical trial protocol.
(PDF)

S1 Checklist CONSORT checklist. (DOC)
