# Supplementary material for: First-in-human randomized controlled trial of an oral, replicating adenovirus 26 vector vaccine for HIV-1
Source: PLoS One. 2018 Nov 14;13(11):e0205139. doi: 10.1371/journal.pone.0205139 (PMC6235250; doi:10.1371/journal.pone.0205139)
Supplement: S1 Protocol — This document is the complete clinical trial protocol. (PDF) [file pone.0205139.s001.pdf]

**Protocol Title:**	A phase 1 clinical trial of the safety and immunogenicity of an oral, replicating adenovirus 26 vector vaccine for HIV-1 (rcAd26.MOS1.HIV-Env) in healthy HIV-1-uninfected adults
**Protocol Number:**	rcAd001/IAVI R001
**Phase:**	Phase 1
**IND Number:**	16246
**Sponsor:**	International AIDS Vaccine Initiative (IAVI)<br>125 Broad Street, 9th Floor<br>New York, New York 10004<br>USA
**Sponsor Status:**	Not for-Profit Organization
**Collaborating Institutions:**	Beth Israel Deaconess Medical Center<br>Boston, MA
**Funder:**	Bill and Melinda Gates Foundation<br>Seattle, WA
**Date of Protocol Version:**	01 Oct 2014<br>1.0
**Date of Amendment: Version:**	19 Feb 2015<br>2.0
**Date of Amendment: Version:**	02 Apr 2015<br>3.0

THE CONFIDENTIAL INFORMATION IN THIS DOCUMENT IS PROVIDED TO YOU AS AN INVESTIGATOR, POTENTIAL INVESTIGATOR, OR CONSULTANT, FOR REVIEW BY YOU, YOUR STAFF, AND APPLICABLE INSTITUTIONAL REVIEW BOARDS (IRBS) AND/OR INDEPENDENT ETHICS COMMITTEES (IECS). IT IS UNDERSTOOD THAT THE INFORMATION WILL NOT BE DISCLOSED TO OTHERS, EXCEPT TO THE EXTENT NECESSARY TO OBTAIN ETHICAL AND REGULATORY APPROVAL FROM THE RESPECTIVE COMMITTEE'S AGENCIES AND INFORMED CONSENT FROM THOSE PERSONS TO WHOM THE INVESTIGATIONAL PRODUCT MAY BE ADMINISTERED.

TABLE OF CONTENTS

1 OVERVIEW....8
2 BACKGROUND AND RATIONALE....11
2.1 Introduction....11
2.2 Rationale for developing the replicating adenovirus 26 vector (rcAd26)....11
2.2.1. The difference between Ad5 and Ad26 vectors....12
2.3 Rationale for developing the mosaic HIV-1 Env immunogen (MOS1.HIV-Env)....13
3 PRECLINICAL EXPERIENCE....15
3.1 Preclinical experience with rcAd26....15
3.1.1 *In vitro* characterization of rcAd26....15
3.1.2 *In vivo* characterization of rcAd26....16
3.1.3. Preclinical experience with non-replicating Ad26 vectors expressing Env....17
3.2 Preclinical experience with MOS1.HIV-Env....17
4 CLINICAL EXPERIENCE....19
4.1 Clinical experience with natural adenovirus 26 infection....19
4.2 Clinical experience with oral, live adenovirus vaccines....20
4.3 Clinical experience with non-replicating Ad26 vectors expressing Env....20
5 OBJECTIVES....22
6 STUDY DESIGN....23
6.1 Safety Assessment....23
6.2 Viral Shedding Assessment....24
6.3 Immunogenicity Assessment....24
6.4 Monitoring....25
6.5 Household Contacts....25
6.6 Randomization Procedures and Enrollment....25
6.7 Method of Blinding and Unblinding....26
7 STUDY POPULATION....27
7.1 Volunteer Inclusion Criteria....27
7.2 Volunteer Exclusion Criteria:....28
7.3 Household Contact Inclusion Criteria....30
7.4 Household Contact Exclusion Criteria....31
8 STUDY PRODUCTS....32
8.1 Study Products....32
8.1.1 rcAd26.MOS1.HIV-Env....32

8.1.2 Placebo 32
8.2 Handling of Study Treatments..32
8.2.1 Packaging and Labeling 32
8.2.2 Shipment and Storage..32
8.2.3 Dose preparation and administration 32
9 CONDUCT OF THE STUDY 33
9.1 Informed Consent 33
9.1.1 Screening Consent Form..33
9.1.2 Protocol-Specific Consent Form 33
9.1.3 Assessment of Understanding...33
9.2 Clinical and Laboratory Evaluations – Volunteers 34
9.3 Unscheduled Visits 34
9.4 Unblinding Visit 34
9.5 Criteria for dose advancement and advancement within a single group 34
9.6 HIV Counseling and Testing 34
9.6.1 Distinguishing intercurrent HIV infection from vaccine-induced seroreactivity 34
9.7 Prior and Concomitant Treatment 35
9.8 Study discontinuation...35
9.8.1 Early discontinuation or withdrawal of volunteers 35
9.8.2 Premature termination of the study...36
9.8.3 Study pausing rules..36
9.8.4 Admission of back up volunteers 38
9.9 Laboratory evaluations...38
9.10 Potential risks and benefits 38
9.10.1 Risks related to vaccines..38
9.10.2 Blood draws 39
9.10.3 Unknown risks...39
9.10.4 Potential benefits..39
10 SAFETY ASSESSMENTS..40
10.1 Adverse events...40
10.1.1 Definitions 40
10.1.2 Surveillance, reporting, and documentation of adverse events...............41
10.1.3 SAE Reporting...43
10.2 Reporting requirements to the local IRB...43
10.3 Causality assessment of study vaccine to adverse events 43
10.3.1 Severity of adverse events 44

10.3.2 Follow-up of ongoing adverse events and assessment of outcome........45
10.3.3 Treatment of adverse events........46
10.4 Handling of pregnancy cases........46
10.5 Physical examination........47
10.6 Routine safety laboratory........47
10.7 Vital signs........47
10.8 Monitoring for HIV infection........47
10.8.1 Management of volunteers who become HIV-infected during the study .48
10.9 Protocol Safety Review Team and Safety Monitoring Committee........48
11 IMMUNOGENICITY AND VIRAL SHEDDING ASSAYS........49
11.1 HIV-associated immunogenicity........49
11.2 Anti-vector immunity........49
11.3 DNA/RNA Assays........50
11.4 Additional Immunogenicity Assays........50
11.5 Viral shedding assays........50
12 DATA EVALUATION AND STATISTICS........51
12.1 Analysis populations and data sets........51
12.1.1 Safety population........51
12.1.2 Immunogenicity population........51
12.2 Endpoints........51
12.3 Sample size consideration........53
13 ETHICAL AND LEGAL REQUIREMENTS........53
13.1 General requirements........53
13.2 Institutional Review Board/Ethics Committee........53
13.3 Regulatory authorities........53
13.4 Volunteer information and informed consent........54
13.5 Indemnity........54
13.6 Data access and protection........54
13.7 Future use and storage and blood samples........55
13.8 Reimbursement........55
14 QUALITY CONTROL AND QUALITY ASSURANCE........56
14.1 Monitoring........56
14.2 Audit and inspections........56
14.3 Data quality assurance........56
15 DOCUMENTATION, ARCHIVING, AND PUBLICATIONS........57
15.1 Documentation........57

15.2 Archiving 57
15.3 Clinical Study Reports and Publications 57
16 REFERENCES 58
17 PROTOCOL SIGNATURE PAGE 66
18 APPENDICES 67
18.1 Toxicity Tables 67
18.2 HIV Risk Assessment 88
18.3 Measures to Prevent Transmission of Vaccine to Staff 89
18.4 Key Study Roles 89
18.5 Schedule Of Procedures - Volunteers 92
18.6 Schedule of Procedures – Household Contacts 93
18.7 Blood Volumes (mL) - Volunteers 94
18.8 Blood Volumes (mL) – Household Contacts 95

List of Abbreviations

AE	Adverse event
ADCC	Antibody-dependent cell-mediated cytotoxicity
ADCVI	Antibody-dependent cell-mediated virus inhibition
AIDS	Acquired immunodeficiency syndrome
ALT	Alanine aminotranferase
AST	Aspartate aminotransferase
BIDMC	Beth Israel Deaconess Medical Center
CAR	Coxsackie Adenovirus Receptor
CBC	Complete blood count
$CD4^+$	A functional subclass of T cells, helper T lymphocytes (Th), that are necessary for augmentation and coordination of innate and adaptive effector responses, humoral and cellular
$CD8^+$	Cytotoxic T-cells that destroy host cells that have become infected by viruses or other intracellular pathogens
CDC	Center for Disease Control
CFR	Code of Federal Regulations
CMV	Cytomegalovirus
CPE	Cytopathic Effect
CRF	Case report form
CTL	Cytotoxic T lymphocyte
CVVR	Center for Virology and Vaccine Research
DNA	Deoxyribonucleic acid
DMC	Data Management Center
ELISA	Enzyme linked immunosorbent assay
ELISPOT	Enzyme-linked immunospot
Env	Envelope
FDA	US Food and Drug Administration
GALT	Gut-Associated Lymphoid Tissue
GCP	Good Clinical Practices
GLP	Good Laboratory Practices
GMS	Global Medical Safety
HIV-1	Human immunodeficiency virus, type 1
HVTN	HIV Vaccine Trials Network
IB	Investigator’s Brochure
ICH	International Conference on Harmonization
ICF	Informed Consent Form
IND	Investigational New Drug
IP	Investigational Product
IRB	Institutional Review Board
mL	Mililiters
MVA	Modified Vaccinia Ankara virus
NHP	Non-human primate
PBMC	Peripheral blood mononuclear cells
PCR	Polymerase chain reaction
PI	Principal investigator
PO	Per Oral
PSRT	Protocol Safety Review Team
RBC	Red blood cell count

rcAd26	Replication competent human adenovirus type 26 vector
rtPCR	Real-time PCR
SAE	Serious adverse event
SIV	Simian Immunodeficiency Virus
SMC	Safety Monitoring Committee
SST	Serum separating tubes
SUSAR	Suspected unexpected serious adverse reaction
$TCID_{50}$	50% Tissue Culture Infective Dose
VISR	Vaccine-induced seroreactivity
VP	Viral particles
WBC	White blood cell count

1 OVERVIEW

Title

A phase 1 clinical trial of the safety and immunogenicity of an oral, replicating adenovirus 26 vector vaccine for HIV-1 (rcAd26.MOS1.HIV-Env) in healthy HIV-1-uninfected adults

Primary Objective

- To evaluate the safety and tolerability of a single oral administration of rcAd26.MOS1.HIV-Env vaccine administered in a dosage escalation of 10^8, 10^9, 10^{10}, and 10^{11} viral particles.

Secondary Objectives

- To evaluate the shedding of rcAd26.MOS1.HIV-Env in rectal and oropharyngeal secretions.
- To evaluate humoral and cellular immune responses elicited by four increasing dosages of rcAd26.MOS1.HIV-Env HIV-1 vaccine.

Exploratory Objectives

- To perform additional host response assays including measurement of Ad26-specific antibodies, HIV-1-specific antibody-dependent cell-mediated cytotoxicity (ADCC) and cell-mediated viral inhibition (ADCVI) assays, epitope mapping of T lymphocytes by ELISPOT, gene expression profiling, and analysis of mucosal humoral immune responses.
- To investigate the possibility of transmission of rcAd26.MOS1.HIV-Env to volunteers' household contacts after volunteers leave isolation unit.

Study Products and Routes of Administration

- **rcAd26.MOS1.HIV-Env**: Replication competent adenovirus serotype 26 vector vaccine is a recombinant product composed of a replication competent adenovirus serotype 26 vector that encodes a mosaic Env glycoprotein. The dose will range from 10^8 to 10^{11} viral particles (vp), delivered as enteric-coated capsules.
- **Placebo**: The placebo product is a sucrose-containing capsule.

Table 1: Study Schema

Study Group*	rcAd26.MOS1.HIV-Env / Placebo	Dosage (Day 0)	Delivery	Setting
1	5 / 1	10^{8} vp / Placebo	PO	Isolation**
2	5 / 1	10^{9} vp / Placebo	PO	Isolation
3	5 / 1	10^{10} vp / Placebo	PO	Isolation
4	5 / 1	10^{11} vp / Placebo	PO	Isolation
Total Number	20 / 4			

*Starting with Group 1, each group will be sequentially enrolled following PSRT and SMC reviews of safety data for a period of at least 9 days post vaccination.

**Volunteers will be housed in an isolation unit from day -2 through day 9.

Volunteers

Healthy, HIV-1-uninfected volunteers aged 18-50 years:

20 vaccinees
4 placebo recipients
24 total volunteers

Design

Single center, isolation unit, dose escalation, randomized, controlled, double-blind phase 1 trial

Duration per volunteer

12 months active follow-up per volunteer

Estimated total study duration

14 months (includes screening and active follow-up)

Investigational New Drug (IND) Sponsor

International AIDS Vaccine Initiative (IAVI), New York, New York, USA

Vaccine Manufacturer

PaxVax Corporation, San Diego, California, USA
Batavia Bioservices, Leiden, The Netherlands

Data Management Center (DMC)

The EMMES Corporation, Rockville, Maryland, USA

Endpoint Assays Laboratory

Center for Virology and Vaccine Research (CVVR), Beth Israel Deaconess Medical Center (BIDMC), Boston, Massachusetts, USA

Study Site

University of Rochester Medical Center, Rochester, New York, USA

Safety Monitoring

Protocol Safety Review Team (PSRT)
Safety Monitoring Committee (SMC)

2 BACKGROUND AND RATIONALE

2.1 Introduction

Prevention of HIV-1 remains a public health priority, even in an era of widespread antiretroviral treatment. In the United States, the Centers for Disease Control and Prevention (CDC) estimates that of all HIV-1-positive US residents, approximately one fifth are unaware of their status, and this small proportion is responsible for transmitting half the new infections each year [1]. Worldwide, a much larger percentage of HIV-positive individuals remain undiagnosed, and access to appropriate therapy is more difficult. According to the World Health Organization, 6,300 people are newly infected with HIV-1 every day, and more than 95% of newly HIV-1-infected people live in low and middle income countries that have limited access to antiviral medication [2]. It is an urgent global health priority to find a safe and potent HIV-1 vaccine that would prevent HIV-1 infection or blunt its initial impact prior to diagnosis, i.e., diminishing the destruction of the gut CD4+ T cell pool [3] or risk of HIV-1 transmission [4]. Such a vaccine, to have a global impact, should be simple to administer, long-acting, and effective against all types of HIV-1 transmission, including the most frequent—exposure at the mucosal surface.

Experience to date with protein ‘subunit’ vaccines such as gp120 and replication incompetent vectors such as adenovirus 5 (Ad5) has been disappointing, with failure to show benefit in several efficacy trials [5-8]. A combined regimen of the replication incompetent canarypox vector administered with gp120 showed transient protection that was considered inadequate for development of these precise candidate vaccines, though research to improve the approach is ongoing [9]. In this protocol we describe our plan to test a new, orally-administered vaccine based on a replication competent adenovirus 26 (Ad26) vector that expresses a ‘mosaic’ HIV-1 envelope protein engineered to provide maximal coverage of global HIV-1 sequence diversity.

2.2 Rationale for developing the replicating adenovirus 26 vector (rcAd26)

The development of an HIV-1 vaccine remains a critical goal in the effort to decrease HIV-1 incidence and end the worldwide epidemic. To date, only one vaccine trial, RV144, has demonstrated efficacy in preventing HIV-1 acquisition [9]. RV144 evaluated an ALVAC canarypox prime and an AIDSVAX B/E recombinant gp120 boost in low-risk individuals in Thailand, with an observed efficacy of 31.2% in reducing HIV-1 infection rates. This modest level of protection was demonstrated in the absence of robust CD8+ T cell or neutralizing antibodies responses to primary HIV-1 isolates. Follow-up analyses of RV144 showed that antibodies to variable loops 1 and 2 (V1V2) regions of HIV-1 Env were associated with a reduced risk of HIV-1 acquisition [10,11], and studies in nonhuman primates have also shown that Env-specific antibodies were associated with partial protection against viral challenge [12,13]. Optimally, however, both a robust CD4+/CD8+ T cell response and a potent humoral response with multiple effector functions would be induced by a vaccine.

Despite the modest protection observed in RV144, it remains unclear how to increase the potency and durability of HIV-1-specific immunity. One strategy to improve these immune responses is to use live, replicating vectors to express HIV-1 antigens [14]. Live-attenuated viral vaccines have been shown to be effective in both humans and animals [14] and include some of the most widely-used licensed vaccines in the world, e.g.,

vaccines for measles-mumps-rubella (MMR-II), yellow fever (YF-VAX), varicella (Varivax and Zostavax), influenza (FluMist), and rotavirus (Rotarix and RotaTeq) [15]. Attenuated simian immunodeficiency virus (SIV, with *nef* deletion) has long been known to elicit protection against challenge with pathogenic SIV in rhesus monkeys [16-19], but live-attenuated HIV-1 has not been pursued as a vaccine concept due to safety concerns [20]. Nevertheless, recent studies have shown that immunization with a replicating CMV vector expressing SIV antigens can lead to immune clearance of highly pathogenic SIV infection in rhesus monkeys [21]. This data suggests that live vectors expressing HIV-1 antigens might approximate a live, attenuated HIV-1 vaccine while also having a better safety profile.

The protective efficacy of live viral vaccines is likely because these vaccines mimic natural intracellular infection, thereby eliciting more comprehensive innate, cellular, and humoral immune responses to vaccine antigens. In addition, many live vaccines are delivered via mucosal surfaces – such as intra-nasally or orally – and may therefore lead to enhanced mucosal immunity. These features are relevant to HIV-1 vaccine design, as in most cases HIV-1 acquisition will have to be blocked at mucosal portals of viral entry and HIV-1 targets mucosal CD4+ T cells within gut-associated lymphoid tissue (GALT) [22]. It is likely that protective mucosal immunity will require antibody responses as well as host CD8+ T cells that can limit replication of HIV-1 and kill HIV-1-infected cells, thus dampening or eliminating a local, mucosal focus of infection during the early days of infection [23]. To elicit such protective mucosal immunity, it is desirable to have a vaccine vector that might mimic natural HIV-1 infection in gut mucosa.

2.2.1. The difference between Ad5 and Ad26 vectors

All three of the HIV-1 vaccine efficacy studies used adenovirus type 5 (Ad5)-based vectors (HVTN 502, HVTN 503, HVTN 505) and showed no efficacy against HIV-1 infection. In the first two studies increased HIV-1 infection was observed in vaccinees as compared with placebo volunteers. The mechanism for this possible increase in HIV-1 acquisition risk remains unclear, but a leading hypothesis involves activation of vector-specific CD4+ T lymphocytes at mucosal surfaces following Ad5 vaccination, potentially resulting in increased targets for HIV-1 infection.

The rationale to continue clinical development of Ad26 vector-based vaccines for HIV-1 is based on data showing that: (1) Ad26 is biologically substantially different than Ad5; (2) Ad26-based vaccines afford superior protective efficacy compared with Ad5-based vaccines against stringent SIVmac251 challenges in rhesus monkeys; and (3) Ad26 did not increase the number or activation status of total or vector-specific CD4+ T lymphocytes at mucosal surfaces in humans following vaccination in a randomized, double-blinded, placebo-controlled clinical trial (IPCAVD-003) [24].

The data that Ad5 and Ad26 are biologically different are as follows: (1) Ad5 seroprevalence is nearly universal with high titers in the developing world, whereas Ad26 seroprevalence is lower with substantially lower titers in the developing world [25,26]. (2) Ad5 utilizes CAR as its primary cellular receptor (Ad26 utilizes CD46), exhibits primarily liver tropism *in vivo* (Ad26 exhibits no liver tropism), and Ad5 immune complexes are more stimulatory to human dendritic cells than are Ad26 immune complexes [27-29]. (3) Ad5 and Ad26 exhibit profoundly different innate immune profiles following vaccination of human volunteers, as demonstrated by differential gene expression profiles on Days 1, 3, and 7 after immunization (Dan Zak and Alan Aderem, Seattle BioMed, unpublished).

(4) Ad5 and Ad26 vectors exhibit profoundly different adaptive immune phenotypes. For example, in mice, Ad5 vectors elicited high magnitude but dysfunctional and exhausted T cells, characterized by low levels of CD127, CD62L, and IFN-gamma, and high levels of PD-1, whereas Ad26 vectors elicited polyfunctional T cells characterized by high levels of CD127, CD62L, and IFN-gamma, and low levels of PD-1 [30,31]. Moreover, Ad26-induced T cells expanded more robustly than did Ad5-induced T cells upon re-exposure to antigen and afforded superior protective efficacy in murine challenge models.

In addition, there is evidence that Ad26 has improved protective efficacy compared to Ad5 in rhesus monkeys. Consistent with the results of the HVTN 505 clinical trial, DNA/Ad5 vaccines afforded no protective efficacy against stringent, repetitive, intrarectal challenges with the neutralization-resistant virus SIVmac251 in rhesus monkeys [32]. DNA/Ad5 vaccines afforded partial protective efficacy against less stringent, neutralization-sensitive SIVsmE660 challenges, indicating that the low stringency SIVsmE660 challenge model was not predictive of the clinical results observed in HVTN 505. Ad5 only vaccines also did not afford protection against SIVmac239 [33], consistent with the results of the Step study. In contrast, Ad26/MVA and Ad26/Ad35 vaccine regimens afforded substantial protective efficacy against repetitive, intrarectal challenges with the neutralization-resistant virus SIVmac251 in rhesus monkeys [12]. Thus, Ad26/MVA vaccines afforded partial protection (76-83% reduction in the per-exposure risk of infection) in the stringent SIVmac251 challenge model in which DNA/Ad5 failed.

Finally, there was no activation of mucosal total or vector-specific CD4+ T lymphocytes following Ad26 vaccinations in humans, based on a randomized, double-blinded, placebo-controlled clinical trial (IPCAVD-003) [24]. In this study, 24 volunteers were enrolled (16 Ad26 seronegative, 8 Ad26 seropositive) and randomly allocated to receive Ad26.ENVA.01 vaccine or placebo in a 3:1 ratio. Blood samples, colorectal biopsies, and colorectal Weck-cel sponges were collected prior to immunization and at Weeks 2 and 24 following vaccination. HIV-1 Env-specific humoral and cellular immune responses were observed in both peripheral blood and colorectal mucosa following Ad26 vaccination. However, Ad26 vector-specific CD4+ T-lymphocyte responses were substantially lower in colorectal mucosa than in peripheral blood and also did not appear to increase following vaccination. In addition, no increased inflammation was observed in colorectal mucosa following vaccination. Normal histology was observed in 71/71 biopsies, and there were no increases in CD4, CD8, CD3, or CD25 cells, or Ki67 or HLA-DR activation.

2.3 Rationale for developing the mosaic HIV-1 Env immunogen (MOS1.HIV-Env)

One of the additional challenges in HIV-1 vaccine development is the tremendous diversity of HIV-1 strains worldwide [34-42]. Because of the ability of HIV-1 to evade immune responses through mutational escape, there is constant viral evolution within populations and individuals. There are 13 distinct subtypes and sub-subtypes of HIV-1, as well as hundreds of circulating recombinant forms, and between-subtype variation can be as large as 35% [37,38,43]. HIV-1 also diversifies extensively within each host. Korber et al. have demonstrated that the variability of HIV-1 within one host is comparable to the global variation of circulating influenza A during a single season [44]. This extreme genetic diversity makes it difficult to design an HIV-1 vaccine that can elicit broadly protective immunity.

The mosaic HIV-1 Env immunogen (MOS1.HIV-Env) was developed to address this challenge of HIV-1 sequence diversity. Mosaic immunogens are bioinformatically engineered to optimize coverage of global HIV-1 sequence diversity through analysis of a database of global HIV-1 sequences at Los Alamos National Laboratory [45-52]. Studies of the T cell responses induced by previous vaccines using natural HIV-1 sequences have shown that there is relatively limited breadth of recognition of HIV-1 epitopes, compared to the wide variety of circulating strains [53]. In contrast, studies have shown that mosaic sequences increase the breadth and depth of immune responses compared with consensus or natural sequences in monkeys [48,49]. It has also recently been shown that adenovirus and poxvirus vectors expressing mosaic Gag, Pol, and Env sequences afforded a significant reduction in per-exposure risk of SHIV acquisition in monkeys [13]. Moreover, mosaic Env antigens induced non-inferior binding and neutralizing antibodies compared with these other antigens [51].

3 PRECLINICAL EXPERIENCE

3.1 Preclinical experience with rcAd26

The vaccine candidate proposed for this Phase 1 clinical study is a replication competent human adenovirus serotype 26 (rcAd26), used as a vaccine vector to deliver a MOS1.HIV-Env transgene. The vaccine is referred to as rcAd26.MOS1.HIV-Env and is an Ad26 virus with the E3 and part of the E4 genes (orf 1-4) deleted to allow the MOS1.HIV-Env transgene to be inserted. Adenoviruses are generally highly species specific and the human Ad26 virus is not known to replicate in any laboratory animal. However, the *in vitro* replicative fitness of rcAd26.MOS1.HIV-Env growth can be assessed in human cell lines. In addition, *in vivo* immunogenicity studies can be performed in mice and rhesus monkeys, with the understanding that the vector will be replication incompetent in these species. These assessments were performed at CVVR as part of the preclinical evaluation of rcAd26.MOS1.HIV-Env and are summarized below.

3.1.1 *In vitro* characterization of rcAd26

The infectivity and growth kinetics of rcAd26 vectors were assessed in human cell lines to study how the deletion of E3/E4 and the insertion of the MOS1.HIV-Env transgene resulted in viral attenuation. Here, attenuation is defined by virus titer required to infect cells and the time to achieve maximum cytopathic effect (CPE). The cell lines used included A549 (epithelial lung carcinoma cells), HuTu 80 (duodenum adenocarcinoma cells), and Per55K (epithelial cells that complement the Ad E1 region). The rcAd26 vectors used in this study included the clinical candidate (rcAd26.MOS1.HIV-Env) and wild-type Ad26, as well as other versions of the vector that had either the E3 or E3/E4 region deleted, or were with or without the MOS1.HIV-Env transgene. Non-replicating versions of these vectors were also assessed.

The *in vitro* replicative capacity of all rcAd26 vectors was significantly reduced as compared to wild-type Ad26 in human epithelial and duodenal cells. Decreased *in vitro* replicative capacity occurred in a step-wise fashion with vectors containing only the E3 deletion having only a slight reduction, then becoming more reduced as both E3 and E4 were deleted. Replicative capacity was most reduced in vectors containing the HIV-1 Env transgene, with the clinical candidate rcAd26.MOS1.HIV-Env having the most pronounced decrease in replicative capacity, with approximately 100-fold more virus required to achieve the same virus growth compared to wild-type Ad26. As expected, the non-replicating Ad26 vectors did not replicate in either the A549 or HuTu 80 cell lines. As a control, the vector growth in E1-complementing PER.55K cells was evaluated. The data show that there was no significant difference in growth between the various Ad26 vectors as compared to wild-type when PER.55K cells were infected.

The *in vitro* infectivity of rcAd26 vectors was also compared to Ad4-H5-Vtn, an Ad4-based vector expressing influenza antigens which was recently found safe in a previous clinical trial [54]. The rcAd26.MOS1.HIV-Env vaccine had a significantly decreased replication capacity compared to Ad4-H5-Vtn in A549 and HuTu 80 cells. In these cell lines, wild-type Ad26, wild-type Ad4 (Ad4.WT), and Ad4-H5-Vtn all grew equally well with rcAd26.MOS1.HIV-Env having significantly decreased growth in comparison. As expected, all vectors grew well in PER.55K cells. Finally, the ability of rcAd26 to infect

rhesus monkey cells (MK2, kidney cell line) was assessed. No CPE was seen at any MOI out to 6 days post-infection when rcAd26 vectors were used to infect MK2 cells.

In summary, the rcAd26.MOS1.HIV-Env vaccine had at least a 100-fold less replicative capacity compared with wild-type Ad26. In addition, growth of rcAd26.MOS1.HIV-Env was significantly reduced *in vitro* as compared to Ad4-H5-Vtn, a vector shown to be safe in a previous clinical trial. Finally, no evidence of rcAd26 virus replication in terms of CPE was detected in the rhesus monkey cell line MK2.

3.1.2 *In vivo* characterization of rcAd26

The immunogenicity of rcAd26.MOS1.HIV-Env was first assessed in Balb/c mice. Four mice were immunized by intramuscular (IM) injection with 10^9 vp of vaccine, sacrificed at 28 days, and the splenocytes assessed by IFN-γ ELISPOT. Env-specific ELISPOT responses were detected in all mice. To assess if the immunogenicity of lyophilized rcAd26.MOS1.HIV-Env had been affected by lyophilization, Balb/c mice (n=4) were also immunized by IM injection with 10^8 or 10^9 vp of two different lots of reconstituted lyophilized rcAd26.MOS1.HIV-Env, as well as bulk drug substance. This study showed that lyophilized and reconstituted rcAd26.MOS1.HIV-Env remained infectious as well as immunogenic when compared to non-lyophilized rcAd26.MOS1.HIV-Env in terms of PFU, ELISPOT and ELISA assays. Finally, *in vivo* replication of replication-incompetent Ad26 was compared to that of replication-competent rcAd26 in mice by immunizing Balb/c mice (n=4) with 10^{10} vp IM injection or intra-nasally (IN). The HIV-1 specific antibody responses were similar for the replication and non-replication product given either by IM or IN administration in these studies. The T-cell responses were much greater when the vectors were given by IM administration. There was no evidence of replication of vectors in mice. Though no safety data was collected in these non-GLP studies, all mice in all groups appeared healthy and well and no adverse effects in mood or physical appearance were observed.

Next, the immunogenicity of rcAd26 was assessed in nonhuman primates (NHP). Rhesus monkeys (Macaca Mulatta) (N=3/group) were immunized by IM injection with 10^{11} vp with replicating E1(+) and non-replicating E1(-) Ad26-SIVGag vectors at weeks 0 and 24. IFNγ-ELISPOT was performed at weeks 0, 2, 24, 26 and 32 to determine immune response. RT-PCR on serum was done at weeks -1, 1, 4, 6, 8, 10, 12 and 16 to look at virus replication. Both replication-competent and replication-incompetent Ad26.SIVGag were immunogenic in non-human primates. There was no evidence by RT-PCR that the replication-competent vector replicates in NHP.

In the non-GLP NHP immunogenicity study depicted above, various non-GLP safety assessments were made in the three NHPs that received the rcAd26-SIVGag vector at week 0 and week 24 by IM injection. Note that these studies were designed as small immunogenicity studies and were not specifically designed as safety studies. The safety observations were as follows: (1) No clinical adverse events (AEs) were observed in the three NHPs following immunization with 10^{11} vp rcAd26-SIVGag. Non-GLP clinical observations included daily assessments of behavior, feeding patterns, and local and/or systemic reactogenicity as well as weekly assessments of body weights by senior veterinarians and veterinary technicians at New England Primate Research Center (NEPRC), Southborough, MA, which is affiliated with Harvard Medical School. (2) No clear changes in hematology parameters were observed in the three monkeys following immunization with 10^{11} vp rcAd26-SIVGag, with the exception of mild decreases in

hematocrit as a result of the maximum allowable blood draws that were performed to facilitate the immunogenicity studies. Non-GLP blood hematology included assessments of white blood cell counts, hematocrit, cellular differentials, and T cell subpopulations. (3) No clear changes in serum chemistries were observed in the three monkeys following immunization with 10^{11} vp rcAd26-SIVgag at week 0 and week 24. Non-GLP serum chemistries included assessments of electrolytes, renal function, hepatic function, and other chemistries.

3.1.3. Preclinical experience with non-replicating Ad26 vectors expressing Env

Formal toxicology studies of rcAd26.MOS1.HIV-Env were not performed as the rcAd26 vector does not replicate in animals. The non-replicating Ad26 vector (E1 deleted), however, has been assessed in a GLP single-dose biodistribution study and a GLP repeated-dose toxicity study in New Zealand white rabbits performed at Avanza Laboratories (formerly Bridge Global Pharmaceutical Services / GeneLogic), Gaithersburg, MD. The biodistribution study (Bridge GPS Study No.1645-06074) demonstrated that IM administration of Ad26.ENVA.01 was well tolerated in male and female New Zealand white rabbits. Ad26.ENVA.01 remained primarily in the injection site muscle and distributed only to the iliac lymph nodes and spleen. Over the time course of the 91-day study, Ad26.ENVA.01 was shown to clear from inoculated animals as was indicated by the decline in percentage of animals with positive tissues (from a majority or all of the animals to only 1-2 animals) and the copy numbers detected (declining by more than 1-2 logs). The repeated-dose toxicity study also found that 4 repeat IM administrations of Ad26.ENVA.01 at doses up to 10^{11} vp were well tolerated in male and female New Zealand white rabbits and did not result in any toxicologically significant effects. Changes in some clinical pathology, hematology and immunology parameters as well as histopathological findings at the injection site were consistent with an inflammatory response to the vaccine administration.

3.2 Preclinical experience with MOS1.HIV-Env

The mosaic HIV-1 Env immunogen (MOS1.HIV-Env) has been evaluated extensively in mice and nonhuman primates [13,47,51,52,55,56]. In addition to the studies described above, Barouch et al. assessed cellular immune responses to mosaic HIV-1 vaccines in rhesus monkeys immunized with a single dose of non-replicating Ad26 expressing mosaic, consensus, or natural HIV-1 Gag, Pol, and Env sequences [51]. The breadth and depth of T cell responses (measures of T cell epitope diversity) were greatest for the mosaic antigens compared to consensus and natural sequences. Mosaic Env immunogens also elicited non-inferior antibody responses as compared with the consensus or natural sequence Env antigens following heterologous boost. Similar results were seen in a mosaic vaccine study in NHP by Santra et al. [52]. In a challenge study, rhesus monkeys were immunized with Ad26, Ad35, and MVA vectors expressing mosaic HIV-1 Env, Gag, and Pol in different prime/boost combinations, and were subsequently challenged with 6 low-dose, intrarectal challenges with SHIV-SF162P3 [13]. Both the Ad/MVA and Ad/Ad vaccines afforded partial protection against acquisition of SHIVSF162P3 infection [13], corresponding to an 87-90% reduction of per exposure acquisition risk, although the majority of the vaccinees became infected by the end of the challenge series. The vaccinees also exhibited a modest but significant 0.64 log reduction of setpoint viral loads, which was measured 84 days after infection, as compared with the controls.

Recently, an NHP challenge study addressed correlates of protection by interrogating virus sequences and vaccine-induced immune responses following immunization with mosaic immunogens [56]. Animals were vaccinated with DNA and Ad5 expressing either mosaic Gag or mosaic heterologous Env, or heterologous Env based on a natural SIVmac239 sequence. The authors found that Env-elicited immune responses were necessary and sufficient to provide protection from acquisition from intrarectal SIVsmE660 with a strong sieving effect of Env immunization, selecting for minor variants in the challenge swarm. There was no association between protection from infection and protection from pathogenesis, suggesting that humoral responses that effectively block acquisition are not necessarily correlated with cellular responses that control pathogenesis. The Env-induced CTL also suppressed acute viremia better than Gag CTL resulting in reduced transmitted-founder viruses in breakthrough infections [56].

As noted above, formal toxicology studies of rcAd26.MOS1.HIV-Env were not performed as the rcAd26 vector does not replicate in animals. The MOS1.HIV-Env insert, however, has been assessed in a repeated dose GLP toxicity and local tolerance study of the Modified Vaccinia Ankara (MVA) vector expressing the same Mosaic Env sequence in New Zealand white rabbits. Vaccine-related changes in body temperatures, clinical pathology parameters, and pathology data were generally resolved and/or were trending toward recovery, were not deemed to be adverse, and were considered a normal immunologic/inflammatory response to the vaccine administration. None of these effects during the study for animals were adverse to the health of the animal.

4 CLINICAL EXPERIENCE

4.1 Clinical experience with natural adenovirus 26 infection

Adenovirus serotype 26 (Ad26) is a non-enveloped DNA virus first discovered in the stool of healthy children in Washington, D. C. in 1961 [57]. Like other Group D adenoviruses, Ad26 infects the human gut and is likely transmitted via the oral-fecal route [58]. Epidemiologic studies show that Ad26 is extremely rare even among gut adenoviruses, and is infrequently associated with any clinical symptoms. Early data showed that Ad26 accounted for <1% of all adenovirus isolates from 18,096 children hospitalized in the United States in 1969 and was not associated with clinical illness [59]. Since that time, Ad26 has not been identified in any follow up survey of adenovirus isolate serotypes in the developed world including among 178 healthy families in New York [60], 215 healthy families in Seattle [61], 137 hospitalized children with diarrhea in London [62], 30 hospitalized children in New York [63], and 90 healthy adults/children in Toronto [64]. In keeping with this data, only 8-11% of individuals from the United States were found to have detectable Ad26-specific neutralizing antibodies in a recent survey [65].

Ad26 is also rare in the developing world, where adenoviruses account for only 6% of viral gastroenteritis in children [66-68]. For example, Ad26 was identified in <2% of adenovirus isolates in stool from 104 Kenyan children in 2010 [69]. Other surveys of adenovirus isolate serotypes outside the U.S. have failed to identify Ad26 altogether, including among 1,301 fecal samples and 7,921 nasopharyngeal samples from patients with diarrhea and/or respiratory illness in Thailand from 2009-2012 [70], 2,233 outpatient and hospitalized children with diarrhea in China from 2011-2012 [71], 2,234 children with respiratory illness in China from 2009-2012 [72], 106 hospitalized children with diarrhea in China in 2010 [73,74], 113 hospitalized children with diarrhea in Korea from 2004-2006 [75], and 9 children with severe acute respiratory disease in Paraguay in 2010 [76]. Seroprevalence surveys confirm that Ad26-specific neutralizing antibodies are more common in the developing world compared to the United States, but titers are low [65,77]. Among individuals at low risk for HIV in sub-Saharan Africa, 43% had detectable Ad26-specific antibodies, but >90% of these antibodies were within the lowest titer tier [65]. These data suggest that most Ad26 infection in the developing world produces subclinical disease, and is likely due to increased opportunities for oral-fecal transmission.

As described above, there is no evidence from observational studies that enteric infection with Ad26 causes clinical symptoms in healthy adults. Experimental data from an Ad26 challenge study in 1963 also suggests that enteric Ad26 infection does not produce symptoms [78]. In this study, 8 volunteers were inoculated with $10^{4.5}$ $TCID_{50}$ Ad26 intranasally. Six volunteers developed an enteric infection, excreting Ad26 in the stool for multiple days, without any abdominal pain, fever, or diarrhea. An additional 13 volunteers had Ad26 swabbed on the lower conjunctival sac and developed conjunctivitis. Eleven of these volunteers developed Ad26 enteric infection, excreting Ad26 in stool, but reported no GI symptoms.

While Ad26 infection is not found naturally among healthy adults, Ad26 can be isolated more easily from immunocompromised populations. This was first noted in 1988, when a single Ad26 was identified among 67 adenovirus isolates recovered from patients with

acquired immunodeficiency syndrome (AIDS) [79]. Surveillance sampling also recovered Ad26 from the stool of 2 patients with AIDS in England [80], and 2 HIV-1-infected patients in Lima, Peru [81]. In addition, Ad26 was identified in the central nervous system (CNS) of a patient with medulloblastoma following irradiation and ventriculo-peritoneal shunt placement; this is the only report in the literature of Ad26 isolated outside the gut [82]. Interestingly, Ad26 has not been identified among adenovirus isolates in the transplant population, including in 22 children following liver transplant [83], and in 19 children and 35 adults following bone marrow transplant [84,85].

4.2 Clinical experience with oral, live adenovirus vaccines

The above data suggest that Ad26 infection produces subclinical enteric disease, and would be an appropriate vector to deliver vaccine antigens to the gut mucosa. In addition, oral, live attenuated adenovirus vaccines have a proven track record of safety and efficacy for the prevention of Ad4 and Ad7 respiratory illness [86-88]. These vaccines have been administered safely to more than 10 million people over the span of nearly sixty years [88] to prevent mini-epidemics of debilitating and even life-threatening adenovirus disease in military recruits [87]. Oral, live recombinant Ad4 (Ad4-H5-Vtn) has also recently been shown safe and immunogenic as a vector to express influenza antigens in humans [54]. In addition, these oral adenovirus vaccine studies have provided data on vaccine shedding in stool among volunteers. In the clinical trial of Ad4-H5-Vtn, shedding was first detected, and detected in the most volunteers, at day 7 following immunization, and after day 14 was not detected in any volunteer [54]. Similar patterns of shedding were also observed in studies of oral Ad1, Ad2, Ad4, Ad5, Ad7, and Ad21[89-97].

4.3 Clinical experience with non-replicating Ad26 vectors expressing Env

Non-replicating Ad26 vectors expressing the Env antigen have been tested in humans as part of three Phase 1 studies under the IPCAVD (Integrated Preclinical/Clinical AIDS Vaccine Development) NIH-sponsored program. In addition to providing safety data about non-replicating Ad26 vectors, these studies also provide supporting safety data about the use of Env inserts. These inserts are similar to the MOS1.HIV-Env insert (69% sequence overlap); MOS1.HIV-Env itself has not previously been tested in humans.

In IPCAVD001, a non-replicating Ad26 vector expressing EnvA.01 (Ad26.ENVA.01) was evaluated in a dose escalation study in 60 healthy, HIV-1 uninfected adults [98,99]. Ad26.ENVA.01 vaccine was generally safe and well-tolerated at all doses tested (10^9 through 10^{11} vp, given intramuscularly) and up to 3 vaccinations. There were no vaccine-related SAEs. At the 10^{11} dose, reactogenicity (fatigue, myalgias, and chills) was seen 12 hours after the 1st vaccination and resolved spontaneously. These symptoms were not seen after the 2nd vaccination. Consistent Env antibody titers and Env T cell responses were seen following the prime which increased after the 6 month boost and were durable at 1 year in vaccinated volunteers.

In a follow up study IPCAVD003, Ad26.ENVA.01 was tested in 24 healthy, HIV-1 uninfected adults to obtain a detailed evaluate of safety, mucosal immunogenicity, and innate immune responses following intramuscular immunization [24]. The majority of volunteers reported mild or no tenderness at the vaccination site. Nine out of 18 vaccine recipients experienced at least 1 systemic reactogenicity symptom that was moderate or severe. In the 5 vaccine recipients who experienced at least 1 systemic severe reactogenicity symptom, these symptoms largely occurred between day 0 to 1 and were

typically a combination of headache, chills, joint pain, myalgia, and malaise/fatigue. There were no study product related SAEs. All volunteers developed detectable Ad26-specific Nabs, and 16 of 18 volunteers had detectable peripheral EnvA IgG ELISA titers. Mucosal EnvA-specific IgG ELISA responses were detected in 11 of 18 volunteers. EnvA-specific CD8+ and CD4+ T cell responses were also detected in the majority of volunteers. There were stable levels of Ki67+ cellular activation in total and vector specific CD4+ T lymphocytes following Ad26.ENVA.01 vaccination. There was no increase in CCR5 expression in total or vector specific CD4+ T lymphocytes in PBMCs or colorectal mucosa following vaccination.

Finally, in IPCAVD004, Ad26.ENVA.01 and Ad35Env vaccines were evaluated in various prime/boost combinations in 218 healthy HIV-1 uninfected adults in the US and in South and East Africa. The majority of both local and systemic reactogenicity reactions were graded as mild or moderate and resolved spontaneously. There were three unrelated SAEs. There was induction of Ad26 Nabs and EnvA ELISA titers in the majority of vaccinees, as well as detectable T cell responses.

5 OBJECTIVES

Primary Objective

- To evaluate the safety and tolerability of a single oral administration of rcAd26.MOS1.HIV-Env HIV-1 vaccine administered in a dosage escalation of 10^8, 10^9, 10^{10}, and 10^{11} viral particles.

Secondary Objectives

- To evaluate the shedding of rcAd26.MOS1.HIV-Env in rectal and oropharyngeal secretions.
- To evaluate humoral and cellular immune responses elicited by four increasing dosages of rcAd26.MOS1.HIV-Env HIV-1 vaccine.

Exploratory Objectives

- To perform additional host response assays including measurement of Ad26-specific antibodies, HIV-1-specific antibody-dependent cell-mediated cytotoxicity (ADCC) and cell-mediated viral inhibition (ADCVI) assays, epitope mapping of T lymphocytes by ELISPOT, gene expression profiling, and analysis of mucosal humoral immune responses.
- To investigate the possibility of transmission of rcAd26.MOS1.HIV-Env to volunteers' household contacts after volunteers leave isolation unit.

6 STUDY DESIGN

This is a phase 1 trial of an oral, replication competent adenovirus 26-vectored HIV-1 vaccine. Because the vaccine is replication competent, volunteers will be housed in an isolation unit from day -2 through day 9 in order to limit any transmission of the viral vector if shedding occurs. The trial will be conducted under a placebo controlled, double-blind, randomized allocation of study product. This design is intended to reduce the likelihood of observer bias, provide control for confounding variables of intercurrent illness, and aid in the interpretation of laboratory data. The study will also be conducted using a dose-escalation design, wherein the group receiving the lowest dose (group 1) will be monitored for at least 9 days following vaccination before planned PSRT and SMC safety reviews prior to enrollment in the next group. Shedding data will not be available for inclusion in this safety review (see Section 6.2). There are four groups in the study and blinding will be maintained to vaccine or placebo within each group as noted in the table below.

Table 1: Study Schema

Study Group*	rcAd26.MOS1.HIV-Env / Placebo	Dosage (Day 0)	Delivery	Setting
1	5 / 1	10^8 vp / Placebo	PO	Isolation**
2	5 / 1	10^9 vp / Placebo	PO	Isolation
3	5 / 1	10^{10} vp / Placebo	PO	Isolation
4	5 / 1	10^{11} vp / Placebo	PO	Isolation
Total Number	20 / 4	*Starting with Group 1, each group will be sequentially enrolled following PSRT and SMC reviews of safety data for a period of at least 9 days post vaccination.<br><br>**Volunteers will be housed in an isolation unit from day -2 through day 9.		

It is anticipated that enrollment will be completed within a 6-month period, and that volunteers will be actively followed for 12 months.

6.1 Safety Assessment

On Days 0-9 post vaccination of the isolation portion of the study, a study clinician (MD, NP, and/or nurse) will assess the volunteers with a directed medical history and abbreviated physical examination (once daily, noting any medication use), review the volunteer’s reactogenicity diary card (once daily), and check vital signs (twice daily). Any unsolicited adverse events observed or reported at any time of day will also be noted.

After the completion of the isolation portion of the study, adverse events will be assessed at follow up visits, as detailed in the Schedule of Procedures (Section 18.5). Hematology and blood chemistries will also be assessed as detailed in the Schedule of Procedures.

6.2 Viral Shedding Assessment

In order to reduce the possibility of transmission, volunteers will be housed in an isolation facility for 9 days following receipt of the vaccine (Days -2 to 9). Based on prior studies with other oral replicating adenovirus vectors, it is believed that maximum viral shedding will occur approximately 7 days following administration of the rcAd26.MOS1.HIV-Env vaccine. Given the 100-fold reduced replicative capacity of the vaccine compared to wild type virus and the limits of how long volunteers can reasonably be expected to stay in an isolation unit, the 9 day period was deemed appropriate. Within the facility, volunteers will reside alone in a hospital-style room with its own shower and toilet; volunteers will not be allowed to leave his or her room following immunization for the duration of the isolation period and can receive no visitors. Clinical staff will use contact precautions (gloves and disposable gown) when in physical contact with volunteers and/or in the volunteers' room; no other staff will enter the volunteers' room. Upon discharge from the isolation unit, instructions will be provided to each volunteer regarding methods to limit the possibility of transmission. Rectal and throat swabs will be assessed for rcAd26.MOS1.HIV-Env by PCR as outlined in Schedule of Procedures (Section 18.5); if a rectal or throat swab is positive by PCR, then adenovirus culture will also be performed. Plasma adenovirus PCR will be performed on day 7 but results will not be available prior to discharge from the isolation unit; if plasma adenovirus PCR is positive by PCR, then adenovirus culture will also be performed.

Real-time (rt)PCR analysis will be performed for both the Ad26 vector and the Env insert to allow for quantitation of vector and insert and to evaluate the stability of the vaccine. If the PCR is positive, adenovirus cultures will also be performed to evaluate for the presence of infectious virus and to allow eventual correlation of PCR and culture results; PCR will be performed on positive cultures to confirm that the virus is rcAd26.MOS1.HIV-Env.

All shedding samples will be frozen, and all PCR and cultures will be performed at CVVR. Investigators and study clinicians will be blinded to adenovirus PCR and culture results, but a single individual on the study team will be unblinded to gain access to the results from day 28. If a volunteer's adenovirus PCR remains positive at day 28, weekly adenovirus PCR will be performed until the PCR is negative.

6.3 Immunogenicity Assessment

Blood samples will be obtained at various time points after immunization (Section 18.5). These will be assayed for the magnitude of antibody binding to HIV-1 Env as quantified by enzyme-linked immunosorbant assay (ELISA), the magnitude of neutralizing antibodies against HIV-1 as quantified by virus neutralization assay, and the magnitude of HIV-1-specific T lymphocyte responses as quantified by interferon-gamma (IFN-y) enzyme-linked immunospot assay (ELISPOT). Additional studies may include measurement of Ad26-specific antibodies, ADCC and ADCVI, epitope mapping, and mucosal immune responses. Laboratory studies will be conducted in the labs of Drs.

Dolin, Seaman, and Barouch at the CVVR. Additionally, rectal mucosal secretions will be collected with rectal wicks for evaluation of potential mucosal immunity to HIV-1 Env and Ad26 (this procedure will be optional for volunteers). Studies may include measurement of Ad26 and HIV-1-specific antibodies by ELISA, and antibody epitope mapping to Env peptides by linear peptide microarray.

6.4 Monitoring

The Safety Monitoring Committee (SMC) and Protocol Safety Review Team (PSRT) will review the safety data through Day 9 post vaccination for all volunteers in each dosage group. In addition, the SMC will oversee the study as outlined in Section 10.9, and will advise the PSRT as needed. *Ad-hoc* safety review may be specifically requested by the Sponsor, PaxVax, Batavia, the Principal Investigators, Ethics Committees, Regulatory Authorities, PSRT or SMC.

6.5 Household Contacts

Study volunteers will be required to live alone or have no more than 2 adult household contacts, as detailed in the inclusion criteria. A household contact is defined as an individual who shares the same residence as the volunteer. Household contacts will provide informed consent and will be enrolled in the study as a household contact. After a volunteer has been discharged from the isolation facility, the volunteer's household contacts will be contacted on a weekly basis for a period of 2 weeks to assess for the presence of illness that may be related to transmission of rcAd26.MOS1.HIV-Env. If such illness is suspected, the household contact will be seen in clinic to further assess if Ad26 was the cause of illness. This may include blood tests as well as rectal or throat swabs for PCR and culture, depending on the type of illness present. Household contacts will be consented and continue to be followed for a period of 8 months, as detailed in the Schedule of Procedures for household contacts in Section 18.6.

6.6 Randomization Procedures and Enrollment

Volunteers will be enrolled in the study after ascertainment by one of the study investigators that all of the inclusion and none of the exclusion criteria have been met. Once confirmation of eligibility for the trial has been performed, the volunteer will be randomized. Randomized treatment assignments will be generated by the Data Management Center (DMC). After successful randomization, an allocation number is provided. The DMC will create the randomization table and the sponsor will monitor the implementation of this process. The allocation number is referenced against a confidential list provided to an unblinded on-site pharmacist to determine the assignment/treatment allocation.

The 6 volunteers in each group will be randomized in a ratio of 5 vaccinees to 1 placebo recipient. The study preparation will be provided to the study nurses for oral administration under a coded, masked identification. For the 10^8 and 10^{10} doses, volunteers will receive a single capsule, while the doses at 10^9 and 10^{11} will consist of 10 capsules of 10^8 or 10^{10}, respectively. For each group, placebo recipients will receive the same number of capsules as those receiving the active treatment. For the 10^8 and 10^9 doses, the placebo and vaccine preparations will be indistinguishable in the preparation provided for volunteers. For the 10^{10} and 10^{11} doses, the placebo capsules will be

smaller than the vaccine capsules (size 3 vs. size 1), but will otherwise be indistinguishable.

6.7 Method of Blinding and Unblinding

The volunteers, clinical staff, investigator, and sponsor personnel will be blinded to treatment allocation throughout the study. A single member of the study team will have unblinded access to Ad26 shedding cultures results from day 28 after vaccination. The pharmacist with primary responsibility for vaccine dispensing will not be blinded to the treatment and will maintain the randomization code and complete assignments of volunteers according to the randomization allocation. For the 10^{10} and 10^{11} doses, a single member of the clinical staff with primary responsibility for vaccine administration to volunteers will not be blinded to the treatment, given that the vaccine and placebo capsules are different sizes; this staff member will not participate in clinical management or data analysis. For these doses, the volunteers will remain blinded as they will be isolated from one another. Routine unblinding of treatment allocations may occur only after all volunteers have had their last study visit and the database is locked.

7 STUDY POPULATION

The study population will include healthy men or women aged 18-50 years old who are able and willing to provide written informed consent. Because of the possibility of transmission with the use of a replication competent vaccine, volunteers will reside alone or with no more than 2 adults, who must consent to participate in the study as a Household Contact. Volunteers will be enrolled in the study once eligibility criteria are met.

7.1 Volunteer Inclusion Criteria

1. Age 18-50 years old.

2. Ability and willingness to provide informed consent.

3. Assessment of understanding: completion of a questionnaire prior to first screening procedure; verbally demonstrate understanding of all questionnaire items answered incorrectly.

4. Available for the duration of the trial, including willingness to be kept in an isolation facility for Days -2 to 9.

5. From Day 10 through 4 months following vaccination must reside alone or with no more than 2 adults (age 18-60) and no minors under age 18 who are willing to consent to participate in the study as a Household Contact. A Household Contact is defined as an individual who shares the same residence as the volunteer.

6. Good general health as shown by medical history, physical exam, and screening laboratory tests.

7. The following laboratory parameters:
   a. Hematology
      - Hemoglobin ≥11.0 g/dL for women; ≥12.5 g/dL for men
      - Absolute Neutrophil Count (ANC): ≥1000/mm^3
      - Absolute Lymphocyte Count (ALC): ≥500/mm^3
      - Platelets: 125,000 to 550,000/mm^3
   b. Chemistry
      - Creatinine: <1.1 x upper limit of normal (ULN)
      - AST: <1.25 x ULN
      - ALT: <1.25 x ULN
   c. Normal urinalysis
      - Negative urine glucose
      - Negative or trace urine protein
      - Negative or trace urine hemoglobin (if trace hemoglobin is present on dipstick, a microscopic urinalysis within institutional range)

8. Willing to undergo HIV testing and receive HIV test results, and willing to receive risk reduction counseling

9. All female volunteers must be willing to undergo urine beta human chorionic gonadotropin pregnancy tests at time points indicated in the Schedule of Procedures (Section 18.4) and must test negative prior to vaccination

10. All sexually active males (unless anatomically sterile) must be willing to use an effective method of contraception (such as consistent condom use) from the day of vaccination until at least 4 months after vaccination

11. If a woman of child-bearing potential, committed to use an effective method of contraception when sexually active with men for 4 months following the receipt of vaccine, including:
    a. Condoms (male or female) with or without spermicide
    b. Diaphragm or cervical cap with spermicide
    c. Intrauterine device
    d. Hormonal contraception
    e. Successful vasectomy in the male partner (considered successful if a woman reports that a male partner has [1] documentation of azoospermia by microscopy, or [2] a vasectomy more than 2 years ago with no resultant pregnancy despite sexual activity post-vasectomy)
    f. Not be of reproductive potential, such as having undergone hysterectomy, bilateral oophorectomy, or tubal ligation

12. Willing to forgo donations of blood or any other tissues during the study and, for those who test HIV positive due to trial vaccination (vaccine-induced HIV seroreactivity), until the anti-HIV antibody titers become undetectable

13. Low risk for HIV infection (see Section 18.2) and willing to maintain low-risk behavior for the trial duration

7.2 Volunteer Exclusion Criteria:

1. Confirmed HIV-1 or HIV-2 infection; confirmed HCV, HBV, or syphilis infection

2. Current or planned participation in another clinical trial of an experimental agent during the study period

3. Pregnant or lactating

4. Significant acute or chronic disease, including inflammatory bowel disease or other chronic gastrointestinal disease, immunodeficiency syndrome, or condition requiring immunosuppressant medications (the use of topical or inhaled steroids is permitted)

5. Use of anticancer, antituberculosis or other medications considered significant by the investigator within the previous 6 months

6. Receipt of live-attenuated vaccine within the previous 60 days or planned receipt within 60 days after vaccination with Investigational Product (within 14 days for live attenuated influenza vaccine [LAIV]); or receipt of other vaccine (e.g., influenza, pneumococcal), allergy treatment with antigen injections or tuberculin

skin test within the previous 14 days or planned receipt within 14 days after vaccination with Investigational Product

7. Receipt of blood transfusion or blood-derived products within the previous 3 months

8. Receipt of HIV vaccine(s) in a prior HIV vaccine trial. For volunteers who have received control/placebo in an HIV vaccine trial, the Medical Monitor will determine eligibility on a case-by-case basis.

9. Previous severe local or systemic reactions to vaccination

10. History of splenectomy

11. History of seizure in the last 3 years (volunteers with a history of seizures who have neither required medications nor had a seizure for 3 years are not excluded)

12. Known autoimmune disease

13. Asthma other than mild, well-controlled asthma. Exclude a volunteer who:
    - Generally uses a bronchodilator (beta 2 agonist) weekly, or
    - In the past year has (any of the following):
        - Had > 1 exacerbation of symptoms treated with oral steroids
        - Routinely used moderate to high dose inhaled corticosteroids (e.g., more than the equivalent of 250 mcg fluticasone; 400 mcg budesonide; 500 mcg beclomethasone; or 1000 mcg triamcinolone/flunisolide, as a daily dose) or theophylline
        - Needed emergency care, urgent care, hospitalization, or intubation for asthma
    - Prophylactic bronchodilator use prior to exercise is not exclusionary

14. Diabetes mellitus type 1 or type 2, including cases controlled with diet alone. (Not excluded: history of isolated gestational diabetes.)

15. Thyroidectomy, or thyroid disease requiring medication during the last 12 months

16. Angioedema within the last 3 years if episodes are considered serious or have required medication within the last 2 years

17. Hypertension:
    - If a person has been diagnosed with hypertension during screening or previously, exclude for hypertension that is not well controlled. Well-controlled hypertension is defined as blood pressure consistently ≤ 140 mm Hg systolic and ≤ 90 mm Hg diastolic, with or without medication, with only isolated, brief instances of higher readings, which must be ≤ 150 mm
    - If a person has NOT been diagnosed with hypertension during screening or previously, exclude for systolic blood pressure ≥ 150 mm Hg at enrollment or diastolic blood pressure ≥ 90 mm Hg at enrollment

18. Body mass index (BMI) ≥ 40; or BMI ≥ 35 with 2 or more of the following: systolic blood pressure > 140 mm Hg, diastolic blood pressure > 90 mm Hg, current smoker, known hyperlipidemia

19. Bleeding disorder diagnosed by a doctor (e.g. factor deficiency, coagulopathy, or platelet disorder requiring special precautions)

20. Malignancy (Not excluded: a volunteer with a surgical excision and subsequent observation period that in the investigator's estimation has a reasonable assurance of sustained cure or is unlikely to recur during the study period)

21. Psychiatric condition that compromises safety of the volunteer or precludes compliance with the protocol, specifically excluding persons with psychoses within the past 3 years, ongoing risk for suicide, or history of suicide attempt or gesture within the past 3 years

22. All healthcare workers with direct patient contact

23. Childcare worker who has direct contact with children

24. Individuals employed as food handlers or otherwise engaged in the preparation or delivery of food outside of their place of residence

25. A urine toxicology test positive for opioids, cocaine, or amphetamines.

7.3 Household Contact Inclusion Criteria

Household contacts must be deemed eligible according to the following criteria, before the volunteer is vaccinated:

1. Able to understand and give written consent, and complete an assessment of understanding

2. Healthy (based on medical history and physical examination) men or women aged 18-60.

3. Low risk for HIV infection (see Section 18.2) and willing to maintain low-risk behavior for 4 months following the volunteer's vaccination

4. Willing to undergo HIV testing and receive HIV test results and as well as risk-reduction counseling

5. Willing to forgo blood or tissue donation for 4 months after the volunteer's vaccination; for those who test HIV positive due to trial vaccination (vaccine-induced HIV seropositivity), until the anti-HIV antibody titers become undetectable

6. If a woman of child-bearing potential, committed to use an effective method of contraception when sexually active with men for 4 months following the volunteer's receipt of vaccine, including:
    a. Condoms (male or female) with or without spermicide

b. Diaphragm or cervical cap with spermicide
c. Intrauterine device
d. Hormonal contraception
e. Successful vasectomy in the male partner (considered successful if a woman reports that a male partner has [1] documentation of azoospermia by microscopy, or [2] a vasectomy more than 2 years ago with no resultant pregnancy despite sexual activity post-vasectomy)
f. Not be of reproductive potential, such as having undergone hysterectomy, bilateral oophorectomy, or tubal ligation

7. All sexually active males (unless anatomically sterile) must be willing to use an effective method of contraception (such as consistent condom use) from the conclusion of the isolation period until at least 4 months after vaccination

7.4 Household Contact Exclusion Criteria

1. Significant acute or chronic disease, including inflammatory bowel disease or other chronic gastrointestinal disease, immunodeficiency syndrome, or condition requiring immunosuppressant medications (the use of topical or inhaled steroids is permitted)

2. Asthma other than mild to moderate, well-controlled asthma. Exclude a volunteer who:
    - Generally uses a bronchodilator (beta 2 agonist) daily, or
    - In the past year has (any of the following):
        - Had > 1 exacerbation of symptoms treated with oral steroids
        - Routinely used high dose inhaled corticosteroids (e.g., more than the equivalent of 500 mcg fluticasone; 1200 mcg budesonide; 500 mcg beclomethasone; or 1000 mcg triamcinolone/flunisolide, as a daily dose) or theophylline
        - Needed hospitalization, or intubation for asthma
    - Prophylactic bronchodilator use prior to exercise is not exclusionary

3. Pregnant or lactating

4. HIV infection

5. Individuals employed as a healthcare worker with direct patient contact, childcare worker who has direct contact with children <5 years old, or food handler (or an individual who is otherwise engaged in the preparation or delivery of food outside of their place of residence)

8 STUDY PRODUCTS

8.1 Study Products

Detailed instructions for study products including preparation, storage and documentation are provided under separate cover in the Study Operations Manual. Additional information is also provided in the Investigator’s Brochure for the rcAd26.MOS1.HIV-Env product.

8.1.1 rcAd26.MOS1.HIV-Env

rcAd26.MOS1.HIV-Env vaccine is formulated as Acryl-EZE enteric-coated capsules for oral administration. The 10^8 and 10^9 doses will be administered in size 3 capsules. The 10^{10} and 10^{11} doses will be administered in larger size 1 capsules.

8.1.2 Placebo

The placebo is formulated as Acryl-EZE enteric-coated capsules for oral administration. Capsules will be size 3.

8.2 Handling of Study Treatments

8.2.1 Packaging and Labeling

rcAd26.MOS1.HIV-Env and placebo capsules will be provided in Nalgene 1 oz. size plastic amber colored polyethylene container with a polypropylene closure and labeled in accordance with Good Manufacturing Practice (GMP). Ten drug product capsules are packaged per container.

8.2.2 Shipment and Storage

rcAd26.MOS1.HIV-Env and placebo capsules should be stored frozen at -20 °C (± 10 °C). One rayon ball is used as packing inside the Nalgene bottles to prevent damage to the capsules during shipping and handling.

8.2.3 Dose preparation and administration

The unblinded pharmacist (or other unblinded staff member qualified to handle and dispense medication) will dispense rcAd26.MOS1.HIV-Env or placebo capsules in a single dose container labeled with the volunteer’s study ID and allocation number. Volunteers will be asked to swallow the capsule(s) with a glass of non-carbonated water.

9 CONDUCT OF THE STUDY

9.1 Informed Consent

The informed consent form documents that a volunteer (1) understands the potential risks, benefits, and alternatives to participation, and (2) is willing to participate in this study. Informed consent encompasses all written or verbal study information staff provide to the volunteer before and during the trial.

The informed consent process continues throughout the study. At each study encounter, staff should consider reviewing the procedures and requirements for that encounter and for the remaining encounters. Additionally, if any new information is learned that might affect the volunteer's decision to stay in the trial, this information will be shared with trial volunteers. If necessary, volunteers will be asked to sign a revised informed consent form.

Volunteers must sign a screening or protocol-specific consent before any procedures to determine eligibility are performed. All recruitment and prescreening materials will be approved by the Institutional Review Board.

9.1.1 Screening Consent Form

An IRB approved general vaccine screening protocol and consent may be used as part of the initial screening procedure for this trial. Results from this IRB approved general screening or prescreening may be used to determine eligibility in this protocol, provided the tests are conducted within the time period specified in the eligibility criteria.

9.1.2 Protocol-Specific Consent Form

The protocol-specific consent form describes the study products to be used and all aspects of protocol participation, including screening and enrollment procedures. The consent form is developed in accordance with local IRB requirements and the principles of informed consent as described in Title 45, Code of Federal Regulations (CFR) Part 46 and Title 21 CFR, Part 50, and in the International Conference on Harmonization (ICH) Guideline 4.8.10. It must be approved by all responsible ethical review bodies before any volunteers can be consented for the study.

9.1.3 Assessment of Understanding

Study staff should ensure that volunteers understand the study before screening them. This process involves reviewing the informed consent form with the volunteer, allowing time for the volunteer to reflect on the procedures and issues presented, and answering all questions completely. An Assessment of Understanding is used to document the volunteer's understanding of key concepts in this HIV vaccine trial. The volunteer must complete the Assessment of Understanding—with the assistance of staff, if necessary, in reading and understanding the questions and responses—before screening. Volunteers must verbalize understanding of all questions answered incorrectly. This process and the volunteer's understanding of the key concepts should be recorded in source documentation at the site.

9.2 Clinical and Laboratory Evaluations – Volunteers

Please see Sections 18.5 and 18.7 for a concise outline of study procedures. Screening should occur within 56 days of enrollment. Volunteers will be admitted to the isolation facility 2 days before the vaccine is administered in order to allow for any incubating illness to emerge prior to the administration of the vaccine. During the isolation period, volunteers will be seen twice daily. For visit windows, refer to the Schedule of Procedures (Section 18.5).

9.3 Unscheduled Visits

Unscheduled Visits/Contacts are visits/contact that are not described in the Schedule of Procedures (Section 18.5). Unscheduled visits may occur any time during the study:

- For administrative reasons, e.g., the volunteer or household contact may have questions for study staff or may need to re-schedule a follow-up visit.
- To obtain laboratory test results from a previous visit.
- For other reasons as requested by the volunteer or site investigator.

All unscheduled visits will be documented in the volunteer's study records on applicable source documents and entered into the Case Report Form (CRF).

9.4 Unblinding Visit

After all volunteers (from all 4 groups) have completed the active follow-up part of the study, and the database is locked, unblinding will occur. Volunteers will be informed as to whether or not they received study product or placebo and whether they have vaccine induced seroreactivity. For those with a false positive HIV serology at the end of the study, follow-up testing will be offered. The unblinding visit may be performed in person or by phone.

9.5 Criteria for dose advancement and advancement within a single group

The SMC will review available safety data including safety data through the 9-day post vaccination safety monitoring period prior to enrollment in the next study group. If any of the pausing criteria in Table 2 (Section 9.8.3) are met, a study pause will be triggered. Otherwise, the next group of volunteers will be enrolled.

9.6 HIV Counseling and Testing

HIV counseling will be performed in compliance with the CDC's guidelines and local guidelines for HIV counseling, testing, and referral. Volunteers and household contacts will be counseled at all scheduled visits during the trial on the avoidance of HIV infection and on the potential negative social impacts of testing antibody positive due to the vaccine. They will also be counseled on the risks of seeking HIV antibody testing outside of the study during study participation and will be discouraged from doing so.

9.6.1 Distinguishing intercurrent HIV infection from vaccine-induced seroreactivity

The study product may elicit an antibody response to HIV proteins. Therefore, vaccine-induced seroreactivity (VISR) may occur in this study. If significant shedding of the

vaccine occurs, it is possible that household volunteers may acquire VISR as well, although considering the attenuated nature of the vector this seems unlikely. Several precautionary measures will be taken to distinguish intercurrent HIV infection from vaccine-induced positive serology.

- Signs or symptoms of an acute HIV infection syndrome, an intercurrent illness consistent with HIV-1 infection, or probable HIV exposure would prompt a diagnostic workup for recent exposure/acute infection testing to determine HIV infection.

- HIV diagnostic testing will be similar to the HIV Vaccine Trials Network (HVTN) HIV testing algorithm, which is able to distinguish vaccine-induced antibody responses from actual HIV infections.

- All volunteers or household contacts who have VISR will be offered post-study HIV-1 diagnostic testing (HIV-1 enzyme immunoassays [EIAs], Western blot, and/or PCR periodically and free of charge as medically/socially indicated (approximately every 6 months).

All volunteers who become HIV-infected during the study will be terminated from the study. Potential and enrolled volunteers identified as HIV-infected will be referred for medical treatment, counseling, and management of the HIV infection. These individuals may also be referred to appropriate ongoing clinical trials or observational studies.

9.7 Prior and Concomitant Treatment

All concomitant medications will be recorded on CRFs from the signing of the ICF until the last study visit. Study volunteers can receive medications such as acetaminophen, non-steroidal anti-inflammatory drugs (NSAIDs), or antihistamines as needed although they must be documented. Use of these medications as routine prophylaxis prior to study vaccination is discouraged.

9.8 Study discontinuation

A volunteer will be taken out of the study in the event of:

- Repeated failure to comply with protocol requirements
- Decision by the study sponsor of PI to stop or cancel the study
- Decision by local regulatory authorities or IRB to stop or cancel the study
- Volunteer’s request

9.8.1 Early discontinuation or withdrawal of volunteers

Each volunteer and household contact has the right to withdraw from the study at any time for any reason without affecting the right to treatment by the investigator/designee. Any unnecessary withdrawal from the Isolation Unit before Day 9 (day of discharge) should be strongly avoided. Prior to admission, prior to vaccination, and throughout the isolation period volunteers will be educated as to the importance of remaining on the Isolation Unit through Day 9 (day of discharge). If a volunteer decides to leave the Isolation Unit prematurely, they will be instructed to return daily for shedding samples to be collected through Day 9 post-vaccination.

The investigator should make an attempt to contact volunteers who did not return for scheduled visits or follow-up. Although the volunteer is not obliged to give reason(s) for withdrawing prematurely, the investigator should make a reasonable effort to ascertain the reason(s) while fully respecting the volunteer's rights.

The investigator also has the right to withdraw a volunteer, e.g. because of worsening health status, intercurrent illness, AEs, or pregnancy (for pregnancy follow-up see Section 10.4). The sponsor reserves the right to request the withdrawal of a volunteer due to safety issues, protocol violations, or administrative or other reasons.

Any unnecessary withdrawal should be avoided. Should a volunteer be withdrawn, all efforts should be made to complete and report the observations as thoroughly as possible. Whenever a volunteer is withdrawn from the study, independent of the reason, a final evaluation must be completed for that volunteer and the primary reason for which the volunteer was withdrawn must be stated. All documentation concerning the volunteer must be as complete as possible.

For those volunteers who are unable to continue participation in the study, but who do not withdraw consent, an early termination visit will be conducted. Any volunteer who withdraws consent will not have any further data collected after consent has been withdrawn.

9.8.2 Premature termination of the study

The sponsor reserves the right to discontinue the study for safety, ethical, or administrative reasons. Should the study be discontinued, no further vaccinations will be administered but volunteers who are still actively participating at the time of discontinuation will be followed through the remainder of their follow up visits.

9.8.3 Study pausing rules

If the trial is placed on safety pause, all enrollment and vaccinations will be suspended pending review. The AEs that may lead to a safety pause or prompt PSRT AE review are summarized below in Table 2. These AEs apply both to volunteers and household contacts. Vaccinations may be suspended for safety concerns other than those described in the table, or before pause rules are met, if, in the judgment of the PI or PSRT, volunteer or household contact safety may be threatened.

Table 2: AE notification and safety pause/AE review rules

Event and relationship to study product	Severity	Occurrence	Site PI action	PSRT or SMC action
SAE, related[1]	Any	Any	Phone, email or fax forms to sponsor within 24 hours	Study pause within 24 hours, refer to SMC for review
SAE, not related[2]	Grade 5	Any	Phone, email or fax forms to sponsor within 24 hours	PSRT review within 2 business days to consider pause
AE[3], related[4]	Grade 3 or 4[5]	Second[6]	Phone, email or fax notification to sponsor within 24 hours	Study pause within 24 hours, refer to SMC for review
AE[3], related[4]	Grade 3 or 4[5]	First	Phone, email or fax notification to sponsor within 24 hours	PSRT review within 2 business days to consider pause
Documented VISR in household contact	N/A	Any	Phone, e-mail or fax notification to sponsor within 24 hours	Study pause within 24 hours, refer to SMC for review
Documented illness compatible with Ad26 infection associated with a positive Ad26 serology in household contact[7]	N/A	Any	Phone, email or fax forms to sponsor within 24 hours	Study pause within 24 hours, refer to SMC for review

[1] Related SAE refers to SAE deemed to be definitely, probably, or possibly related to study vaccine.

[2] Not related SAE refers to SAE deemed to be probably not related or not related to the study vaccine.

[3] Does not include the following reactogenicity symptoms (fatigue/malaise, myalgia, arthralgia, chills, headache, nausea, vomiting).

[4] Related AE refers to fever, conjunctivitis, gastroenteritis, cystitis, upper respiratory infection, or other AE that is judged probably or definitely related to the vaccine.

[5] If no evidence of disease is present other than an abnormal laboratory value, the test must be repeated (entailing blood re-draw) at least one time. The verification period will be a maximum of 72 hours after initial awareness of the abnormal laboratory value. When signs and symptoms are present, repeat test will not be needed.

[6] PSRT will determine whether the reported related AE (Grade 3 or 4) is a second occurrence of a previously reported AE (Grade 3 or 4).

[7] "Positive Ad26 serology" means a *newly* positive Ad26 serology compared to enrollment that develops *after* a documented illness compatible with Ad26 infection in a household contact.

For events in the table above, the Site Principal Investigator (PI) notifies the IAVI Medical Monitor (within a few hours after the site observes, or is notified of, the AE) and the IAVI Medical Monitor then notifies the PSRT immediately (within 24 hours). The PSRT will convene within two business days to review these AEs. The PSRT will review and determine disposition (including whether the SMC needs to review the event).

If a study pause is triggered, all enrollment and vaccinations will be held until review by the SMC. Resumption of enrollment and study treatment may be determined by the SMC (in consultation with the FDA, if required) following a cumulative review of the available safety data. If a decision to resume study enrollment and study treatment

administration is made, the SMC will record its judgment in a memorandum to the study file and notify the sponsor, who will then forward the memorandum to the principal investigators. The clinical site will be allowed to resume activities upon receipt of written notification from the sponsor or its designee. As needed, the appropriate regulatory authorities will be informed in writing of the decision by the SMC to resume or discontinue study activities. The site is responsible for notifying their IRBs according to local standards and regulations. The sponsor is responsible for notifying the FDA.

9.8.4 Admission of back up volunteers

To facilitate the timely enrollment of 6 volunteers into the isolation unit at the same time, two additional volunteers may be admitted for each group as back-ups. These back-up volunteers will be admitted to the isolation unit on D-2. If back up volunteers are not needed, then they will be discharged on D0 and all collected samples will be discarded. If another volunteer should withdraw from the study before D0, then the first backup volunteer will act as his or her replacement for the duration of the study. Back up volunteers who are discharged prior to receiving vaccine/placebo are eligible for participation in a future group.

9.9 Laboratory evaluations

The total approximate volume of blood that will be collected from each volunteer is presented in Section 18.7. The total approximate blood volume that will be collected from each household contact is presented in Section 18.8. Total blood volume drawn from each volunteer will not exceed the American Association of Blood Banks (AABB), and US Food and Drug Administration (FDA) guidelines of 550 mL in any eight-week period.

All biological samples must be collected in the appropriate manner. The investigator will ensure that the personnel and laboratory under his/her supervision comply with these requirements. Further details on shipment, handling, and storage of the samples are provided in the Specimen Handling Guidelines.

Any residual samples will be stored indefinitely following completion of the study at the sponsor-designated laboratories. Any future analyses conducted on the samples will maintain volunteer anonymity. Volunteers will have to provide their approval for long-term storage of their biological specimens. Volunteers will have the right to opt out of having their biological specimens stored once all analyses in the Informed Consent Form are completed. Opting out of this procedure does not impact the volunteer’s ability to participate in the study.

9.10 Potential risks and benefits

9.10.1 Risks related to vaccines

Volunteers may exhibit general signs and symptoms associated with the oral administration of a vaccine or placebo. Side effects, if observed, are expected to be short term, mild, and not requiring treatment.

Volunteers may have an allergic reaction to the vaccination. An allergic reaction may cause a rash, hives or even difficulty breathing. Severe reactions are rare. Medications are available in the isolation facility to treat serious allergic reactions.

The effect of this vaccine on a fetus or nursing baby is unknown, as well as the effect on semen. Therefore, sexually active males (unless anatomically sterile) must be willing to use an effective method of contraception (such as consistent condom use) from the day of first vaccination until at least 4 months after vaccination. Female volunteers of child-bearing potential will be required to agree to use birth control for sexual intercourse beginning prior to the vaccination and continuing through the 4 months after vaccination. Women who are pregnant or nursing will be excluded from the study.

The vaccine may cause a positive HIV antibody test using the standard screening test. A positive or indeterminate test may have a negative employment and social impact. As a result, a letter of participation will be provided on request. Volunteers will be discouraged from donating blood during study participation as a result of the potential false-positive HIV antibody test result. If these test results should occur, Western blot analysis, PCR, and/or other required testing will be performed either to exclude or confirm HIV infection. Blood donation options for those volunteers who wish to resume blood donation will be explained at the final study follow-up visit.

9.10.2 Blood draws

Blood drawing may cause pain, bruising, and, rarely, infection at the site where the blood is taken.

9.10.3 Unknown risks

There may be other serious risks that are not known. Volunteers may believe that this vaccine provides protection against acquiring HIV infection, and therefore practice riskier behavior. They will receive extensive counseling throughout the study to address this potential problem. It is not known if the study vaccine increases, decreases, or has no effect on the chance of becoming HIV infected when exposed, or if upon becoming HIV infected, the person’s disease course progresses faster or slower to AIDS.

9.10.4 Potential benefits

There is no direct benefit to the volunteer or household contact for participation in this clinical trial. Although study volunteers may benefit from clinical testing and physical examination, they may receive no direct benefit from participation. Society may benefit from knowledge gained in this study that may aid in the development of an HIV vaccine.

10 SAFETY ASSESSMENTS

10.1 Adverse events

10.1.1 Definitions

Adverse events

An AE is any untoward medical occurrence in a patient or volunteer, and does not necessarily have a causal relationship with a medical treatment. This includes any noxious, pathological, or unintended change in anatomical, physiological, or metabolic functions, as indicated by physical signs, symptoms, and/or laboratory-detected changes. These might occur in any phase of the clinical study whether associated with the study vaccine or not. This includes exacerbations of pre-existing conditions or events, intercurrent illness, or vaccine or drug interactions. Anticipated day-to-day fluctuations of pre-existing conditions that do not represent a clinically significant exacerbation are not considered AEs. Discrete episodes of chronic conditions occurring during a study period should be reported as AEs in order to assess changes in frequency or severity.

Serious adverse events

Seriousness refers to the outcome of an AE. Seriousness is determined by both the investigator and medical monitor, and can also be determined by the sponsor (*FDA 2010 Guidance for Industry and Investigators; Safety Reporting Requirements for INDs and BA/BE Studies*). An AE or suspected adverse reaction is considered "serious" if, in the view of either the investigator or sponsor, it results in any of the following outcomes:

- Results in **death.**
- Is **life-threatening**: i.e. the volunteer was at risk of death at the time of the event; it does not refer to an event which hypothetically might have caused death if it were more severe.
- Results in persistent or significant **disability/incapacity**: i.e. results in a substantial disruption of the volunteer's ability to carry out normal life functions. This definition is not intended to include experiences of relatively minor medical significance, such as headache, nausea, vomiting, diarrhea, influenza, infusion site reactions, or accidental trauma (e.g. sprained ankle).
- Requires in-patient **hospitalization** or prolongation of existing hospitalization: i.e. the volunteer is detained (usually at least an overnight stay) at the hospital or emergency ward for treatment that would not have been appropriate in the physician's office or in an out-patient setting. Hospitalization for either elective surgery related to a pre-existing condition which did not increase in severity or frequency following initiation of the study, or for routine clinical procedures [1] (including hospitalization for 'social' reasons) that are not the result of an AE, are **not** considered as SAEs.
- Is a **congenital anomaly or birth defect** in the offspring of a study volunteer
- Is an **important medical event** that may jeopardize the volunteer or may require intervention to prevent one of the other outcomes listed above: e.g. interventions

[1] A procedure that may take place during the study period and should not interfere with the study drug administration or any of the ongoing protocol-specific procedures.

such as intensive treatment in an emergency room or at home for allergic bronchospasm; blood dyscrasias or convulsions that do not result in hospitalization, or development of drug dependency or drug abuse. Based on medical and scientific judgment, these events should usually be considered serious.

A suspected transmission of any infectious agent via a medicinal product is always considered as an important medical event, i.e. an SAE.

Although **not** considered as an SAE, cancer should be reported in the same way as SAEs.

Note: If anything untoward is reported during an elective procedure, that occurrence must be reported as an AE, either serious or non-serious according to the criteria defined above.

When in doubt as to whether hospitalization occurred or was necessary, the AE should be considered serious.

Adverse reactions
An adverse reaction is an AE judged to be related to study vaccine.

Related AEs (adverse reactions) are defined as those judged by the investigator, sponsor or independent safety monitor to be possibly, probably, or definitely related to study vaccine.

When an AE is judged to be related to study vaccine and also is judged to be serious and unexpected, it is a SUSAR (suspected unexpected serious adverse reaction) and is volunteer to expedited reporting. All SAEs in this study will be considered unexpected as there are no expected AEs associated with this vaccine product.

Adverse reactions will be reported in accordance with FDA 2009 Guidance for Clinical Investigators, Sponsors and IRBs; Adverse Events Reporting to IRBs – Improving Human Volunteer Protection.

10.1.2 Surveillance, reporting, and documentation of adverse events

The recording of AEs is an essential part of study documentation. The investigator is responsible for documenting all AEs as set out in the following sections.

10.1.2.1 Surveillance of adverse events

At each visit through four months following vaccination, all AEs, either observed by the investigator or one of his/her clinical collaborators, or reported by the volunteers spontaneously or in response to a direct question, will be evaluated by the investigator or designee. All SAEs will be reported through the final visit (Study Day 365).

Volunteers will be instructed to contact the investigator immediately should he/she experience any signs or symptoms he/she perceives as severe from the time of vaccination through a period of 12 months.

10.1.2.2 Documentation of adverse events

All AEs will be collected on case report forms (CRFs) from the signing of the ICF until Study Day 112. SAEs will be collected through Study Day 365.

AEs and SAEs should be documented in terms of signs and symptoms observed by the investigator or reported by the volunteers. Whenever possible, a medical diagnosis should be made. The nature of each event, date and (where appropriate) time of onset, outcome, severity, and causal relationship should be established. Details of any symptomatic/corrective treatment should be recorded on the appropriate page of the CRF.

Hospitalization for routine clinical procedures (or other reason) that is not the result of an AE, are not considered AEs but will be recorded on the AE page of the CRF ('Hospitalization, Not an AE'). The same applies for hospitalization for elective procedures related to a pre-existing condition that did not increase in severity or frequency during the study. If hospitalization was planned before administration of the study vaccine, it will be documented in the Medical History page of the CRF (see below).

The following events will be documented in the Medical History page of the CRF:

- Pre-existing conditions or signs and/or symptoms present in a volunteer before study start. This includes conditions that were not recognized at study entry but later during the study period.
- Hospitalization arising from a pre-existing condition and planned before the administration of the vaccine.

10.1.2.3 Post-vaccination reactions occurring immediately after each vaccination

Volunteers will be observed for minimum of 30 minutes following vaccine administration. Vital signs (temperature, blood pressure, pulse and respiratory rate) will be taken and qualified study personnel will evaluate for any signs or symptoms of reactions.

Possible reactions may include fever (≥37.7 ºC), fatigue/malaise, headache, myalgia, arthralgia, chills, nausea, and vomiting. For life-threatening allergic reactions that occur immediately post-vaccination, the site has specific procedures developed for handling such emergencies.

10.1.2.4 Reactions occurring within ten days (D0-D9) after vaccination

Volunteers will be housed in an isolation facility for 2 days prior to vaccination (Day -2), for the day of vaccination (Day 0), and for the subsequent 9 days following vaccination (D1-9). The purpose of isolation is to limit the possibility of transmission of the replication-competent viral vector. During the isolation period, study clinicians (MD, NP, or nurse) will visit each volunteer twice daily to monitor vital signs, complete scheduled lab procedures, and assess for any adverse events.

Volunteers will be instructed to notify specified study personnel immediately if any unusual or severe sign or symptom appears after vaccination.

10.1.3 SAE Reporting

IAVI has a regulatory obligation to report SAEs to the FDA according to established timetables for reporting based on specific criteria. The investigator will report SAEs to IAVI (or designee) within 24 hours from the time they first learn of the event. The SAE form will be completed as thoroughly as possible and signed by the investigator or his/her designee before transmittal to IAVI (or designee). The investigator will provide his/her assessment of causality to study treatment at the time of the initial SAE report. The investigator will not delay in the reporting of any SAE in order to obtain additional information. Any additional information, if collected, will be reported to IAVI as a follow-up to the initial report.

All SAEs must be reported immediately (within 24 hours of discovery) by email or fax to the sponsor at the following contact information:

Email: MAreport@iavi.org
Fax: 1-888-317-4215

The Medical Monitor will perform a clinical review of the information provided to identify any missing data. The Medical Monitor will also contact the study site to clarify any discrepant or missing information, to answer questions and to provide guidance to the site, if needed. The investigator will report the SAE as an acceptable medical diagnosis. If a preliminary diagnosis has not yet been made, then each symptom will be listed separately. A follow-up report will be issued when a diagnosis is made.

The investigator must report SAEs to the appropriate IRB as requested by the board according to local legal requirements.

10.2 Reporting requirements to the local IRB

The PI/designee will be responsible for providing all safety reports and reporting all SAEs, study pauses, social harms, and major deviations to the local regulatory authorities, such as a local IRB, and any regulatory agencies, in a timely manner according to the institution’s guidelines and to local regulations.

10.3 Causality assessment of study vaccine to adverse events

Every effort should be made by the investigator to explain any AE and assess its potential causal relationship, i.e. to administration of the study treatment or to alternative causes (e.g. natural history of the underlying diseases, concomitant therapy). This applies to all AEs, whether serious or non-serious.

The investigator will use the following guidelines to assess the causal relationship of an AE to study injection:

**Not related:**	Adverse experiences felt to be due to extraneous causes that neither follows a known pattern of response nor a reasonable temporal relationship to study vaccine.
**Remote (probably not related):**	Adverse experiences that are unlikely to be related to study vaccine but which follow a reasonable temporal relationship,

	such that this cannot be completely excluded or events that could be associated with study vaccine but which are unrelated in time.
**Possibly related:**	Adverse experiences that may be due to extraneous causes but which follow a known pattern of response and/or a reasonable temporal relationship to study vaccine.
**Probably related:**	Adverse experiences that cannot be explained by extraneous causes; which follow a known pattern of response and/or a reasonable temporal relationship; which disappear or decrease on cessation of study vaccine and reappear on re-challenge.
**Definitely related:**	Adverse experiences that have a definite relationship to the study vaccine (e.g. anaphylactic reaction after vaccination) without any other explained causes; which follow a known pattern of response and/or a reasonable temporal relationship; which disappear or decrease on cessation of study vaccine and reappear on re-challenge.

In the final analyses, events categorized as “not related” and “remote” will be considered as not related to study vaccine; events categorized as “possibly related”, “probably related” and “definitely related” will be considered as related to study vaccine.

10.3.1 Severity of adverse events

All AE and lab data will be coded for severity using the DAIDS Table for Grading the Severity of Adult and Pediatric Adverse Events (DAIDS AE Grading Table), **Version 1.0 dated December 2004** (Clarification, August 2009) included in Section 18.1, and also located on the website: http://rsc.tech-res.com/safetyandpharmacovigilance/.

For AEs not identified in the grading table (e.g. diagnosis of HIV infection), the following guidelines will be applied:

**Mild**	Grade 1	Symptoms causing no or minimal interference with usual social and functional activities
**Moderate**	Grade 2	Symptoms causing greater than minimal interference with usual social and functional activities
**Severe**	Grade 3	Symptoms causing inability to perform usual social and functional activities
**Potentially life-threatening**	Grade 4	Symptoms causing inability to perform basic self-care functions OR medical or operative intervention indicated to prevent permanent impairment, persistent disability
**Fatal**	Grade 5	For any AE where the outcome is death, the severity of the AE is classified as Grade 5

The clinical research site team will ascertain accurate recording of all AEs during the study. AE CRFs will be completed by the clinical research site staff on a daily basis as the data become available from the isolation unit, clinic or laboratory.

The investigators will monitor and analyze study data including all AE and lab data as they become available and will make determinations regarding the severity of the adverse experiences and their relation to study vaccine. AEs will be deemed either related to study vaccine or not related to study vaccine. To ensure that all AEs are captured in a timely manner, CRFs will be entered in real-time entry, and volunteered to analysis to identify AEs that may invoke study pausing rules.

The PI or designee must review AE CRFs to insure prompt and complete identification of all events that require expedited reporting as SAEs, study pausing rules or other serious and unexpected events.

Study vaccine related AEs will be followed by the clinical research site team through resolution or until study completion. Non-study vaccine related AEs will be followed to resolution or study completion, whichever occurs first.

10.3.2 Follow-up of ongoing adverse events and assessment of outcome

10.3.2.1 Follow-up of non-serious adverse events

Non-serious AEs already documented in the CRF at a previous assessment and designated as ‘ongoing’ should be reviewed at subsequent visits. If the event has resolved, the documentation in the CRF should be completed. If the frequency or severity of a non-serious AE changes significantly, a new record of the AE has to be started. If the AE becomes serious, the procedures for reporting of SAEs have to be followed (see Section 9.8.3).

New non-serious AEs will be recorded through Month 4 post-vaccination. Ongoing non-serious AEs will be monitored until the Day 365 visit (or until the last study visit for volunteers who withdraw early (see Section 9.8.1)).

Outcomes will be assessed as:

1. Resolved
2. Resolved with sequelae
3. Resolving
4. Not resolved
5. Fatal
6. Unknown

Clinically significant abnormal laboratory values will be followed up until they have returned to normal, stabilized, or a satisfactory explanation has been provided.

10.3.2.2 Follow-up of serious adverse events

All SAEs must be followed-up until the event has either resolved, subsided, stabilized, disappeared, or is otherwise explained, or the study volunteer is lost to follow-up, but no longer than 12 months after the administration of the study vaccine. Outcomes are assessed as above.

All follow-up activities must be reported in a timely manner to the IAVI Medical Monitor (if necessary on one or several consecutive SAE report forms). All form fields with additional or changed information must be completed and the SAE Report Form should be forwarded to the IAVI Medical Monitor as soon as possible but at the latest within 7 calendar days after receipt of the new information.

Clinically significant laboratory abnormalities reported as SAEs will be followed-up until they have returned to normal, or a satisfactory explanation has been provided.

Reports related to the subsequent course of any SAE reported for any volunteer must be submitted to the sponsor.

10.3.3 Treatment of adverse events

Treatment of any AE is at the sole discretion of the investigator and according to currently available best treatment. The applied measures should be recorded in the CRF.

10.4 Handling of pregnancy cases

Pregnancy events will be reported through 4 months following vaccination. All initial reports of pregnancy must be reported to the IAVI Medical Monitor by the investigational staff within 24 hours of their knowledge of the event using the appropriate pregnancy notification form. Abnormal pregnancy outcomes (e.g. spontaneous abortion, stillbirth, and congenital anomaly) are considered SAEs and must be reported using the Serious Adverse Event Form.

Because the effect of the study vaccine on sperm is unknown, pregnancies in partners of male volunteers included in the study will be reported by the investigational staff within 24 hours of their knowledge of the event using the appropriate pregnancy notification form.

Follow-up information regarding the outcome of the pregnancy and any postnatal sequelae in the infant will be required.

10.5 Physical examination

Physical examinations will be performed by the investigator or designated medically-trained clinician. The time points of these examinations are specified in Section 18.5. Any abnormalities or changes should be documented in the source document and recorded on the CRF.

A new, clinically significant finding (in the opinion of the investigator) not noted at screening must be captured as an AE. In addition, resolution of any abnormal findings during the study will be noted in the source document and in the CRF.

10.6 Routine safety laboratory

Samples for routine safety laboratory parameters will be collected at the time points specified in Section 18.7.

The following routine safety laboratory parameters will be determined:

- **Serum chemistry:** AST, ALT, and Creatinine
- **Complete blood count:** hemoglobin, hematocrit, white blood cells (WBC), WBC differentiation, red blood cell count (RBC), and platelet count

Laboratory values will be graded according to the DAIDS toxicity scale (Section 18.1) and, if clinically significant, reported as AEs.

10.7 Vital signs

Vital sign measurements will be performed at time points specified in Section 18.5.

The following measurements will be performed:

- Heart rate (bpm)
- Systolic and diastolic blood pressure (mmHg)
- Respiratory rate
- Body temperature (oral)

A confirmatory vital signs measurement can be performed if inconsistent with a prior measurement. If any clinically significant changes in vital signs are noted, they will be reported as AEs and followed to resolution, or until reaching a clinically stable endpoint.

10.8 Monitoring for HIV infection

Diagnostic HIV testing will be performed for both volunteers and household contacts during the study as indicated in Sections 18.5 and 18.6. Information provided to the clinical staff of the trial site will not include the results of specific tests, but will state only the final interpretation as “infected” or “uninfected”. This system allows timely HIV testing without compromising the double-blind nature of the trial. Volunteers or household

contacts testing positive on HIV testing will be called back for confirmatory testing and counseling. Once confirmed infected, the volunteer will be referred for treatment.

10.8.1 Management of volunteers who become HIV-infected during the study

The individual HIV test results performed to confirm the diagnosis will be forwarded to the study staff. Volunteers who become HIV-infected during the trial will be terminated from the study and will be counseled and referred for medical treatment.

10.9 Protocol Safety Review Team and Safety Monitoring Committee

The Protocol Safety Review Team (PSRT) will include the Protocol Chair, Protocol Co-Chairs, Site Principal Investigator, co-investigators and the IAVI Medical Monitor. Additional members could include associate investigators, senior clinical research nursing staff, and site staff. The PSRT will decide by consensus whether AEs not requiring a study pause should also be reviewed by the SMC.

The Safety Monitoring Committee (SMC) will be appointed by the sponsor before the start of the study and will operate according to its charter. The SMC is an independent multidisciplinary group consisting of experts in human immunodeficiency virus (HIV) or adenoviruses that, collectively, has experience in the management, conduct, and monitoring of vaccine trials. Members of the SMC are not directly affiliated with this protocol and are not situated at the study site. The SMC will review safety data, unblinded as to treatment arm as needed, for D0-D9 following vaccination of all volunteers in each Group before deciding whether or not to proceed with dose escalation. The SMC also will review individual Expedited Adverse Event (EAE) reports. The SMC will conduct additional special reviews at the request of the PSRT.

11 IMMUNOGENICITY AND VIRAL SHEDDING ASSAYS

11.1 HIV-associated immunogenicity

Blood samples for the determination of cellular and humoral responses will be collected as specified in Section 18.7.

Evaluations of cellular immune responses to HIV will likely include, but are not limited to, the following assays: IFN-γ ELISPOT, intra-cellular cytokine staining (ICS), and epitope mapping as indicated in Table 3.

Table 3: Cellular immune response assays

Rationale	Objective/ endpoint	System	Assay method	Unit	Timepoint(s)
T-cell responses	Secondary	PBMC	Interferon gamma producing cells (ELISPOT)	Number of spot forming cells per 10^6 PBMC	Baseline, 2, 4, 16, 34, and 52 weeks
	Secondary	PBMC	Intracellular cytokine staining (ICS)	Number of cytokine(s) + cells per 10^6 CD4/CD8 T cells	As above
	Exploratory	PBMC	Epitope mapping	# Epitopes	4 weeks following vaccination

Humoral response assays to HIV will likely include, but are not limited to: ELISA and neutralizing Ab, as indicated in Table 4.

Table 4: Humoral immune response assays

Rationale	Objective/ endpoint	System	Assay method	Unit	Timepoint(s)
Humoral responses	Secondary	Serum	Env binding Antibody (ELISA)	Endpoint titer	Baseline, 2, 4, 16, 34, and 52 weeks
	Secondary	Serum	HIV neutrAb (tzm-bl and/or A3R5)	Tier 1 and Tier 2 NAbs	As above

Immunologic assays may be performed and analyzed in a blinded fashion after all volunteers of a given group reach Day 112. This will allow more rapid accrual of data to facilitate product development strategies. The optimal choice of immunogenicity assays will take into consideration the latest technological advances.

11.2 Anti-vector immunity

Samples collected may also be assessed for the development of anti-vector immunity to Ad26, including Ad26-specific binding antibodies by ELISA as well as neutralizing antibodies.

11.3 DNA/RNA Assays

Blood samples collected for cellular immunogenicity may also be used for exploratory DNA and RNA micro-array and deep-sequencing assays.

11.4 Additional Immunogenicity Assays

Additional immunogenicity assessments of systemic and mucosal responses may potentially include the following: HIV-1-specific antibody-dependent cell-mediated cytotoxicity (ADCC) and cell-mediated viral inhibition assays (ADCVI), multiparameter intracellular cytokine staining and flow cytometry of CD4+ and CD8+ T lymphocytes, extracellular cytokine secretion by multiplex cytokine bead array, in vitro CD8+ T cell-mediated inhibition of HIV-1, proliferative capacity of T lymphocytes, B cell repertoire analysis, gene expression profiling, and/or rectal wicks for analysis of mucosal humoral immune responses.

11.5 Viral shedding assays

Samples collected for analysis of viral shedding will be assessed by (rt)PCR and viral culture.

Table 5: Viral shedding assays

Rationale	Objective/ endpoint	System	Assay method	Unit	Timepoint(s)
Vector shedding	Secondary	Throat and rectal swabs/ Plasma	Real-time PCR	Copies/ml	Baseline (Day 0), days 1-9, 12, 17, 21, 28<br>Plasma PCR Day 7 only
	Secondary	Throat and rectal swabs	Adenovirus culture	Cytopathic effect (CPE)	Performed whenever PCR is positive

Rectal and throat swabs will be assessed for rcAd26.MOS1.HIV-Env by PCR as outlined in Schedule of Procedures (Section 18.5); if a rectal or throat swab is positive by PCR, then adenovirus culture will also be performed. Plasma adenovirus PCR will be performed on day 7 but results will not be available prior to discharge from the isolation unit; if plasma adenovirus PCR is positive by PCR, then adenovirus culture will also be performed.

Real-time (rt)PCR analysis will be performed for both the Ad26 vector and the Env insert to allow for quantitation of vector and insert and to evaluate the stability of the vaccine. If the PCR is positive, adenovirus cultures will also be performed to evaluate for the presence of infectious virus and to allow eventual correlation of PCR and culture results; PCR will be performed on positive cultures to confirm that the virus is rcAd26.MOS1.HIV-Env.

All shedding samples will be frozen, and all PCR and cultures will be performed at CVVR. Investigators and study clinicians will be blinded to adenovirus PCR and culture results, but a single individual on the study team will be unblinded to gain access to the

results from day 28. If a volunteer's adenovirus PCR remains positive at day 28, weekly adenovirus PCR will be performed until the PCR is negative.

12 DATA EVALUATION AND STATISTICS

All data entry will be performed by qualified and trained study staff. When all data have been entered and validated, the final database will be locked.

12.1 Analysis populations and data sets

12.1.1 Safety population

All volunteers who received rcAd26.MOS1.HIV-Env or placebo, and for whom any post-dose data is available, will be included in the safety population.

12.1.2 Immunogenicity population

The immunogenicity population will consist of all volunteers who received rcAd26.MOS1.HIV-Env or placebo, and who have at least one measured post-dose blood sample collected.

12.2 Endpoints

Safety and tolerability:

- Any adverse events within 4 months following vaccination (SAEs within 12 months following vaccination)
- Moderate or greater unsolicited adverse events related to study product (i.e., suspected adverse reactions) within 4 weeks post-vaccination
- Moderate or greater solicited reactions within 9 days post-vaccination

Shedding:

- Proportion (95% CI) of volunteers per dose group with positive responses and mean response per group with 95% CI.
    - Rectal/oral shedding: real-time PCR for rcAd26.MOS1.HIV-Env at 1, 2, 3, 4, 5, 6, 7, 8, 9, 12, 17, 21, 7, 12, 21, and 28 days after vaccination.

Immunogenicity:

- Proportion (95% CI) of volunteers per dose group with positive responses and mean response (e.g. GMT) per group with 95% CI.
    - Cellular immunogenicity: Env-specific ELISPOT responses at 4 weeks after vaccination.
    - Humoral immunogenicity: Env-specific binding antibody titers at 4 weeks after vaccination.

- Durability of responses: immunogenicity assays at 4, 8 and 12 months after vaccination.

12.3 Sample size consideration

The number of volunteers chosen for this study will provide a preliminary safety and immunogenicity assessment. The sample size is not based on formal hypothesis testing considerations, but within the range of volunteers (i.e., 20-80) recommended in the Code of Federal Regulations (CFR 312.21) for first-in-human product Phase 1 evaluations. Placebo recipients are included for blinding and safety purposes and will provide additional control specimens for immunogenicity assays. In particular, if no events are observed in a group of 4 or 5 volunteers, the upper 95% Clopper-Pearson confidence limit on the true rate of events is 60.2% and 52.2%, respectively.

13 ETHICAL AND LEGAL REQUIREMENTS

13.1 General requirements

The study will be performed according to this Study Protocol and in compliance with the Declaration of Helsinki, the guidelines of the International Conference on Harmonization (ICH) Good Clinical Practice (GCP) and the respective local legal requirements including the following: US Code of Federal Regulations 45CFR Pt 46; 21CFR Pt 50, 21CFR Pt 56 and 21CFR Pt 312.

13.2 Institutional Review Board/Ethics Committee

Before the start of the study, the investigator will submit the Study Protocol, Informed Consent Form, and other study-related documents as required by applicable laws and regulations to the responsible IRB for written approval.

The investigator will inform the IRB according to applicable laws and regulations about Amendments to the Study Protocol including, but not limited to, any new information that requires an ethical reconsideration of the Study Protocol.

Unless otherwise instructed by the IRB or local regulation the investigator must submit to the IRB:

- All subsequent Amendments to the Study Protocol, changes to the Informed Consent Form or revisions of other documents originally submitted for review
- New or revised volunteer recruiting materials approved by the sponsor, if applicable
- All subsequent changes of logistical or administrative aspects (for information)
- Serious and/or unexpected AEs occurring during the study, where required
- New information that may affect adversely the safety of the volunteers or the conduct of the study
- New edition(s) of the Investigator's Brochure and amendments/addenda
- Annual update and/or request for re-approval, where required
- Date of study completion, where required

13.3 Regulatory authorities

Before initiating the study, the sponsor will submit any required application to the regulatory authorities and obtain approval according to applicable laws and regulations. The sponsor will also inform the regulatory authorities about Amendments to the Study Protocol including, but not limited to, any new information that requires an ethical reconsideration of the Study Protocol.

13.4 Volunteer information and informed consent

The study informed consent describes the investigational products to be used and all aspects involved in protocol participation. A properly executed written informed consent, in compliance with the Declaration of Helsinki, guidelines of the Council of International Organization of Medical Sciences (CIOMS), the Belmont Report, the US Code of Federal Regulations 21 CFR 50, must be obtained from each volunteer prior to entry into the trial or prior to performing any unusual or non-routine procedure that involves risk to the volunteer. The investigator must provide a copy of the approved informed consent to the volunteer and a signed copy must be maintained in the volunteer's record file. Before a volunteer's participation in the study, it is the investigator's responsibility to obtain this written informed consent from the volunteer, after adequate explanation of the aims, methods, anticipated benefits, and potential hazards of the study and before any protocol-specific procedures or study medications are administered.

All items must be explained by the investigator or designee in a language that is easy to understand. Volunteers will also be informed that the participation is voluntary and that they have the right to withdraw at any time without giving reasons and without any disadvantages for their subsequent care. Volunteers will confirm their consent in writing before study start and any study-specific procedure.

Volunteers must be given enough time to consider participation in the study.

13.5 Indemnity

The Sponsor and Institution are responsible to have appropriate liability insurance. For research-related injuries and/or medical problems determined to result from receiving the Investigational Product, treatment including necessary emergency treatment and proper follow-up care will be made available to the volunteer free of charge at the expense of the Sponsor.

13.6 Data access and protection

Confidentiality is extended to cover testing of biological samples in addition to the clinical information relating to participating volunteers. The study protocol, documentation, data, and all other information generated will be held in strict confidence.

The collection and processing of personal data from volunteers enrolled in this study will be limited to those data that are necessary to fulfill the objectives of the study.

These data must be collected and processed with adequate precautions to ensure confidentiality and compliance with applicable data privacy protection laws and regulations. Appropriate technical and organizational measures to protect the personal data against unauthorized disclosures or access, accidental or unlawful destruction, or accidental loss or alteration must be put in place. Sponsor personnel whose

responsibilities require access to personal data agree to keep the identity of volunteers confidential.

The informed consent includes explicit consent for the processing of personal data and for the investigator/institution to allow direct access to the volunteer's source data/documents for study-related monitoring, audit, IRB review, and regulatory inspection. This consent also addresses the transfer of the data to other entities and to other countries.

The volunteer has the right to request through the investigator access to his or her personal data and the right to request rectification of any data that are not correct or complete. Reasonable steps will be taken to respond to such a request, taking into consideration the nature of the request, the conditions of the study, and the applicable laws and regulations.

13.7 Future use and storage and blood samples

Each study volunteer will be asked to consent voluntarily for their blood samples to be stored for other research studies that may be done after this study is completed. Future testing may involve DNA/RNA tests. For volunteers unwilling to have their blood samples stored for future use, they can consent to participate in this study only, without having their blood samples stored for future testing. In this case, their blood samples will be destroyed after all the tests specified for this study have been concluded.

All samples for which consent has been obtained, and for which additional material is available after study-specified testing is complete, will be stored for future testing. All applicable approvals will be sought before any such samples are used for analysis not specified in the protocol or a protocol amendment approved by the IRB.

13.8 Reimbursement

Volunteers and household contacts will be reimbursed for time and inconvenience in accordance with the standards and legal obligations for compensation required by each site. Any applicable guidelines by IRBs/ECs for compensation of research volunteers and household contacts will be sought and followed.

14 QUALITY CONTROL AND QUALITY ASSURANCE

14.1 Monitoring

Monitoring visits to the clinic will be made regularly to ensure that the study is carried out according to this Study Protocol and in compliance with GCP and applicable legal requirements.

Source documents will be reviewed for verification of consistency with CRF data. The investigator guarantees direct access to source documents for monitoring purposes. Source data verification will be performed in accordance with data protection regulations and guidelines. All information reviewed will be handled according to these rules and regulations.

The monitor will review each volunteer's data as outlined in the study specific Clinical Monitoring Plan.

14.2 Audit and inspections

Audits and inspections may be carried out by qualified delegates authorized by the sponsor or by authorities. The investigator consents to cooperate and to allow direct access to all source documents and other study-related data during an audit or inspection. All information disclosed will be handled in accordance with applicable data protection rules and regulations.

14.3 Data quality assurance

All CRF data will be entered into a validated, 21CFR Part 11 compliant, computerized clinical data management system.

The site is required to have a plan in place for assuring the quality of the research being conducted.

15 DOCUMENTATION, ARCHIVING, AND PUBLICATIONS

15.1 Documentation

Source documents are original documents, data, and records from which the volunteer's data are obtained. These include but are not limited to hospital records, clinical and office charts, laboratory and pharmacy records, radiographs, and correspondence.

The Investigator and staff are responsible for ensuring maintenance of a comprehensive and centralized filing system of all study-related (essential) documentation, suitable for inspection at any time by representatives from the sponsor, FDA, and/or applicable regulatory authorities.

All CRF entries have to be verifiable by the source data in the volunteer file. This does not apply to CRF entries that are defined as source data.

15.2 Archiving

The Investigator is responsible for the archiving of the Investigator's file, the volunteer's file, and the source data according to national and international legal requirements.

Any records related to the conduct of the study may not be destroyed without written authorization by the sponsor. According to GCP requirements, study records should be retained until at least 2 years after the last approval of a marketing application in an ICH region and until there are no pending or contemplated marketing applications in an ICH region or at least 2 years have elapsed since the formal discontinuation of clinical development of the investigational product.

15.3 Clinical Study Reports and Publications

The results of the clinical study will be documented in the Clinical Study Report.
The study results may be published and/or presented at scientific meetings. Terms of publication will be addressed in an agreement between the sponsor and participating partners.

16 REFERENCES

1. Centers for Disease C, Prevention. Vital signs: HIV prevention through care and treatment--United States. MMWR 2011;60:1618-23.

2. UNAIDS. Report on the Global AIDS Epidemic. 2013.

3. Centlivre M, Sala M, Wain-Hobson S, Berkhout B. In HIV-1 pathogenesis the die is cast during primary infection. AIDS 2007;21:1-11.

4. Wawer MJ, Gray RH, Sewankambo NK, et al. Rates of HIV-1 transmission per coital act, by stage of HIV-1 infection, in Rakai, Uganda. J Infect Dis 2005;191:1403-9.

5. Flynn NM, Forthal DN, Harro CD, et al. Placebo-controlled phase 3 trial of a recombinant glycoprotein 120 vaccine to prevent HIV-1 infection. J Infect Dis 2005;191:654-65.

6. Pitisuttithum P, Gilbert P, Gurwith M, et al. Randomized, double-blind, placebo-controlled efficacy trial of a bivalent recombinant glycoprotein 120 HIV-1 vaccine among injection drug users in Bangkok, Thailand. J Infect Dis 2006;194:1661-71.

7. Gray GE, Allen M, Moodie Z, et al. Safety and efficacy of the HVTN 503/Phambili study of a clade-B-based HIV-1 vaccine in South Africa: a double-blind, randomised, placebo-controlled test-of-concept phase 2b study. Lancet Infect Dis 2011;11:507-15.

8. Buchbinder SP, Mehrotra DV, Duerr A, et al. Efficacy assessment of a cell-mediated immunity HIV-1 vaccine (the Step Study): a double-blind, randomised, placebo-controlled, test-of-concept trial. Lancet 2008;372:1881-93.

9. Rerks-Ngarm S, Pitisuttithum P, Nitayaphan S, et al. Vaccination with ALVAC and AIDSVAX to prevent HIV-1 infection in Thailand. N Engl J Med 2009;361:2209-20.

10. Haynes BF, Gilbert PB, McElrath MJ, et al. Immune-correlates analysis of an HIV-1 vaccine efficacy trial. N Engl J Med 2012;366:1275-86.

11. Zolla-Pazner S, deCamp AC, Cardozo T, et al. Analysis of V2 antibody responses induced in vaccinees in the ALVAC/AIDSVAX HIV-1 vaccine efficacy trial. PLoS One 2013;8:e53629.

12. Barouch DH, Liu J, Li H, et al. Vaccine protection against acquisition of neutralization-resistant SIV challenges in rhesus monkeys. Nature 2012;482:89-93.

13. Barouch DH, Stephenson KE, Borducchi EN, et al. Protective efficacy of a global HIV-1 mosaic vaccine against heterologous SHIV challenges in rhesus monkeys. Cell 2013;155:531-9.

14. Parks CL, Picker LJ, King CR. Development of replication-competent viral vectors for HIV vaccine delivery. Curr Opin HIV AIDS 2013;8:402-11.

15. Mandell GL, Bennett JE, Dolin R. Mandell, Douglas, and Bennett's principles and practice of infectious diseases. 7th ed. Philadelphia, PA: Churchill Livingstone/Elsevier; 2010.

16. Joag SV, Liu ZQ, Stephens EB, et al. Oral immunization of macaques with attenuated vaccine virus induces protection against vaginally transmitted AIDS. J Virol 1998;72:9069-78.

17. Daniel MD, Kirchhoff F, Czajak SC, Sehgal PK, Desrosiers RC. Protective effects of a live attenuated SIV vaccine with a deletion in the nef gene. Science 1992;258:1938-41.

18. Wyand MS, Manson K, Montefiori DC, Lifson JD, Johnson RP, Desrosiers RC. Protection by live, attenuated simian immunodeficiency virus against heterologous challenge. J Virol 1999;73:8356-63.

19. Cranage MP, Whatmore AM, Sharpe SA, et al. Macaques infected with live attenuated SIVmac are protected against superinfection via the rectal mucosa. Virology 1997;229:143-54.

20. Girard MP, Osmanov S, Assossou OM, Kieny MP. Human immunodeficiency virus (HIV) immunopathogenesis and vaccine development: a review. Vaccine 2011;29:6191-218.

21. Hansen SG, Piatak M, Jr., Ventura AB, et al. Immune clearance of highly pathogenic SIV infection. Nature 2013;502:100-4.

22. George MD, Asmuth DM. Mucosal immunity in HIV infection: what can be done to restore gastrointestinal-associated lymphoid tissue function? Curr Opin Infect Dis 2014.

23. Masopust D, Picker LJ. Hidden memories: frontline memory T cells and early pathogen interception. J Immunol 2012;188:5811-7.

24. Baden LR, Liu J, Li H, et al. Induction of HIV-1-specific mucosal immune responses following intramuscular recombinant adenovirus serotype 26 HIV-1 vaccination of humans. J Infect Dis 2014.

25. Barouch DH, Kik SV, Weverling GJ, et al. International seroepidemiology of adenovirus serotypes 5, 26, 35, and 48 in pediatric and adult populations. Vaccine 2011;29:5203-9.

26. Mast TC, Kierstead L, Gupta SB, et al. International epidemiology of human pre-existing adenovirus (Ad) type-5, type-6, type-26 and type-36 neutralizing antibodies: correlates of high Ad5 titers and implications for potential HIV vaccine trials. Vaccine 2010;28:950-7.

27. Li H, Rhee EG, Masek-Hammerman K, Teigler JE, Abbink P, Barouch DH. Adenovirus serotype 26 utilizes CD46 as a primary cellular receptor and only transiently activates T lymphocytes following vaccination of rhesus monkeys. J Virol 2012;86:10862-5.

28. Waddington SN, McVey JH, Bhella D, et al. Adenovirus serotype 5 hexon mediates liver gene transfer. Cell 2008;132:397-409.

29. Perreau M, Welles HC, Pellaton C, et al. The number of Toll-like receptor 9-agonist motifs in the adenovirus genome correlates with induction of dendritic cell maturation by adenovirus immune complexes. J Virol 2012;86:6279-85.

30. Penaloza-Macmaster P, Provine NM, Ra J, et al. Alternative serotype adenovirus vaccine vectors elicit memory T cells with enhanced anamnestic capacity compared to ad5 vectors. J Virol 2013;87:1373-84.

31. Tan WG, Jin HT, West EE, et al. Comparative analysis of simian immunodeficiency virus Gag-specific effector and memory CD8+ T cells induced by different adenovirus vectors. J Virol 2013;87:1359-72.

32. Letvin NL, Rao SS, Montefiori DC, et al. Immune and genetic correlates of vaccine protection against mucosal infection by SIV in monkeys. Sci Transl Med 2011;3:81ra36.

33. Casimiro DR, Wang F, Schleif WA, et al. Attenuation of simian immunodeficiency virus SIVmac239 infection by prophylactic immunization with dna and recombinant adenoviral vaccine vectors expressing Gag. J Virol 2005;79:15547-55.

34. Walker BD, Ahmed R, Plotkin S. Use both arms to beat HIV. Nature Medicine 2011;17:1194-5.

35. Picker LJ, Hansen SG, Lifson JD. New paradigms for HIV/AIDS vaccine development. Ann Rev Med 2012;63:95-111.

36. Stephenson KE, Barouch DH. A Global approach to HIV-1 development. Immunological Rev 2013;254:295-304.

37. Ndung'u T, Weiss RA. On HIV diversity. AIDS 2012;26:1255-60.

38. Taylor BS, Sobieszczyk ME, E. MF, Hammer SM. The challenge of HIV-1 subtype diversity. N Engl J Med 2008;358:1590-602.

39. Gaschen B, Taylor J, Yusim K, Foley B, Gao F, Lang D. Diversity considerations in HIV-1 vaccine selection. Science 2002;296:2354-60.

40. Korber BT, Foley B, Gaschen B, Kuiken C. Epidemiological and immunological implications of the global variability of HIV-1. Retroviral immune response and restoration. Totowa, New Jersey: Humana Press; 2001:1-32.

41. Korber BT, Letvin NL, Haynes BF. T-cell vaccine strategies for human immunodeficiency virus, the virus with a thousand faces. J Virol 2009;83:8300-14.

42. Barouch DH, Korber B. HIV-1 vaccine development after Step. Ann Rev Med 2009;61:2.1-2.15.

43. Hemelaar J, Gouws E, Ghys PD, Osmanov S. Global and regional distributions of HIV-1 genetic subtypes and recombinants in 2004. AIDS 2006;20:W13-23.

44. Korber B, Gaschen B, Yusim K, Thakallapally R, Kesmir C, Detours V. Evolutionary and immunological implications of contemporary HIV-1 variation. Brit Med Bull 2001;58:19-42.

45. Thurmond J, Yoon H, Juiken C, et al. Web-based design and evaluation of T-cell vaccine candidates. Bioinformatics 2008;24:1639-40.

46. Fischer W, Perkins S, Theiler J, et al. Polyvalent vaccines for optimal coverage of potential T-cell epitopes in global HIV-1 variants. Nature Med 2007;13:100-6.

47. Stephenson KE, SanMiguel A, Simmons NL, et al. Full-length HIV-1 immunogens induce greater magnitude and comparable breadth of T lymphocyte responses to conserved HIV-1 regions compared with conserved-region-only HIV-1 immunogens in rhesus monkeys. J Virol 2012;86:11434-40.

48. Barouch DH, O'Brien KL, Simmons NL, et al. Mosaic HIV-1 vaccines expand the breadth and depth of cellular immune responses in rhesus monkeys. Nat Med 2010;16:319-23.

49. Santra S, Liao HX, Zhang R, et al. Mosaic vaccines elicit CD8+ T lymphocyte responses that confer enhanced immune coverage of diverse HIV strains in monkeys. Nat Med 2010;16:324-8.

50. Fischer W, Perkins S, Theiler J, et al. Polyvalent vaccines for optimal coverage of potential T-cell epitopes in global HIV-1 variants. Nat Med 2007;13:100-6.

51. Barouch DH, O'Brien KL, Simmons NL, et al. Mosaic HIV-1 vaccines expand the breadth and depth of cellular immune responses in rhesus monkeys. Nat Med 2010;16:319-23.

52. Santra S, Liao HX, Zhang R, et al. Mosaic vaccines elicit CD8+ T lymphocyte responses that confer enhanced immune coverage of diverse HIV strains in monkeys. Nat Med 2010;16:324-8.

53. Janes H, Frahm N, DeCamp A, et al. MRKAd5 HIV-1 Gag/Pol/Nef vaccine-induced T-cell responses inadequately predict distance of breakthrough HIV-1 sequences to the vaccine or viral load. PLoS One 2012;7:e43396.

54. Gurwith M, Lock M, Taylor EM, et al. Safety and immunogenicity of an oral, replicating adenovirus serotype 4 vector vaccine for H5N1 influenza: a randomised, double-blind, placebo-controlled, phase 1 study. Lancet Infect Dis 2013;13:238-50.

55. Kong WP, Wu L, Wallstrom TC, et al. Expanded breadth of the T-cell response to mosaic human immunodeficiency virus type 1 envelope DNA vaccination. J Virol 2009;83:2201-15.

56. Roederer M, Keele BF, Schmidt SD, et al. Immunological and virological mechanisms of vaccine-mediated protection against SIV and HIV. Nature 2014;505:502-8.

57. Bell JA, Huebner RJ, Rosen L, et al. Illness and microbial experiences of nursery children at junior village. Am J Hygiene 1961;74:267-92.

58. Rhee EG, Barouch DH. Adenoviruses. In: Mandell GL, Bennett JE, Dolin R, eds. Principles and Practice of Infectious Diseases. 7th ed. Philadelphia, PA: Elsevier; 2010.

59. Brandt CD, Kim HW, Vargosko AJ, et al. Infections in 18,000 infants and children in a controlled study of respiratory tract disease. I. Adenovirus pathogenicity in relation to serologic type and illness syndrome. Am J Epidemiology 1969;90:484-500.

60. Fox JP, Brandt CD, Wassermann FE, et al. The virus watch program: a continuing surveillance of viral infections in metropolitan New York families. VI. Observations of adenovirus infections: virus excretion patterns, antibody response, efficiency of surveillance, patterns of infections, and relation to illness. Am J Epidemiology 1969;89:25-50.

61. Fox JP, Hall CE, Cooney MK. The Seattle Virus Watch. VII. Observations of adenovirus infections. Am J Epidemiology 1977;105:362-86.

62. Noel J, Mansoor A, Thaker U, Herrmann J, Perron-Henry D, Cubitt WD. Identification of adenoviruses in faeces from patients with diarrhoea at the Hospitals for Sick Children, London, 1989-1992. J Med Virol 1994;43:84-90.

63. Faden H, Wilby M, Hainer ZD, et al. Pediatric adenovirus infection: relationship of clinical spectrum, seasonal distribution, and serotype. Clin Ped 2011;50:483-7.

64. Abbas KZ, Lombos E, Duvvuri VR, Olsha R, Higgins RR, Gubbay JB. Temporal changes in respiratory adenovirus serotypes circulating in the greater Toronto area, Ontario, during December 2008 to April 2010. Virol J 2013;10:15.

65. Barouch DH, Kik SV, Weverling GJ, et al. International seroepidemiology of adenovirus serotypes 5, 26, 35, and 48 in pediatric and adult populations. Vaccine 2011;29:5203-9.

66. Diarrhea: Why children are still dying and what can be done. New York, NY: The United Nations Chidlren's Fund (UNICEF)/World Health Organization (WHO); 2009.

67. Ramani S, Kang G. Viruses causing childhood diarrhoea in the developing world. Curr Opinion Infect Dis 2009;22:477-82.

68. Kotloff KL, Nataro JP, Blackwelder WC, et al. Burden and aetiology of diarrhoeal disease in infants and young children in developing countries (the Global Enteric Multicenter Study, GEMS): a prospective, case-control study. Lancet 2013;382:209-22.

69. Magwalivha M, Wolfaardt M, Kiulia NM, van Zyl WB, Mwenda JM, Taylor MB. High prevalence of species D human adenoviruses in fecal specimens from Urban Kenyan children with diarrhea. J Med Virol 2010;82:77-84.

70. Sriwanna P, Chieochansin T, Vuthitanachot C, Vuthitanachot V, Theamboonlers A, Poovorawan Y. Molecular characterization of human adenovirus infection in Thailand, 2009-2012. Virol J 2013;10:193.

71. Liu L, Qian Y, Zhang Y, Deng J, Jia L, Dong H. Adenoviruses associated with acute diarrhea in children in Beijing, China. PLoS One 2014;9:e88791.

72. Lu QB, Tong YG, Wo Y, et al. Epidemiology of human adenovirus and molecular characterization of human adenovirus 55 in China, 2009-2012. Influenza Other Respir Viruses 2014;8:302-8.

73. Liu LY, Qian Y, Zhang Y, Jia LP, Dong HJ, Deng J. [Investigation of adenovirus infection in hospitalized children with diarrhea during 2010 in Beijing, China]. Chinese J Ped 2012;50:450-4.

74. Ouyang Y, Ma H, Jin M, et al. Etiology and epidemiology of viral diarrhea in children under the age of five hospitalized in Tianjin, China. Arch Virol 2012;157:881-7.

75. Lee JI, Lee GC, Chung JY, et al. Detection and molecular characterization of adenoviruses in Korean children hospitalized with acute gastroenteritis. Microbiol Immunol 2012;56:523-8.

76. Espinola EE, Russomando G, Basualdo W, et al. Genetic diversity of human adenovirus in hospitalized children with severe acute lower respiratory infections in Paraguay. J Clin Virol 2012;53:367-9.

77. Mast TC, Kierstead L, Gupta SB, et al. International epidemiology of human pre-existing adenovirus (Ad) type-5, type-6, type-26 and type-36 neutralizing antibodies: correlates of high Ad5 titers and implications for potential HIV vaccine trials. Vaccine 2010;28:950-7.

78. Kasel JA, Evans HE, Spickard A, Knight V. Conjunctivitis and enteric infection with adenovirus types 26 and 27: responses to primary, secondary and reciprocal cross-challenges. Amer J Hygiene 1963;77:265-82.

79. Hierholzer JC, Wigand R, Anderson LJ, Adrian T, Gold JW. Adenoviruses from patients with AIDS: a plethora of serotypes and a description of five new serotypes of subgenus D (types 43-47). J Infect Dis 1988;158:804-13.

80. Khoo SH, Bailey AS, de Jong JC, Mandal BK. Adenovirus infections in human immunodeficiency virus-positive patients: clinical features and molecular epidemiology. J Infect Dis 1995;172:629-37.

81. Curlin ME, Huang ML, Lu X, et al. Frequent detection of human adenovirus from the lower gastrointestinal tract in men who have sex with men. PLoS One 2010;5:e11321.

82. Dubberke ER, Tu B, Rivet DJ, et al. Acute meningoencephalitis caused by adenovirus serotype 26. J Neurovirol 2006;12:235-40.

83. Koneru B, Jaffe R, Esquivel CO, et al. Adenoviral infections in pediatric liver transplant recipients. JAMA 1987;258:489-92.

84. Venard V, Carret A, Corsaro D, Bordigoni P, Le Faou A. Genotyping of adenoviruses isolated in an outbreak in a bone marrow transplant unit shows that diverse strains are involved. Journal Hosp Infect 2000;44:71-4.

85. Al Qurashi YM, Guiver M, Cooper RJ. Sequence typing of adenovirus from samples from hematological stem cell transplant recipients. J Med Virol 2011;83:1951-8.

86. Tucker SN, Tingley DW, Scallan CD. Oral adenoviral-based vaccines: historical perspective and future opportunity. Exp Rev Vaccines 2008;7:25-31.

87. Kuschner RA, Russell KL, Abuja M, et al. A phase 3, randomized, double-blind, placebo-controlled study of the safety and efficacy of the live, oral adenovirus type 4 and type 7 vaccine, in U.S. military recruits. Vaccine 2013;31:2963-71.

88. Deal C, Pekosz A, Ketner G. Prospects for oral replicating adenovirus-vectored vaccines. Vaccine 2013;31:3236-43.

89. Schwartz AR, Togo Y, Hornick RB. Clinical evaluation of live, oral types 1, 2, and 5 adenovirus vaccines. Am Rev Respir Dis 1974;109:233-9.

90. Gutekunst RR, White RJ, Edmondson WP, Chanock RM. Immunization with live type 4 adenovirus: determination of infectious virus dose and protective effect of enteric infection. Am J Epidemiol 1967;86:341-9.

91. Lyons A, Longfield J, Kuschner R, et al. A double-blind, placebo-controlled study of the safety and immunogenicity of live, oral type 4 and type 7 adenovirus vaccines in adults. Vaccine 2008;26:2890-8.

92. Stanley ED, Jackson GG. Spread of enteric live adenovirus type 4 vaccine in married couples. J Infect Dis 1969;119:51-9.

93. Chin J, Lennette EH, Schieble JH, Magoffin RL. Concurrent administration of live adenovirus, type 4 and live Poliovirus, type 1 vaccines. Am J Epidemiol 1968;87:633-42.

94. Rosenbaum MJ, De Berry P, Sullivan EJ, et al. Characteristics of vaccine-induced and natural infection with adenovirus type 4 in naval recruits. Am J Epidemiol 1968;88:45-54.

95. Top FH, Jr., Grossman RA, Bartelloni PJ, et al. Immunization with live types 7 and 4 adenovirus vaccines. I. Safety, infectivity, antigenicity, and potency of adenovirus type 7 vaccine in humans. J Infect Dis 1971;124:148-54.

96. Tacket CO, Losonsky G, Lubeck MD, et al. Initial safety and immunogenicity studies of an oral recombinant adenohepatitis B vaccine. Vaccine 1992;10:673-6.

97. Dudding BA, Bartelloni PJ, Scott RM, Top FH, Jr., Russell PK, Buescher EL. Enteric immunization with live adenovirus type 21 vaccine. I. Tests for safety, infectivity, immunogenicity, and potency in volunteers. Infect Immun 1972;5:295-9.

98. Baden LR, Walsh SR, Seaman MS, et al. First-in-human evaluation of the safety and immunogenicity of a recombinant adenovirus serotype 26 HIV-1 Env vaccine (IPCAVD 001). J Infect Dis 2013;207:240-7.

99. Barouch DH, Liu J, Peter L, et al. Characterization of humoral and cellular immune responses elicited by a recombinant adenovirus serotype 26 HIV-1 Env vaccine in healthy adults (IPCAVD 001). J Infect Dis 2013;207:248-56.

17 PROTOCOL SIGNATURE PAGE

The signatures below constitute the approval of this protocol and the appendices and provide the necessary assurances that this study will be conducted in compliance with the protocol, Good Clinical Practices (GCP) and the applicable regulatory requirement(s).

Sponsor:

Signed: ________________________ Date: ____________

Frances Priddy, MD, MPH
Chief Medical Officer, Medical Affairs, IAVI

Protocol Chair:

Signed: ________________________ Date: ____________

Kathryn E. Stephenson, MD, MPH
Beth Israel Deaconess Medical Center

Site Principal Investigator:

Signed: ________________________ Date: ____________

John J. Treanor, MD
University of Rochester Medical Center

18 APPENDICES

18.1 Toxicity Tables

Division of AIDS table for grading the severity of
ADULT AND PEDIATRIC adverse Events
Version 1.0, December, 2004; clarification AUGUST 2009

The Division of AIDS Table for Grading the Severity of Adult and Pediatric Adverse Events (“DAIDS AE Grading Table”) is a descriptive terminology which can be utilized for Adverse Event (AE) reporting. A grading (severity) scale is provided for each AE term.

This clarification of the DAIDS Table for Grading the Severity of Adult and Pediatric AE’s provides additional explanation of the DAIDS AE Grading Table and clarifies some of the parameters.

I. Instructions and Clarifications

Grading Adult and Pediatric AEs
The DAIDS AE Grading Table includes parameters for grading both Adult and Pediatric AEs. When a single set of parameters is not appropriate for grading specific types of AEs for both Adult and Pediatric populations, separate sets of parameters for Adult and/or Pediatric populations (with specified respective age ranges) are given in the Table. If there is no distinction in the Table between Adult and Pediatric values for a type of AE, then the single set of parameters listed is to be used for grading the severity of both Adult and Pediatric events of that type.

Note: In the classification of AEs, the term “severe” is not the same as “serious”. Severity is an indication of the intensity of a specific event (as in mild, moderate, or severe chest pain). The term “serious” relates to a volunteer/event outcome or action criteria, usually associated with events that pose a threat to a volunteer’s life or functioning.

Grade 5
For any AE where the outcome is death, the severity of the AE is classified as Grade 5.

Estimating Severity Grade for Parameters Not Identified in the Table
In order to grade a clinical AE that is not identified in the DAIDS AE grading table, use the category “Estimating Severity Grade”.

Determining Severity Grade for Parameters “Between Grades”
If the severity of a clinical AE could fall under either one of two grades (e.g. the severity of an AE could be either Grade 2 or Grade 3), select the higher of the two grades for the AE. If a laboratory value that is graded as a multiple of the ULN or LLN falls between two grades, select the higher of the two grades for the AE. For example, Grade 1 is 2.5 x ULN and Grade 2 is 2.6 x ULN for a parameter. If the lab value is 2.53 x ULN (which is between the two grades), the severity of this AE would be Grade 2, the higher of the two grades.

Values Below Grade 1
Any laboratory value that is between either the LLN or ULN and Grade 1 should not be graded.

Determining Severity Grade when Local Laboratory Normal Values Overlap with Grade 1 Ranges.
In these situations, the severity grading is based on the ranges in the DAIDS AE Grading Table, even when there is a reference to the local lab LLN.

For example: Phosphate, Serum, Low, Adult and Pediatric >14 years (Page 20) Grade 1 range is 2.50 mg/dL - <LLN. A particular laboratory's normal range for Phosphate is 2.1 – 3.8 mg/dL. A volunteer's actual lab value is 2.5. In this case, the value of 2.5 exceeds the LLN for the local lab, but will be graded as Grade 1 per DAIDS AE Grading Table.

II. Definitions of terms used in the Table:

**Basic Self-care Functions**	Adult<br>Activities such as bathing, dressing, toileting, transfer/movement, continence, and feeding.<br><br>Young Children<br>Activities that are age and culturally appropriate (e.g. feeding self with culturally appropriate eating implement).
**LLN**	Lower limit of normal
**Medical Intervention**	Use of pharmacologic or biologic agent(s) for treatment of an AE.
**NA**	Not Applicable
**Operative Intervention**	Surgical OR other invasive mechanical procedures.
**ULN**	Upper limit of normal
**Usual Social and Functional Activities**	Adult<br>Adaptive tasks and desirable activities, such as going to work, shopping, cooking, use of transportation, pursuing a hobby, etc.<br><br>Young Children<br>Activities that are age and culturally appropriate (e.g. social interactions, play activities, learning tasks, etc.).

PARAMETER	GRADE 1<br>MILD	GRADE 2<br>MODERATE	GRADE 3<br>SEVERE	GRADE 4<br>POTENTIALLY<br>LIFE-THREATENING
**ESTIMATING SEVERITY GRADE**				
Clinical adverse event NOT identified elsewhere in this DAIDS AE Grading Table	Symptoms causing no or minimal interference with usual social & functional activities	Symptoms causing greater than minimal interference with usual social & functional activities	Symptoms causing inability to perform usual social & functional activities	Symptoms causing inability to perform basic self-care functions OR Medical or operative intervention indicated to prevent permanent impairment, persistent disability, or death
**SYSTEMIC**				
Acute systemic allergic reaction	Localized urticaria (wheals) with no medical intervention indicated	Localized urticaria with medical intervention indicated OR Mild angioedema with no medical intervention indicated	Generalized urticaria OR Angioedema with medical intervention indicated OR Symptomatic mild bronchospasm	Acute anaphylaxis OR Life-threatening bronchospasm OR laryngeal edema
Chills	Symptoms causing no or minimal interference with usual social & functional activities	Symptoms causing greater than minimal interference with usual social & functional activities	Symptoms causing inability to perform usual social & functional activities	NA
Fatigue<br>Malaise	Symptoms causing no or minimal interference with usual social & functional activities	Symptoms causing greater than minimal interference with usual social & functional activities	Symptoms causing inability to perform usual social & functional activities	Incapacitating fatigue/ malaise symptoms causing inability to perform basic self-care functions
Fever (nonaxillary)	37.7 – 38.6 ^{o}C	38.7 – 39.3 ^{o}C	39.4 – 40.5 ^{o}C	>40.5 ^{o}C
Pain (indicate body site)<br>DO NOT use for pain due to injection (See Injection Site Reactions: Injection site pain)<br>See also Headache, Arthralgia, and Myalgia	Pain causing no or minimal interference with usual social & functional activities	Pain causing greater than minimal interference with usual social & functional activities	Pain causing inability to perform usual social & functional activities	Disabling pain causing inability to perform basic self-care functions; OR Hospitalization (other than emergency room visit) indicated

PARAMETER	GRADE 1 MILD	GRADE 2 MODERATE	GRADE 3 SEVERE	GRADE 4 POTENTIALLY LIFE-THREATENING
Unintentional weight loss	NA	5 – 9% loss in body weight from baseline	10 – 19% loss in body weight from baseline	≥ 20% loss in body weight from baseline OR Aggressive intervention indicated [e.g. tube feeding or total parenteral nutrition (TPN)]
**INFECTION**				
Infection (any other than HIV infection)	Localized, no systemic antimicrobial treatment indicated AND Symptoms causing no or minimal interference with usual social & functional activities	Systemic antimicrobial treatment indicated OR Symptoms causing greater than minimal interference with usual social & functional activities	Systemic antimicrobial treatment indicated AND Symptoms causing inability to perform usual social & functional activities OR Operative intervention (other than simple incision and drainage) indicated	Life-threatening consequences (e.g. septic shock)
**INJECTION SITE REACTIONS**				
Injection site pain (pain without touching)<br>Or<br>Tenderness (pain when area is touched)	Pain/tenderness causing no or minimal limitation of use of limb	Pain/tenderness limiting use of limb OR Pain/tenderness causing greater than minimal interference with usual social & functional activities	Pain/tenderness causing inability to perform usual social & functional activities	Pain/tenderness causing inability to perform basic self-care function OR Hospitalization (other than emergency room visit) indicated for management of pain/tenderness
Injection site reaction (localized)				
**Adult >15 years**	Erythema OR Induration of 5x5 cm – 9x9 cm (or 25 cm^2 – 81cm^2)	Erythema OR Induration OR Edema >9 cm any diameter (or >81 cm^2)	Ulceration OR Secondary infection OR Phlebitis OR Sterile abscess OR Drainage	Necrosis (involving dermis and deeper tissue)

PARAMETER	GRADE 1<br>MILD	GRADE 2<br>MODERATE	GRADE 3<br>SEVERE	GRADE 4<br>POTENTIALLY<br>LIFE-THREATENING
**Pediatric ≤ 15 years**	Erythema OR Induration OR Edema present but ≤ 2.5 cm diameter	Erythema OR Induration OR Edema >2.5 cm diameter but <50% surface area of the extremity segment (e.g. upper arm/thigh)	Erythema OR Induration OR Edema involving ≥ 50% surface area of the extremity segment (e.g. upper arm/thigh) OR Ulceration OR Secondary infection OR Phlebitis OR Sterile abscess OR Drainage	Necrosis (involving dermis and deeper tissue)
Pruritis associated with injection<br>See also Skin: Pruritis (itching - no skin lesions)	Itching localized to injection site AND Relieved spontaneously or with <48 hours treatment	Itching beyond the injection site but not generalized OR Itching localized to injection site requiring ≥ 48 hours treatment	Generalized itching causing inability to perform usual social & functional activities	NA
**SKIN – DERMATOLOGICAL**				
Alopecia	Thinning detectable by study volunteer (or by caregiver for young children and disabled adults)	Thinning or patchy hair loss detectable by health care provider	Complete hair loss	NA
Cutaneous reaction – rash	Localized macular rash	Diffuse macular, maculopapular, or morbilliform rash OR Target lesions	Diffuse macular, maculopapular, or morbilliform rash with vesicles or limited number of bullae OR Superficial ulcerations of mucous membrane limited to one site	Extensive or generalized bullous lesions OR Stevens-Johnson syndrome OR Ulceration of mucous membrane involving two or more distinct mucosal sites OR Toxic epidermal necrolysis (TEN)
Hyperpigmentation	Slight or localized	Marked or generalized	NA	NA
Hypopigmentation	Slight or localized	Marked or generalized	NA	NA
Pruritis (itching – no skin lesions)<br>(See also Injection Site Reactions: Pruritis associated with injection)	Itching causing no or minimal interference with usual social & functional activities	Itching causing greater than minimal interference with usual social & functional activities	Itching causing inability to perform usual social & functional activities	NA

PARAMETER	GRADE 1<br>MILD	GRADE 2<br>MODERATE	GRADE 3<br>SEVERE	GRADE 4<br>POTENTIALLY<br>LIFE-THREATENING
**CARDIOVASCULAR**				
Cardiac arrhythmia (general)<br>(By ECG or physical exam)	Asymptomatic AND No intervention indicated	Asymptomatic AND Non-urgent medical intervention indicated	Symptomatic, non-life-threatening AND Non-urgent medical intervention indicated	Life-threatening arrhythmia OR Urgent intervention indicated
Cardiac-ischemia/infarction	NA	NA	Symptomatic ischemia (stable angina) OR Testing consistent with ischemia	Unstable angina OR Acute myocardial infarction
Hemorrhage (significant acute blood loss)	NA	Symptomatic AND No transfusion indicated	Symptomatic AND Transfusion of ≤ 2 units packed RBCs (for children ≤ 10 cc/kg) indicated	Life-threatening hypotension OR Transfusion of >2 units packed RBCs (for children >10 cc/kg) indicated
Hypertension				
Adult >17 years<br>(with repeat testing at same visit)	140 – 159 mmHg systolic<br>OR<br>90 – 99 mmHg diastolic	160 – 179 mmHg systolic<br>OR<br>100 – 109 mmHg diastolic	≥180 mmHg systolic<br>OR<br>≥110 mmHg diastolic	Life-threatening consequences (e.g. malignant hypertension) OR Hospitalization indicated (other than emergency room visit)
**Correction**: in Grade 2 to 160 - 179 from >160-179 (systolic) and to ≥ 100 -109 from >100-109 (diastolic) and in Grade 3 to ≥ 180 from >180 (systolic) and to ≥ 110 from >110 (diastolic).				
**Pediatric ≤ 17 years**<br>(with repeat testing at same visit)	NA	91st – 94th percentile adjusted for age, height, and gender (systolic and/or diastolic)	≥95th percentile adjusted for age, height, and gender (systolic and/or diastolic)	Life-threatening consequences (e.g. malignant hypertension) OR Hospitalization indicated (other than emergency room visit)
Hypotension	NA	Symptomatic, corrected with oral fluid replacement	Symptomatic, IV fluids indicated	Shock requiring use of vasopressors or mechanical assistance to maintain blood pressure

PARAMETER	GRADE 1<br>MILD	GRADE 2<br>MODERATE	GRADE 3<br>SEVERE	GRADE 4<br>POTENTIALLY<br>LIFE-THREATENING
Pericardial effusion	Asymptomatic, small effusion requiring no intervention	Asymptomatic, moderate or larger effusion requiring no intervention	Effusion with non-life threatening physiologic consequences OR Effusion with non-urgent intervention indicated	Life-threatening consequences (e.g. tamponade) OR Urgent intervention indicated
Prolonged PR interval				
**Adult >16 years**	PR interval<br>0.21 – 0.25 sec	PR interval<br>>0.25 sec	Type II 2nd degree AV block OR Ventricular pause >3.0 sec	Complete AV block
**Pediatric ≤16 years**	1st degree AV block (PR >normal for age and rate)	Type I 2nd degree AV block	Type II 2nd degree AV block	Complete AV block
Prolonged QTc				
**Adult >16 years**	Asymptomatic, QTc interval 0.45 – 0.47 sec OR Increase interval <0.03 sec above baseline	Asymptomatic, QTc interval 0.48 – 0.49 sec OR Increase in interval 0.03 – 0.05 sec above baseline	Asymptomatic, QTc interval ≥ 0.50 sec OR Increase in interval<br>≥ 0.06 sec above baseline	Life-threatening consequences, e.g. Torsade de pointes or other associated serious ventricular dysrhythmia
**Pediatric ≤16 years**	Asymptomatic, QTc interval 0.450 – 0.464 sec	Asymptomatic, QTc interval 0.465 – 0.479 sec	Asymptomatic, QTc interval ≥ 0.480 sec	Life-threatening consequences, e.g. Torsade de pointes or other associated serious ventricular dysrhythmia
Thrombosis/embolism	NA	Deep vein thrombosis AND No intervention indicated (e.g. anticoagulation, lysis filter, invasive procedure)	Deep vein thrombosis AND Intervention indicated (e.g. anticoagulation, lysis filter, invasive procedure)	Embolic event (e.g. pulmonary embolism, life-threatening thrombus)
Vasovagal episode (associated with a procedure of any kind)	Present without loss of consciousness	Present with transient loss of consciousness	NA	NA
Ventricular dysfunction (congestive heart failure)	NA	Asymptomatic diagnostic finding AND intervention indicated	New onset with symptoms OR Worsening symptomatic congestive heart failure	Life-threatening congestive heart failure

PARAMETER	GRADE 1<br>MILD	GRADE 2<br>MODERATE	GRADE 3<br>SEVERE	GRADE 4<br>POTENTIALLY LIFE-THREATENING
**GASTROINTESTINAL**				
Anorexia	Loss of appetite without decreased oral intake	Loss of appetite associated with decreased oral intake without significant weight loss	Loss of appetite associated with significant weight loss	Life-threatening consequences OR Aggressive intervention indicated [e.g. tube feeding or total parenteral nutrition (TPN)]
**Comment:** Please note that, while the grading scale provided for Unintentional Weight Loss may be used as a guideline when grading anorexia, this is not a requirement and should not be used as a substitute for clinical judgment.				
Ascites	Asymptomatic	Symptomatic AND Intervention indicated (e.g. diuretics or therapeutic paracentesis)	Symptomatic despite intervention	Life-threatening consequences
Cholecystitis	NA	Symptomatic AND Medical intervention indicated	Radiologic, endoscopic, or operative intervention indicated	Life-threatening consequences (e.g. sepsis or perforation)
Constipation	NA	Persistent constipation requiring regular use of dietary modifications, laxatives, or enemas	Obstipation with manual evacuation indicated	Life-threatening consequences (e.g. obstruction)
Diarrhea				
**Adult and Pediatric ≥ 1 year**	Transient or intermittent episodes of unformed stools OR Increase of ≤3 stools over baseline per 24-hour period	Persistent episodes of unformed to watery stools OR Increase of 4 – 6 stools over baseline per 24-hour period	Bloody diarrhea OR Increase of ≥7 stools per 24-hour period OR IV fluid replacement indicated	Life-threatening consequences (e.g. hypotensive shock)
**Pediatric <1 year**	Liquid stools (more unformed than usual) but usual number of stools	Liquid stools with increased number of stools OR Mild dehydration	Liquid stools with moderate dehydration	Liquid stools resulting in severe dehydration with aggressive rehydration indicated OR Hypotensive shock

PARAMETER	GRADE 1<br>MILD	GRADE 2<br>MODERATE	GRADE 3<br>SEVERE	GRADE 4<br>POTENTIALLY<br>LIFE-THREATENING
Dysphagia-Odynophagia	Symptomatic but able to eat usual diet	Symptoms causing altered dietary intake without medical intervention indicated	Symptoms causing severely altered dietary intake with medical intervention indicated	Life-threatening reduction in oral intake
Mucositis/stomatitis (clinical exam)<br>Indicate site (e.g. larynx, oral)<br>See Genitourinary for Vulvovaginitis<br>See also Dysphagia-Odynophagia and Proctitis	Erythema of the mucosa	Patchy pseudomembranes or ulcerations	Confluent pseudomembranes or ulcerations OR Mucosal bleeding with minor trauma	Tissue necrosis OR Diffuse spontaneous mucosal bleeding OR Life-threatening consequences (e.g. aspiration, choking)
Nausea	Transient (<24 hours) or intermittent nausea with no or minimal interference with oral intake	Persistent nausea resulting in decreased oral intake for 24 – 48 hours	Persistent nausea resulting in minimal oral intake for >48 hours OR Aggressive rehydration indicated (e.g. IV fluids)	Life-threatening consequences (e.g. hypotensive shock)
Pancreatitis	NA	Symptomatic AND Hospitalization not indicated (other than emergency room visit)	Symptomatic AND Hospitalization indicated (other than emergency room visit)	Life-threatening consequences (e.g. circulatory failure, hemorrhage, sepsis)
Proctitis (functional-symptomatic)<br>Also see Mucositis/stomatitis for clinical exam	Rectal discomfort AND No intervention indicated	Symptoms causing greater than minimal interference with usual social & functional activities OR Medical intervention indicated	Symptoms causing inability to perform usual social & functional activities OR Operative intervention indicated	Life-threatening consequences (e.g. perforation)
Vomiting	Transient or intermittent vomiting with no or minimal interference with oral intake	Frequent episodes of vomiting with no or mild dehydration	Persistent vomiting resulting in orthostatic hypotension OR Aggressive rehydration indicated (e.g. IV fluids)	Life-threatening consequences (e.g. hypotensive shock)

PARAMETER	GRADE 1<br>MILD	GRADE 2<br>MODERATE	GRADE 3<br>SEVERE	GRADE 4<br>POTENTIALLY<br>LIFE-THREATENING
**NEUROLOGIC**				
Alteration in personality-behavior or in mood (e.g. agitation, anxiety, depression, mania, psychosis)	Alteration causing no or minimal interference with usual social & functional activities	Alteration causing greater than minimal interference with usual social & functional activities	Alteration causing inability to perform usual social & functional activities	Behavior potentially harmful to self or others (e.g. suicidal and homicidal ideation or attempt, acute psychosis) OR Causing inability to perform basic self-care functions
Altered Mental Status<br>For Dementia, see Cognitive and behavioral/attention al disturbance (including dementia and attention deficit disorder)	Changes causing no or minimal interference with usual social & functional activities	Mild lethargy or somnolence causing greater than minimal interference with usual social & functional activities	Confusion, memory impairment, lethargy, or somnolence causing inability to perform usual social & functional activities	Delirium OR obtundation, OR coma
Ataxia	Asymptomatic ataxia detectable on exam OR Minimal ataxia causing no or minimal interference with usual social & functional activities	Symptomatic ataxia causing greater than minimal interference with usual social & functional activities	Symptomatic ataxia causing inability to perform usual social & functional activities	Disabling ataxia causing inability to perform basic self-care functions
Cognitive and behavioral/attention al disturbance (including dementia and attention deficit disorder)	Disability causing no or minimal interference with usual social & functional activities OR Specialized resources not indicated	Disability causing greater than minimal interference with usual social & functional activities OR Specialized resources on part-time basis indicated	Disability causing inability to perform usual social & functional activities OR Specialized resources on a full-time basis indicated	Disability causing inability to perform basic self-care functions OR Institutionalization indicated
CNS ischemia (acute)	NA	NA	Transient ischemic attack	Cerebral vascular accident (CVA, stroke) with neurological deficit

PARAMETER	GRADE 1<br>MILD	GRADE 2<br>MODERATE	GRADE 3<br>SEVERE	GRADE 4<br>POTENTIALLY<br>LIFE-THREATENING
Developmental delay – **Pediatric ≤ 16 years**	Mild developmental delay, either motor or cognitive, as determined by comparison with a developmental screening tool appropriate for the setting	Moderate developmental delay, either motor or cognitive, as determined by comparison with a developmental screening tool appropriate for the setting	Severe developmental delay, either motor or cognitive, as determined by comparison with a developmental screening tool appropriate for the setting	Developmental regression, either motor or cognitive, as determined by comparison with a developmental screening tool appropriate for the setting
Headache	Symptoms causing no or minimal interference with usual social & functional activities	Symptoms causing greater than minimal interference with usual social & functional activities	Symptoms causing inability to perform usual social & functional activities	Symptoms causing inability to perform basic self-care functions OR Hospitalization indicated (other than emergency room visit) OR Headache with significant impairment of alertness or other neurologic function
Insomnia	NA	Difficulty sleeping causing greater than minimal interference with usual social & functional activities	Difficulty sleeping causing inability to perform usual social & functional activities	Disabling insomnia causing inability to perform basic self-care functions
Neuromuscular weakness (including myopathy & neuropathy)	Asymptomatic with decreased strength on exam OR Minimal muscle weakness causing no or minimal interference with usual social & functional activities	Muscle weakness causing greater than minimal interference with usual social & functional activities	Muscle weakness causing inability to perform usual social & functional activities	Disabling muscle weakness causing inability to perform basic self-care functions OR Respiratory muscle weakness impairing ventilation
Neurosensory alteration (including paresthesia and painful neuropathy)	Asymptomatic with sensory alteration on exam or minimal paresthesia causing no or minimal interference with usual social & functional activities	Sensory alteration or paresthesia causing greater than minimal interference with usual social & functional activities	Sensory alteration or paresthesia causing inability to perform usual social & functional activities	Disabling sensory alteration or paresthesia causing inability to perform basic self-care functions

PARAMETER	GRADE 1<br>MILD	GRADE 2<br>MODERATE	GRADE 3<br>SEVERE	GRADE 4<br>POTENTIALLY<br>LIFE-THREATENING
Seizure: (new onset) **– Adult ≥18 years**<br>See also Seizure: (known pre-existing seizure disorder)	NA	1 seizure	2 – 4 seizures	Seizures of any kind which are prolonged, repetitive (e.g. status epilepticus), or difficult to control (e.g. refractory epilepsy)
Seizure: (known pre-existing seizure disorder)<br>**– Adult ≥18 years**<br>For worsening of existing epilepsy the grades should be based on an increase from previous level of control to any of these levels.	NA	Increased frequency of pre-existing seizures (non-repetitive) without change in seizure character OR Infrequent break-through seizures while on stable medication in a previously controlled seizure disorder	Change in seizure character from baseline either in duration or quality (e.g. severity or focality)	Seizures of any kind which are prolonged, repetitive (e.g. status epilepticus), or difficult to control (e.g. refractory epilepsy)
Seizure<br>**– Pediatric <18 years**	Seizure, generalized onset with or without secondary generalization, lasting <5 minutes with <24 hours post-ictal state	Seizure, generalized onset with or without secondary generalization, lasting 5 – 20 minutes with <24 hours post-ictal state	Seizure, generalized onset with or without secondary generalization, lasting >20 minutes	Seizure, generalized onset with or without secondary generalization, requiring intubation and sedation
Syncope (not associated with a procedure)	NA	Present	NA	NA
Vertigo	Vertigo causing no or minimal interference with usual social & functional activities	Vertigo causing greater than minimal interference with usual social & functional activities	Vertigo causing inability to perform usual social & functional activities	Disabling vertigo causing inability to perform basic self-care functions
**RESPIRATORY**				
Bronchospasm (acute)	FEV1 or peak flow reduced to 70 – 80%	FEV1 or peak flow 50 – 69%	FEV1 or peak flow 25 – 49%	Cyanosis OR FEV1 or peak flow <25% OR Intubation
Dyspnea or respiratory distress				

PARAMETER	GRADE 1 MILD	GRADE 2 MODERATE	GRADE 3 SEVERE	GRADE 4 POTENTIALLY LIFE-THREATENING
**Adult ≥14 years**	Dyspnea on exertion with no or minimal interference with usual social & functional activities	Dyspnea on exertion causing greater than minimal interference with usual social & functional activities	Dyspnea at rest causing inability to perform usual social & functional activities	Respiratory failure with ventilatory support indicated
**Pediatric <14 years**	Wheezing OR minimal increase in respiratory rate for age	Nasal flaring OR Intercostal retractions OR Pulse oximetry 90 – 95%	Dyspnea at rest causing inability to perform usual social & functional activities OR Pulse oximetry <90%	Respiratory failure with ventilatory support indicated
**MUSCULOSKELETAL**				
Arthralgia<br>See also Arthritis	Joint pain causing no or minimal interference with usual social & functional activities	Joint pain causing greater than minimal interference with usual social & functional activities	Joint pain causing inability to perform usual social & functional activities	Disabling joint pain causing inability to perform basic self-care functions
Arthritis<br>See also Arthralgia	Stiffness or joint swelling causing no or minimal interference with usual social & functional activities	Stiffness or joint swelling causing greater than minimal interference with usual social & functional activities	Stiffness or joint swelling causing inability to perform usual social & functional activities	Disabling joint stiffness or swelling causing inability to perform basic self-care functions
Bone Mineral Loss				
**Adult ≥21 years**	BMD t-score -2.5 to -1.0	BMD t-score <-2.5	Pathological fracture (including loss of vertebral height)	Pathologic fracture causing life-threatening consequences
**Pediatric <21 years**	BMD z-score -2.5 to -1.0	BMD z-score <-2.5	Pathological fracture (including loss of vertebral height)	Pathologic fracture causing life-threatening consequences
Myalgia (non-injection site)	Muscle pain causing no or minimal interference with usual social & functional activities	Muscle pain causing greater than minimal interference with usual social & functional activities	Muscle pain causing inability to perform usual social & functional activities	Disabling muscle pain causing inability to perform basic self-care functions

PARAMETER	GRADE 1<br>MILD	GRADE 2<br>MODERATE	GRADE 3<br>SEVERE	GRADE 4<br>POTENTIALLY<br>LIFE-THREATENING
Osteonecrosis	NA	Asymptomatic with radiographic findings AND No operative intervention indicated	Symptomatic bone pain with radiographic findings OR Operative intervention indicated	Disabling bone pain with radiographic findings causing inability to perform basic self-care functions
**GENITOURINARY**				
Cervicitis (<u>symptoms</u>)<br>(For use in studies evaluating topical study agents)<br>For other cervicitis see Infection: Infection (any other than HIV infection)	Symptoms causing no or minimal interference with usual social & functional activities	Symptoms causing greater than minimal interference with usual social & functional activities	Symptoms causing inability to perform usual social & functional activities	Symptoms causing inability to perform basic self-care functions
Cervicitis (<u>clinical exam</u>)<br>(For use in studies evaluating topical study agents)<br>For other cervicitis see Infection: Infection (any other than HIV infection)	Minimal cervical abnormalities on examination (erythema, mucopurulent discharge, or friability) OR Epithelial disruption <25% of total surface	Moderate cervical abnormalities on examination (erythema, mucopurulent discharge, or friability) OR Epithelial disruption of 25 – 49% total surface	Severe cervical abnormalities on examination (erythema, mucopurulent discharge, or friability) OR Epithelial disruption 50 – 75% total surface	Epithelial disruption >75% total surface
Inter-menstrual bleeding (IMB)	Spotting observed by volunteer OR Minimal blood observed during clinical or colposcopic examination	Inter-menstrual bleeding not greater in duration or amount than usual menstrual cycle	Inter-menstrual bleeding greater in duration or amount than usual menstrual cycle	Hemorrhage with life-threatening hypotension OR Operative intervention indicated
Urinary tract obstruction (e.g. stone)	NA	Signs or symptoms of urinary tract obstruction without hydronephrosis or renal dysfunction	Signs or symptoms of urinary tract obstruction with hydronephrosis or renal dysfunction	Obstruction causing life-threatening consequences

PARAMETER	GRADE 1<br>MILD	GRADE 2<br>MODERATE	GRADE 3<br>SEVERE	GRADE 4<br>POTENTIALLY<br>LIFE-THREATENING
Vulvovaginitis (symptoms)<br>(Use in studies evaluating topical study agents)<br>For other vulvovaginitis see Infection: Infection (any other than HIV infection)	Symptoms causing no or minimal interference with usual social & functional activities	Symptoms causing greater than minimal interference with usual social & functional activities	Symptoms causing inability to perform usual social & functional activities	Symptoms causing inability to perform basic self-care functions
Vulvovaginitis (clinical exam)<br>(Use in studies evaluating topical study agents)<br>For other vulvovaginitis see Infection: Infection (any other than HIV infection)	Minimal vaginal abnormalities on examination OR Epithelial disruption <25% of total surface	Moderate vaginal abnormalities on examination OR Epithelial disruption of 25 - 49% total surface	Severe vaginal abnormalities on examination OR Epithelial disruption 50 - 75% total surface	Vaginal perforation OR Epithelial disruption >75% total surface
**OCULAR/VISUAL**				
Uveitis	Asymptomatic but detectable on exam	Symptomatic anterior uveitis OR Medical intervention indicated	Posterior or pan-uveitis OR Operative intervention indicated	Disabling visual loss in affected eye(s)
Visual changes (from baseline)	Visual changes causing no or minimal interference with usual social & functional activities	Visual changes causing greater than minimal interference with usual social & functional activities	Visual changes causing inability to perform usual social & functional activities	Disabling visual loss in affected eye(s)
**ENDOCRINE/METABOLIC**				
Abnormal fat accumulation (e.g. back of neck, breasts, abdomen)	Detectable by study volunteer (or by caregiver for young children and disabled adults)	Detectable on physical exam by health care provider	Disfiguring OR Obvious changes on casual visual inspection	NA
Diabetes mellitus	NA	New onset without need to initiate medication OR Modification of current medications to regain glucose control	New onset with initiation of medication indicated OR Diabetes uncontrolled despite treatment modification	Life-threatening consequences (e.g. ketoacidosis, hyperosmolar non-ketotic coma)

PARAMETER	GRADE 1 MILD	GRADE 2 MODERATE	GRADE 3 SEVERE	GRADE 4 POTENTIALLY LIFE-THREATENING
Gynecomastia	Detectable by study volunteer or caregiver (for young children and disabled adults)	Detectable on physical exam by health care provider	Disfiguring OR Obvious on casual visual inspection	NA
Hyperthyroidism	Asymptomatic	Symptomatic causing greater than minimal interference with usual social & functional activities OR Thyroid suppression therapy indicated	Symptoms causing inability to perform usual social & functional activities OR Uncontrolled despite treatment modification	Life-threatening consequences (e.g. thyroid storm)
Hypothyroidism	Asymptomatic	Symptomatic causing greater than minimal interference with usual social & functional activities OR Thyroid replacement therapy indicated	Symptoms causing inability to perform usual social & functional activities OR Uncontrolled despite treatment modification	Life-threatening consequences (e.g. myxedema coma)
Lipoatrophy (e.g. fat loss from the face, extremities, buttocks)	Detectable by study volunteer (or by caregiver for young children and disabled adults)	Detectable on physical exam by health care provider	Disfiguring OR Obvious on casual visual inspection	NA

LABORATORY				
**PARAMETER**	**GRADE 1<br>MILD**	**GRADE 2<br>MODERATE**	**GRADE 3<br>SEVERE**	**GRADE 4<br>POTENTIALLY LIFE-THREATENING**
**HEMATOLOGY** ***Standard International Units are listed in italics***				
Absolute CD4^{+} count<br>**– Adult and Pediatric >13 years**<br>(HIV NEGATIVE ONLY)	300 – 400/mm^3<br>*300 – 400/µL*	200 – 299/mm^3<br>*200 – 299/µL*	100 – 199/mm^3<br>*100 – 199/µL*	<100/mm^3<br>*<100/µL*
Absolute lymphocyte count<br>**– Adult and Pediatric >13 years**<br>(HIV NEGATIVE ONLY)	600 – 650/mm^3<br>*0.600 x 10^9 – 0.650 x 10^9/L*	500 – 599/mm^3<br>*0.500 x 10^9 – 0.599 x 10^9/L*	350 – 499/mm^3<br>*0.350 x 10^9 – 0.499 x 10^9/L*	<350/mm^3<br>*<0.350 x 10^9/L*
**Comment:** Values in children ≤13 years are not given for the two parameters above because the absolute counts are variable.				
Absolute neutrophil count (ANC)				
**Adult and Pediatric, >7 days**	1,000 – 1,300/mm^3<br>*1.000 x 10^9 – 1.300 x 10^9/L*	750 – 999/mm^3<br>*0.750 x 10^9 – 0.999 x 10^9/L*	500 – 749/mm^3<br>*0.500 x 10^9 – 0.749 x 10^9/L*	<500/mm^3<br>*<0.500 x 10^9/L*
**Infant*†, 2 – ≤ 7 days**	1,250 – 1,500/mm^3<br>*1.250 x 10^9 – 1.500 x 10^9/L*	1,000 – 1,249/mm^3<br>*1.000 x 10^9 – 1.249 x 10^9/L*	750 – 999/mm^3<br>*0.750 x 10^9 – 0.999 x 10^9/L*	<750/mm^3<br>*<0.750 x 10^9/L*
**Infant*†, ≤1 day**	4,000 – 5,000/mm^3<br>*4.000 x 10^9 – 5.000 x 10^9/L*	3,000 – 3,999/mm^3<br>*3.000 x 10^9 – 3.999 x 10^9/L*	1,500 – 2,999/mm^3<br>*1.500 x 10^9 – 2.999 x 10^9/L*	<1,500/mm^3<br>*<1.500 x 10^9/L*
**Comment:** Parameter changed from "Infant, <1 day" to "Infant, ≤1 day"				
Fibrinogen, decreased	100 – 200 mg/dL<br>*1.00 – 2.00 g/L*<br>OR<br>0.75 – 0.99 x LLN	75 – 99 mg/dL<br>*0.75 – 0.99 g/L*<br>OR<br>0.50 – 0.74 x LLN	50 – 74 mg/dL<br>*0.50 – 0.74 g/L*<br>OR<br>0.25 – 0.49 x LLN	<50 mg/dL<br>*<0.50 g/L*<br>OR<br><0.25 x LLN<br>OR<br>Associated with gross bleeding
Hemoglobin (Hgb)				
Comment: The Hgb values in mmol/L have changed because the conversion factor used to convert g/dL to mmol/L has been changed from 0.155 to 0.6206 (the most commonly used conversion factor). For grading Hgb results obtained by an analytic method with a conversion factor other than 0.6206, the result must be converted to g/dL using the appropriate conversion factor for that lab.				
**Adult and Pediatric ≥ 57 days**<br>(HIV POSITIVE ONLY)	8.5 – 10.0 g/dL<br>*5.24 – 6.23 mmol/L*	7.5 – 8.4 g/dL<br>*4.62–5.23 mmol/L*	6.50 – 7.4 g/dL<br>*4.03–4.61 mmol/L*	<6.5 g/dL<br>*<4.03 mmol/L*
**Adult and Pediatric ≥ 57 days**<br>(HIV NEGATIVE ONLY)	10.0 – 10.9 g/dL<br>*6.18 – 6.79 mmol/L*<br>OR<br>Any decrease<br>2.5 – 3.4 g/dL<br>*1.58 – 2.13 mmol/L*	9.0 – 9.9 g/dL<br>*5.55 - 6.17 mmol/L*<br>OR<br>Any decrease<br>3.5 – 4.4 g/dL<br>*2.14 – 2.78 mmol/L*	7.0 – 8.9 g/dL<br>*4.34 - 5.54 mmol/L*<br>OR<br>Any decrease<br>≥ 4.5 g/dL<br>*>2.79 mmol/L*	<7.0 g/dL<br>*<4.34 mmol/L*
**Comment:** The decrease is a decrease from baseline				

LABORATORY				
**PARAMETER**	**GRADE 1 MILD**	**GRADE 2 MODERATE**	**GRADE 3 SEVERE**	**GRADE 4 POTENTIALLY LIFE-THREATENING**
**Infant*†, 36 – 56 days** (HIV POSITIVE OR NEGATIVE)	8.5 – 9.4 g/dL *5.24 – 5.86 mmol/L*	7.0 – 8.4 g/dL *4.31 – 5.23 mmol/L*	6.0 – 6.9 g/dL *3.72 – 4.30 mmol/L*	<6.00 g/dL *<3.72 mmol/L*
**Infant*†, 22 – 35 days** (HIV POSITIVE OR NEGATIVE)	9.5 – 10.5 g/dL *5.87 - 6.54 mmol/L*	8.0 – 9.4 g/dL *4.93 – 5.86 mmol/L*	7.0 – 7.9 g/dL *4.34 – 4.92 mmol/L*	<7.00 g/dL *<4.34 mmol/L*
**Infant*†, ≤21 days** (HIV POSITIVE OR NEGATIVE)	12.0 – 13.0 g/dL *7.42 – 8.09 mmol/L*	10.0 – 11.9 g/dL *6.18 – 7.41 mmol/L*	9.0 – 9.9 g/dL *5.59- 6.17 mmol/L*	<9.0 g/dL *<5.59 mmol/L*
**Correction**: Parameter changed from “Infant <21 days” to “Infant ≤21 days”				
International Normalized Ratio of prothrombin time (INR)	1.1 – 1.5 x ULN	1.6 – 2.0 x ULN	2.1 – 3.0 x ULN	>3.0 x ULN
Methemoglobin	5.0 – 10.0%	10.1 – 15.0%	15.1 – 20.0%	>20.0%
Prothrombin Time (PT)	1.1 – 1.25 x ULN	1.26 – 1.50 x ULN	1.51 – 3.00 x ULN	>3.00 x ULN
Partial Thromboplastin Time (PTT)	1.1 – 1.66 x ULN	1.67 – 2.33 x ULN	2.34 – 3.00 x ULN	>3.00 x ULN
Platelets, decreased	100,000 – 124,999/mm^3 *100.000 x 10^9 – 124.999 x 10^9/L*	50,000 – 99,999/mm^3 *50.000 x 10^9 – 99.999 x 10^9/L*	25,000 – 49,999/mm^3 *25.000 x 10^9 – 49.999 x 10^9/L*	<25,000/mm^3 *<25.000 x 10^9/L*
WBC, decreased	2,000 – 2,500/mm^3 *2.000 x 10^9 – 2.500 x 10^9/L*	1,500 – 1,999/mm^3 *1.500 x 10^9 – 1.999 x 10^9/L*	1,000 – 1,499/mm^3 *1.000 x 10^9 – 1.499 x 10^9/L*	<1,000/mm^3 *<1.000 x 10^9/L*
**CHEMISTRIES**	***Standard International Units are listed in italics***			
Acidosis	NA	pH <normal, but ≥ 7.3	pH <7.3 without life-threatening consequences	pH <7.3 with life-threatening consequences
Albumin, serum, low	3.0 g/dL – <LLN *30 g/L – <LLN*	2.0 – 2.9 g/dL *20 – 29 g/L*	<2.0 g/dL *<20 g/L*	NA
Alkaline Phosphatase	1.25 – 2.5 x ULN†	2.6 – 5.0 x ULN†	5.1 – 10.0 x ULN†	>10.0 x ULN†
Alkalosis	NA	pH >normal, but ≤ 7.5	pH >7.5 without life-threatening consequences	pH >7.5 with life-threatening consequences
ALT (SGPT)	1.25 – 2.5 x ULN	2.6 – 5.0 x ULN	5.1 – 10.0 x ULN	>10.0 x ULN
AST (SGOT)	1.25 – 2.5 x ULN	2.6 – 5.0 x ULN	5.1 – 10.0 x ULN	>10.0 x ULN
Bicarbonate, serum, low	16.0 mEq/L – <LLN *16.0 mmol/L – <LLN*	11.0 – 15.9 mEq/L *11.0 – 15.9 mmol/L*	8.0 – 10.9 mEq/L *8.0 – 10.9 mmol/L*	<8.0 mEq/L *<8.0 mmol/L*
**Comment:** Some laboratories will report this value as Bicarbonate (HCO_3) and others as Total Carbon Dioxide (CO_2). These are the same tests; values should be graded according to the ranges for Bicarbonate as listed above.				

LABORATORY				
**PARAMETER**	**GRADE 1 MILD**	**GRADE 2 MODERATE**	**GRADE 3 SEVERE**	**GRADE 4 POTENTIALLY LIFE-THREATENING**
Bilirubin (Total)				
**Adult and Pediatric >14 days**	1.1 – 1.5 x ULN	1.6 – 2.5 x ULN	2.6 – 5.0 x ULN	>5.0 x ULN
**Infant*†, ≤14 days** (non-hemolytic)	NA	20.0 – 25.0 mg/dL<br>*342 – 428 µmol/L*	25.1 – 30.0 mg/dL<br>*429 – 513 µmol/L*	>30.0 mg/dL<br>*>513.0 µmol/L*
**Infant*†, ≤14 days** (hemolytic)	NA	NA	20.0 – 25.0 mg/dL<br>*342 – 428 µmol/L*	>25.0 mg/dL<br>*>428 µmol/L*
Calcium, serum, high				
**Adult and Pediatric ≥7 days**	10.6 – 11.5 mg/dL<br>*2.65 – 2.88 mmol/L*	11.6 – 12.5 mg/dL<br>*2.89 – 3.13 mmol/L*	12.6 – 13.5 mg/dL<br>*3.14 – 3.38 mmol/L*	>13.5 mg/dL<br>*>3.38 mmol/L*
**Infant*†, <7 days**	11.5 – 12.4 mg/dL<br>*2.88 – 3.10 mmol/L*	12.5 – 12.9 mg/dL<br>*3.11 – 3.23 mmol/L*	13.0 – 13.5 mg/dL<br>*3.245 – 3.38 mmol/L*	>13.5 mg/dL<br>*>3.38 mmol/L*
Calcium, serum, low				
**Adult and Pediatric ≥7 days**	7.8 – 8.4 mg/dL<br>*1.95 – 2.10 mmol/L*	7.0 – 7.7 mg/dL<br>*1.75 – 1.94 mmol/L*	6.1 – 6.9 mg/dL<br>*1.53 – 1.74 mmol/L*	<6.1 mg/dL<br>*<1.53 mmol/L*
**Infant*†, <7 days**	6.5 – 7.5 mg/dL<br>*1.63 – 1.88 mmol/L*	6.0 – 6.4 mg/dL<br>*1.50 – 1.62 mmol/L*	5.50 – 5.90 mg/dL<br>*1.38 – 1.51 mmol/L*	<5.50 mg/dL<br>*<1.38 mmol/L*
**Comment:** Do not adjust Calcium, serum, low or Calcium, serum, high for albumin				
Cardiac troponin I (cTnI)	NA	NA	NA	Levels consistent with myocardial infarction or unstable angina as defined by the manufacturer
Cardiac troponin T (cTnT)	NA	NA	NA	≥ 0.20 ng/mL<br>OR<br>Levels consistent with myocardial infarction or unstable angina as defined by the manufacturer
Cholesterol (fasting)				
**Adult ≥18 years**	200 – 239 mg/dL<br>*5.18 – 6.19 mmol/L*	240 – 300 mg/dL<br>*6.20 – 7.77 mmol/L*	>300 mg/dL<br>*>7.77 mmol/L*	NA
**Pediatric <18 years**	170 – 199 mg/dL<br>*4.40 – 5.15 mmol/L*	200 – 300 mg/dL<br>*5.16 – 7.77 mmol/L*	>300 mg/dL<br>*>7.77 mmol/L*	NA
Creatine Kinase	3.0 – 5.9 x ULN†	6.0 – 9.9 x ULN†	10.0 – 19.9 x ULN†	≥ 20.0 x ULN†
Creatinine	1.1 – 1.3 x ULN†	1.4 – 1.8 x ULN†	1.9 – 3.4 x ULN†	≥ 3.5 x ULN†
Glucose, serum, high				
Nonfasting	116 – 160 mg/dL<br>*6.44 – 8.88 mmol/L*	161 – 250 mg/dL<br>*8.89 – 13.88 mmol/L*	251 – 500 mg/dL<br>*13.89 – 27.75 mmol/L*	>500 mg/dL<br>*>27.75 mmol/L*

LABORATORY				
**PARAMETER**	**GRADE 1 MILD**	**GRADE 2 MODERATE**	**GRADE 3 SEVERE**	**GRADE 4 POTENTIALLY LIFE-THREATENING**
Fasting	110 – 125 mg/dL<br>*6.11 – 6.94 mmol/L*	126 – 250 mg/dL<br>*6.95 – 13.88 mmol/L*	251 – 500 mg/dL<br>*13.89 – 27.75 mmol/L*	>500 mg/dL<br>*>27.75 mmol/L*
Glucose, serum, low				
**Adult and Pediatric ≥1 month**	55 – 64 mg/dL<br>*3.05 – 3.55 mmol/L*	40 – 54 mg/dL<br>*2.22 – 3.06 mmol/L*	30 – 39 mg/dL<br>*1.67 – 2.23 mmol/L*	<30 mg/dL<br>*<1.67 mmol/L*
**Infant*†, <1 month**	50 – 54 mg/dL<br>*2.78 – 3.00 mmol/L*	40 – 49 mg/dL<br>*2.22 – 2.77 mmol/L*	30 – 39 mg/dL<br>*1.67 – 2.21 mmol/L*	<30 mg/dL<br>*<1.67 mmol/L*
Lactate	ULN - <2.0 x ULN without acidosis	≥ 2.0 x ULN without acidosis	Increased lactate with pH <7.3 without life-threatening consequences	Increased lactate with pH <7.3 with life-threatening consequences
**Comment:** Added ULN to Grade 1 parameter				
LDL cholesterol (fasting)				
**Adult ≥18 years**	130 – 159 mg/dL<br>*3.37 – 4.12 mmol/L*	160 – 190 mg/dL<br>*4.13 – 4.90 mmol/L*	≥ 190 mg/dL<br>*≥ 4.91 mmol/L*	NA
**Pediatric >2 - <18 years**	110 – 129 mg/dL<br>*2.85 – 3.34 mmol/L*	130 – 189 mg/dL<br>*3.35 – 4.90 mmol/L*	≥190 mg/dL<br>*≥4.91 mmol/L*	NA
Lipase	1.1 – 1.5 x ULN	1.6 – 3.0 x ULN	3.1 – 5.0 x ULN	>5.0 x ULN
Magnesium, serum, low	1.2 – 1.4 mEq/L<br>*0.60 – 0.70 mmol/L*	0.9 – 1.1 mEq/L<br>*0.45 – 0.59 mmol/L*	0.6 – 0.8 mEq/L<br>*0.30 – 0.44 mmol/L*	<0.60 mEq/L<br>*<0.30 mmol/L*
Pancreatic amylase	1.1 – 1.5 x ULN	1.6 – 2.0 x ULN	2.1 – 5.0 x ULN	>5.0 x ULN
Phosphate, serum, low				
**Adult and Pediatric >14 years**	2.5 mg/dL – <LLN<br>*0.81 mmol/L – <LLN*	2.0 – 2.4 mg/dL<br>*0.65 – 0.80 mmol/L*	1.0 – 1.9 mg/dL<br>*0.32 – 0.64 mmol/L*	<1.00 mg/dL<br>*<0.32 mmol/L*
**Pediatric 1 year – 14 years**	3.0 – 3.5 mg/dL<br>*0.97 – 1.13 mmol/L*	2.5 – 2.9 mg/dL<br>*0.81 – 0.96 mmol/L*	1.5 – 2.4 mg/dL<br>*0.48 – 0.80 mmol/L*	<1.50 mg/dL<br>*<0.48 mmol/L*
**Pediatric <1 year**	3.5 – 4.5 mg/dL<br>*1.13 – 1.45 mmol/L*	2.5 – 3.4 mg/dL<br>*0.81 – 1.12 mmol/L*	1.5 – 2.4 mg/dL<br>*0.48 – 0.80 mmol/L*	<1.50 mg/dL<br>*<0.48 mmol/L*
Potassium, serum, high	5.6 – 6.0 mEq/L<br>*5.6 – 6.0 mmol/L*	6.1 – 6.5 mEq/L<br>*6.1 – 6.5 mmol/L*	6.6 – 7.0 mEq/L<br>*6.6 – 7.0 mmol/L*	>7.0 mEq/L<br>*>7.0 mmol/L*
Potassium, serum, low	3.0 – 3.4 mEq/L<br>*3.0 – 3.4 mmol/L*	2.5 – 2.9 mEq/L<br>*2.5 – 2.9 mmol/L*	2.0 – 2.4 mEq/L<br>*2.0 – 2.4 mmol/L*	<2.0 mEq/L<br>*<2.0 mmol/L*
Sodium, serum, high	146 – 150 mEq/L<br>*146 – 150 mmol/L*	151 – 154 mEq/L<br>*151 – 154 mmol/L*	155 – 159 mEq/L<br>*155 – 159 mmol/L*	≥ 160 mEq/L<br>*≥ 160 mmol/L*
Sodium, serum, low	130 – 135 mEq/L<br>*130 – 135 mmol/L*	125 – 129 mEq/L<br>*125 – 129 mmol/L*	121 – 124 mEq/L<br>*121 – 124 mmol/L*	≤ 120 mEq/L<br>*≤ 120 mmol/L*
Triglycerides (fasting)	NA	500 – 750 mg/dL<br>*5.65 – 8.48 mmol/L*	751 – 1,200 mg/dL<br>*8.49 – 13.56 mmol/L*	>1,200 mg/dL<br>*>13.56 mmol/L*
Uric acid	7.5 – 10.0 mg/dL<br>*0.45 – 0.59 mmol/L*	10.1 – 12.0 mg/dL<br>*0.60 – 0.71 mmol/L*	12.1 – 15.0 mg/dL<br>*0.72 – 0.89 mmol/L*	>15.0 mg/dL<br>*>0.89 mmol/L*

LABORATORY				
**PARAMETER**	**GRADE 1 MILD**	**GRADE 2 MODERATE**	**GRADE 3 SEVERE**	**GRADE 4 POTENTIALLY LIFE-THREATENING**
**URINALYSIS**	***Standard International Units are listed in italics***			
Hematuria (microscopic)	6 – 10 RBC/HPF	>10 RBC/HPF	Gross, with or without clots OR with RBC casts	Transfusion indicated
Proteinuria, random collection	1 +	2 – 3 +	4 +	NA
Proteinuria, 24 hour collection				
**Adult and Pediatric ≥ 10 years**	200 – 999 mg/24 h<br>*0.200 – 0.999 g/d*	1,000 – 1,999 mg/24 h<br>*1.000 – 1.999 g/d*	2,000 – 3,500 mg/24 h<br>*2.000 – 3.500 g/d*	>3,500 mg/24 h<br>*>3.500 g/d*
**Pediatric >3 mo - <10 years**	201 – 499 mg/m^2/24 h<br>*0.201 – 0.499 g/d*	500 – 799 mg/m^2/24 h<br>*0.500 – 0.799 g/d*	800 – 1,000 mg/m^2/24 h<br>*0.800 – 1.000 g/d*	>1,000 mg/ m^2/24 h<br>*>1.000 g/d*

18.2 HIV Risk Assessment

ASSESSMENT OF SEXUAL BEHAVIORS
(for individuals considered low-risk for HIV transmission)

Consider whether a volunteer would be appropriate for inclusion if the person:

In the last 6 months did not:

- Have vaginal or anal intercourse with an HIV-infected partner, or a partner who uses injection drugs
- Give or receive money, drugs, gifts or services in exchange for sex

AND

In the last 6 months has abstained from penile/anal or penile/vaginal intercourse, OR

In the last 6 months:

- Had 4 or fewer partners of the opposite sex for vaginal and/or anal intercourse, OR

Is an MSM (person born male with partner(s) born male) who, in the last 6 months:

- Had 2 or fewer MSM partners for anal intercourse, AND
- Had no unprotected anal sex with MSM, OR unprotected anal sex with MSM only within a monogamous relationship lasting at least 6 months (during which neither partner had any other partners)

AND

Uses or intends to use condoms in situations of increased risk, which may include penile/anal or penile/vaginal intercourse with new partners of unknown HIV status, occasional partners, partners outside a primary relationship, and/or partners known to have other partners.

ASSESSMENT OF NON-SEXUAL BEHAVIORS

AND, *consider whether a volunteer would be appropriate for inclusion if the person:*

In the last 12 months did not:

- Inject drugs or other substances without a prescription
- Repeatedly use cocaine or methamphetamine
- Repeatedly use excessive alcohol, which in the investigator’s judgment, rendered the volunteer at greater than low risk for acquiring HIV infection.

Exclude a volunteer if:

Within the 12 months prior to enrollment: a history of newly acquired syphilis, gonorrhea, non-gonococcal urethritis, herpes simplex virus type 2 (HSV2),Chlamydia, pelvic inflammatory disease (PID), trichomonas, mucopurulent cervicitis, epididymitis, proctitis, lymphogranuloma venereum, chancroid, or hepatitis B.

18.3 Measures to Prevent Transmission of Vaccine to Staff

Barrier precautions, immunizations, and antiviral strategies will be used to reduce the risk of transmission of vaccine viruses to study personnel, or from personnel to study volunteers. These strategies will include (but not necessarily be limited to) the following policies:

- Study volunteers will remain in single room isolation for the duration of the inpatient portion of the study.
- Contact isolation (gowns and gloves) will be practiced for all contact between staff and study volunteers from the time the vaccine is administered until the day of discharge.
- Study staff will not work in inpatient care settings for at least 24 hours after working a shift on the inpatient Isolation Unit from the time vaccine is administered until the day of discharge.
- Study staff with household members with significant immunocompromising illnesses will not be allowed to have direct contact with study volunteers during the inpatient portion of the study.
- Staff that had contact with potentially infected volunteers and develop a compatible respiratory, gastrointestinal, or febrile illness will be tested by nasal/throat swab real-time (rt)-PCR for infection with the vaccine virus. Staff with such illnesses will remain at home or on the Isolation Unit pending results of the nasal wash or swab testing.
- Study staff must agree to adhere to these policies in order to work on the study during the inpatient phase.

18.4 Key Study Roles

Protocol Chair:

Kathryn Stephenson, MD, MPH
Center for Virology and Vaccine Research
Beth Israel Deaconess Medical Center
330 Brookline Ave, CLS 1046
Boston, MA 02215
Phone: (617) 735-4556
Email: kstephen@bidmc.harvard.edu

Protocol Co-Chairs:

Lindsey Baden, MD
Division of Infectious Diseases
Brigham and Women's Hospital
75 Francis Street, PBB-A4
Boston, MA 02115
Phone: (617) 732-6801
Email: lbaden@partners.org

Yehuda Cohen, MD
Center for Virology and Vaccine Research
Beth Israel Deaconess Medical Center
330 Brookline Ave, CLS-1003, Boston, MA 02215
Phone: (617) 735-4424
Email: ycohen@bidmc.harvard.edu

Sponsor Representative:

Fran Priddy, MD, MPH
Chief Medical Officer
International AIDS Vaccine Initiative
125 Broad Street, 9th Floor
New York, NY 10004
Phone: (212) 847-1111
fpriddy@iavi.org

Site Principal Investigator

John J. Treanor, MD
Division of Infectious Diseases
University of Rochester Medical Center
601 Elmwood Avenue, Box 689
Rochester, NY 14642
Phone: (585) 442-9328
Email: john_treanor@urmc.rochester.edu

Protocol Immunologists

Kathryn Stephenson, MD, MPH
Center for Virology and Vaccine Research
Beth Israel Deaconess Medical Center
330 Brookline Ave, CLS 1046
Boston, MA 02215
Phone: (617) 735-4556
Email: kstephen@bidmc.harvard.edu

Dan Barouch, MD, PhD
Center for Virology and Vaccine Research
Beth Israel Deaconess Medical Center
330 Brookline Ave, CLS 1043
Boston, MA 02215
Phone: (617) 735-4485
Email: dbarouch@bidmc.harvard.edu

Protocol Statistician

Len Dally
The EMMES Corporation
401 N. Washington Street, Suite 700
Rockville, MD 20850
ldally@emmes.com

Laboratory Investigators

Dan Barouch, MD, PhD
Center for Virology and Vaccine Research
Beth Israel Deaconess Medical Center
330 Brookline Ave, CLS 1043
Boston, MA 02215
Phone: (617) 735-4485
Email: dbarouch@bidmc.harvard.edu

Protocol Data Management

The EMMES Corporation
401 N. Washington Street, Suite 700
Rockville, MD 20850
iavitrials@emmes.com

18.5 Schedule Of Procedures - Volunteers

Study Month				0													1	4	8	12/ET	STUDY END
Study Week				0							1						4	16	34	52	
Study Day	Scr	-2	-1	0	1	2	3	4	5	6	7	8	9	12	17	21	28	112	240	365	
Visit Windows (Days)	-56, -3	0	0	0	0	0	0	0	0	0	0	0	0	±2	±2	±2	± 2	± 7	± 10	± 14	
		INPATIENT PERIOD																			
				VAC																	
**INVESTIGATIONAL PRODUCT**																					
Investigational Product/Placebo				X																	
**STUDY PROCEDURES**																					
Screening Assessment	X																				
Informed Consent	X																				
Assessment of Understanding	X																				
HIV Risk Assessment	X																			X	
HIV Risk Reduction Counseling	X			X										X	X	X	X	X	X	X	
HIV Test Counseling	X																X			X	PRN
Pregnancy Prevention Counseling	X			X										X	X	X	X				
Comprehensive Medical History	X																				
Intercurrent Illness		X	X	X										X	X	X	X	X			
Concomitant Medications/Supplements	X	X	X	X	X	X	X	X	X	X	X	X	X	X	X	X	X	X			
Complete Physical Exam	X																			X	
Abbreviated Physical Exam (see SOM)		X		X	X	X	X	X	X	X	X	X	X	X	X	X	X	X	X		
Weight	X												X							X	
Height	X																				
Vital Signs	X	X		BID	BID	BID	BID	BID	BID	BID	BID	BID	BID	X	X	X	X	X	X	X	
Reactogenicity Assessment				X	X	X	X	X	X	X	X	X	X								
Adverse Event Evaluation				X	X	X	X	X	X	X	X	X	X	X	X	X	X	X	SAE Only	SAE Only	
Social Impact Assessment																				X	
Unblinding																					X
**LABORATORY TESTS**																					
Syphilis, HBV, HCV	X																				
Urine Dipstick	X										X				X					X	
Urine Pregnancy Test	X			X														X			
Urine Toxicology Test	X	X																			
Complete Blood Count and Chemistry	X			X							X				X			X		X	
HIV Screening	X																				
HIV Diagnostics																	X			X	PRN
Ad26 Serology	X																X				
Vaccine Plasma PCR											X										
Humoral Assays				X											X		X	X	X	X	
Cellular Assays				X											X		X	X	X	X	
Mucosal Immunogenicity (rectal wick) - *optional*		X													X		X	X	X	X	
Shedding Specimens (rectal/throat swab)		X			X	X	X	X	X	X	X	X	X	X	X	X	X	prn qweek until neg			

18.6 Schedule of Procedures – Household Contacts

**Study Month**				**1**	**4**	**8**
**Study Week**		**2**	**3**	**4**	**16**	**34**
**Study Day**	**Scr**	**17** ***phone ok***	**21** ***phone ok***	**28**	**112**	**240**
**Visit Windows (Days)**	**-56, -3**	**±2**	**±2**	**+14**	**±14**	**± 14**
Screening Assessment	**X**					
Informed Consent	**X**					
Assessment of Understanding	**X**					
HIV Risk Assessment	**X**					
HIV Risk Reduction Counseling	**X**	**X**	**X**	**X**	**X**	**X**
HIV Test Counseling	**X**			**X**	**X**	**X**
Pregnancy Prevention Counseling	**X**	**X**	**X**	**X**	**X**	
Comprehensive Medical History	**X**					
Intercurrent Illness		**X**	**X**	**X**	**X**	
Adverse Event Evaluation		**X**	**X**	**X**	**X**	SAE Only
HIV Screening	**X**					
HIV Diagnostics				**X**	**X**	**X**
Ad26 Serology	**X**			**X**	**X**	**X**
Urine Pregnancy Test	**X**				**X**	

18.7 Blood Volumes (mL) - Volunteers

	Day:	Screen -56,-3	D0	D7 ±0	D17 ±2	D28 ±2	D112 ±7	D240 ±10	D365 ±14	
	Week:		W0	W1		W4	W16	W34	W52	
	Month:		M0			M1	M4	M8	M12	
**Procedure**	**Tube**		**Vaccine**							**Total**
Screening HIV test	SST	5								**5**
HIV diagnostic	EDTA					10			10	**20**
HBsAg/anti-HCV/ syphilis	SST	5								**5**
CBC with differential	EDTA	5	5	5	5		5		5	**30**
Chemistry panel*	SST	5	5	5	5		5		5	**30**
Ad26 serology	SST	5				5				**10**
Ad26 plasma PCR				5						**5**
Humoral assays	SST		20		10	10	10	10	10	**70**
Cellular assays	Na-Heparin		100		100	100	100	100	100	**600**
Epitope mapping	Na-Heparin					50				**50**
**Visit Total**		**25**	**130**	**15**	**120**	**175**	**120**	**110**	**130**	
**8 Week Total**		25	155	170	290	465	125	110	130	**825**

*Chemistry panel includes ALT, AST, and creatinine

18.8 Blood Volumes (mL) – Household Contacts

	Day:	Screen -56,-3	D28 +14	D112 ±14	D240 ±14	
	Week:		W4	W16	W34	
	Month:		M1	M4	M8	
**Procedure**	**Tube**					**Total**
Screening HIV test	SST	5				**5**
HIV diagnostic	EDTA		10	10	10	**30**
Ad26 serology	SST	5	5	5	5	**20**
**Visit Total**		**10**	**15**	**15**	**15**	**55**
**8 Week Total**		10	25	15	15	
